# Cardiometabolic Aspects of Congenital Adrenal Hyperplasia

**DOI:** 10.1210/endrev/bnae026

**Published:** 2024-09-06

**Authors:** Robert Krysiak, Hedi L Claahsen-van der Grinten, Nicole Reisch, Philippe Touraine, Henrik Falhammar

**Affiliations:** Department of Internal Medicine and Clinical Pharmacology, Medical University of Silesia, 40-555 Katowice, Poland; Amalia Children's Hospital, Radboud University Medical Centre, 6500 Nijmegen, The Netherlands; Medizinische Klinik und Poliklinik IV, LMU Klinikum München, 80336 Munich, Germany; Department of Endocrinology and Reproductive Medicine, Hôpital Pitié Salpêtrière, Sorbonne University Medicine, 75651 Paris, France; Department of Endocrinology, Karolinska University Hospital, SE-171 76 Stockholm, Sweden; Department of Molecular Medicine and Surgery, Karolinska Institutet, SE-171 76 Stockholm, Sweden

**Keywords:** androgens, atherosclerosis, blood pressure, cardiovascular disease, 21-hydroxylase deficiency, glucocorticoids, insulin resistance, risk factors

## Abstract

Treatment of classic congenital adrenal hyperplasia (CAH) is directed at replacing deficient hormones and reducing androgen excess. However, even in the era of early diagnosis and lifelong hormonal substitution, the presence of CAH is still associated with numerous complications and also with increased mortality. The aim of this article was to create an authoritative and balanced review concerning cardiometabolic risk in patients with CAH. The authors searched all major databases and scanned reference lists of all potentially eligible articles to find relevant articles. The risk was compared with that in other forms of adrenal insufficiency. The reviewed articles, most of which were published recently, provided conflicting results, which can be partially explained by differences in the inclusion criteria and treatment, small sample sizes, and gene–environment interactions. However, many studies showed that the presence of CAH is associated with an increased risk of weight gain, worsening of insulin sensitivity, high blood pressure, endothelial dysfunction, early atherosclerotic changes in the vascular wall, and left ventricular diastolic dysfunction. These complications were more consistently reported in patients with classic than nonclassic CAH and were in part related to hormonal and functional abnormalities associated with this disorder and/or to the impact of overtreatment and undertreatment. An analysis of available studies suggests that individuals with classic CAH are at increased cardiometabolic risk. Excess cardiovascular and metabolic morbidity is likely multifactorial, related to glucocorticoid overtreatment, imperfect adrenal hormone replacement therapy, androgen excess, and adrenomedullary failure. Cardiometabolic effects of new therapeutic approaches require future targeted studies.

## Graphical Abstract

**Graphical abstract:**
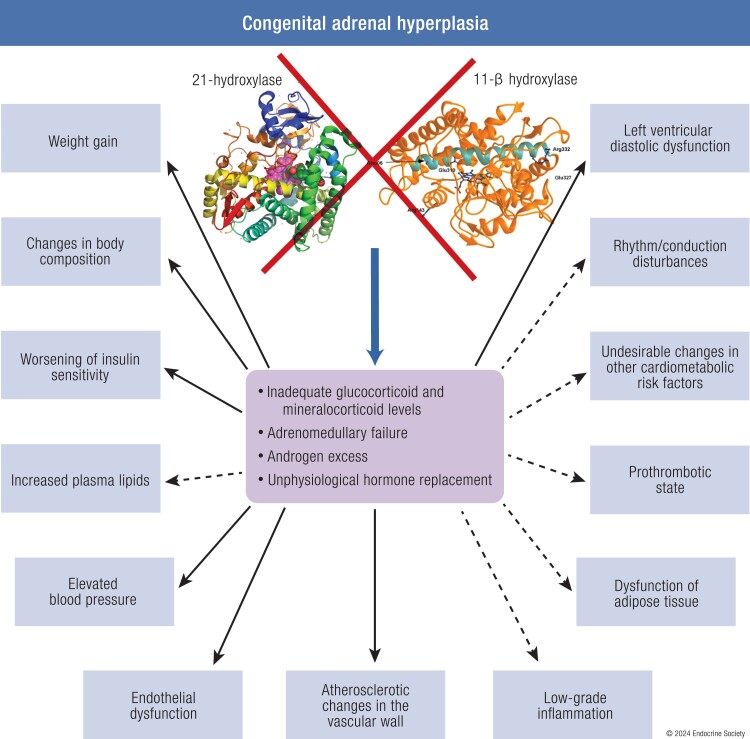

**Essential points**

- Congenital adrenal hyperplasia (CAH) seems to be associated with increased cardiometabolic risk
- Supraphysiological/unphysiological glucocorticoid replacement may increase the cardiometabolic risk
- Hyperandrogenism may also increase the cardiometabolic risk, especially in females
- Long acting/synthetic glucocorticoid preparations may be associate with a higher cardiometabolic risk than hydrocortisone, but the associations are uncertain
- The adrenomedullary failure in severe form of CAH may affect cardiometabolic risk but is unclear
- Thus, the pathogenesis of cardiometabolic complications in CAH is multifactorial and still not well understood

Congenital adrenal hyperplasia (CAH) refers to a group of disorders inherited in an autosomal recessive pattern. They are all characterized by cortisol deficiency resulting from a deficiency in 1 or more enzymes involved in adrenal steroidogenesis. Due to a lack of negative feedback system this causes compensatory increased adrenocorticotropic hormone (ACTH) secretion from the pituitary gland (1). The most common enzyme defect, accounting for 95% to 99% of all cases, is 21-hydroxylase deficiency (21OHD), interrupting conversion of 17-hydroxyprogesterone (17OHP) to 11-deoxycortisol and of progesterone to deoxycorticosterone (2-5). Less frequent causes of CAH include deficiencies of 11β-hydroxylase, 3β-hydroxysteroid dehydrogenase, 17α-hydroxylase/17,20-lyase, steroidogenic acute regulatory protein, and P450 oxidoreductase (6-10). Only 21OHD, 11β-hydroxylase deficiency, and, though to a much lesser extent, 3β-hydroxysteroid dehydrogenase deficiency (in females) lead to increased androgen production (1). Unless otherwise stated, CAH hereinafter refers to CAH caused by 21OHD. The elevated ACTH concentration in 21OHD results in adrenocortical growth (hyperplasia) and accumulation of steroid precursors before the enzymatic block which is shunted towards increased androgen production (11). Bioactive androgens are additionally produced in target tissues via “the backdoor pathway” and in the 11-oxoandrogen pathway (12).

CAH is divided into classic CAH (C-CAH), and nonclassic CAH (NC-CAH). Based on residual enzymatic activity of 21-hydroxylase and the ability to produce adequate amounts of aldosterone, C-CAH is further divided into the salt-wasting phenotype (SW-CAH) with a lack of both cortisol and aldosterone and the simple virilizing phenotype (SV-CAH) in which the aldosterone concentration is generally enough to prevent salt-wasting crisis (11). In NC-CAH the residual enzymatic activity is 30% to 50%. This subgroup is characterized by a generally normal cortisol and aldosterone production but still elevated ACTH and adrenal androgen production (13). As calculated by worldwide screening of newborns, the prevalence of the classic form ranges from 1:6000 to 1:27 000, of which 75% have SW-CAH (1, 14). The estimated prevalence of NC-CAH varies between 1 case per 200 persons to 1 case per 1000 persons, and between 0.6% and 7.1% in women with androgen excess (14, 15).

SW-CAH is characterized by both cortisol and aldosterone deficiency, as well by marked androgen excess, occurring already in fetal life (1). Aldosterone deficiency can cause life-threatening salt-wasting crisis in the neonatal period (1). Adrenal androgen excess during the first trimester of gestation causes virilization of the external genitalia and consequently atypical external genitalia in affected 46,XX individuals (11, 16). Some mineralocorticoid production prevents salt-wasting crisis in children with SV-CAH and, if not detected by neonatal screening or virilized genitalia in the newborn 46,XX individuals, this form is diagnosed during childhood because of signs of precocious puberty, accelerated linear growth, and advanced bone age with decreased final height. Because of only mild enzyme impairment, NC-CAH is mostly diagnosed in later childhood, adolescence, or adult life (13). Children with NC-CAH usually present with signs of moderate hyperandrogenism such premature pubarche and advanced bone age while adolescents and adult women usually present with hirsutism, menstrual cycle disorders, acne, and impaired fertility (17). In males, diagnosis of NC-CAH is mainly the result of family screening or of finding adrenal incidentaloma, but most cases are probably never diagnosed (13, 18). Although cortisol production is sufficient during basal conditions, some patients with NC-CAH have suboptimal cortisol concentrations in response to ACTH stimulation (13).

A useful tool in 21OHD is genotyping, which is helpful for confirming the diagnosis, genetic counseling, and prenatal diagnosis (19). Based on residual 21-hydroxylase activity *CYP21A2* variants can be classified into 5 groups: null (associated with SW-CAH), IVS2-13A/C>G (also known as I2 splice or I2G, often associated with SV-CAH), I172N (usually associated with SV-CAH), P30L (sometimes SV-CAH, sometimes NC-CAH), and V281L together with P453S (normally associated with NC-CAH), with in vitro activity of the enzyme from 0% (null) to 30% to 50% (V281L and P453S) (20). Although the genotype–phenotype correlation is far from perfect, this classification is very useful both in clinical work and research.

Unlike other forms of adrenal insufficiency (AI) in which adrenal androgens and androgen precursors are not chronically elevated, glucocorticoid treatment in 21OHD is aimed not only at replacing deficient hormone but also at reducing ACTH secretion and at normalizing adrenal androgen excess. Adult patients with CAH may be treated with either hydrocortisone or with synthetic glucocorticoids: prednisone, prednisolone, and dexamethasone, which substitute for the lack of endogenous cortisol and are effective in suppressing the production of adrenal androgens (21). Long-acting glucocorticoids are, however, contraindicated in children because of the negative effect on growth velocity during infancy, childhood, and puberty (2, 22). Unfortunately, glucocorticoid replacement therapy fails to mimic the physiological cortisol rhythm and is associated with the risk of both undertreatment and overtreatment, also within 1 day, which can result in several complications (21, 23). Due to mineralocorticoid resistance, antimineralocorticoid effects of elevated 17OHP, immature kidneys, and low-sodium diet, the doses of fludrocortisone are relatively high in the first months of life (24), and therefore infants with SW-CAH usually require transient additional salt supplementation in the first months of life (25). Later, fludrocortisone dose can be substantially reduced and some, even initially diagnosed with SW-CAH, may not require mineralocorticoid replacement in older age (1). Although most subjects with NC-CAH do not require lifelong adrenal hormone replacement therapy in everyday life, some may require stress dosing due to mild partial AI. Moreover, many individuals with NC-CAH are treated with glucocorticoids to alleviate the consequences of adrenal androgen excess, and in case of a wish to conceive (13, 17, 26).

Neonatal screening programs, assessing 17OHP concentrations, facilitate early diagnosis and treatment of C-CAH, reduce mortality and morbidity, and prevent unfavorable outcomes (27). Thanks to neonatal screening program in most Western countries late diagnosis of C-CAH has almost disappeared in those countries (28).

Long-term glucocorticoid therapy, especially overtreatment during childhood leads to growth retardation, resulting in reduced final height in adults although undertreatment with elevated androgens also leads to short stature in adults. Overtreatment with glucocorticoids is also responsible for a reduction in bone mineral density, associated with increased risk of bone fractures (29-31). Both women and men with C-CAH have lower fertility rates (32-34). The therapeutic challenges of balancing androgen and progesterone excess (undertreatment) and glucocorticoid excess (overtreatment) accentuate the many complexities of CAH treatment which, despite current hormone replacement therapy, is associated with adverse clinical consequences.

Cardiometabolic risk refers to a constellation of medical conditions and factors (both dichotomous and continuous) that increase the lifetime possibility of experiencing vascular events (acute coronary syndromes and stroke) or developing diabetes (35). This term encompasses both traditional risk factors, integrated in risk calculators (hypertension, dyslipidemia, smoking, old age, male sex, race, and a positive history of early coronary events), as well as novel or emerging ones (abdominal obesity, impaired insulin sensitivity, impaired endothelial function, low-grade systemic inflammation, hormonal dysfunction of adipose tissue, prothrombotic profile, and structural changes in the vascular system). Each of these factors is associated with increased morbidity and mortality, and the global risk is particularly high in individuals with many factors coexisting with one another (36). Most individuals remain asymptomatic for many years. Hence, early identification and treatment of individuals at high risk for cardiovascular/cardiometabolic disorders could lead to changes in modifiable risk factors and more favorable outcomes because these disorders are the leading cause of preventable death worldwide, and often result in chronic disability (35, 37).

Thus, it seems justified to summarize the current stage of knowledge concerning cardiometabolic aspects in adults and children with CAH, which is the aim of our review. The term “cardiometabolic aspects” refers in the manuscript to modifiable cardiometabolic risk factors, structural changes in the vascular system, and definitive, clinically relevant outcomes (heart disorders, vascular diseases and events, stroke, and diabetes).

## Search Strategy

The established databases, including PubMed, EMBASE, and Cochrane Library, were searched in order to identify potentially eligible articles published between January 1966 and October 2023. Key terms of the search included congenital adrenal hyperplasia and 21OHD, combined with Medical Subject Headings terms for major topics such as AI, cardiovascular and cerebrovascular disease, ischemic heart disease (coronary artery disease, coronary heart disease), myocardial infarction, stroke, heart failure (heart insufficiency, cardiac failure, cardiac insufficiency), thromboembolism, pulmonary embolism, peripheral vascular disease, hypertension, obesity, overweight, diabetes, dyslipidemia, systemic inflammation, adipokines, hemostasis, vascular wall, atherosclerosis, atrial fibrillation, rhythm disturbances (arrhythmia), conduction disturbances, and cardiac function. Then, reference lists of all obtained articles were scanned to find other relevant papers that were missed during indexing. Of 125 eligible articles, 119 (95%) were reviewed and included in the current review. We excluded 3 papers written in languages other than English or languages understood by the authors (Chinese n = 2, Russian n = 1), 2 articles including individuals with disorders of sex development but without a separate analysis for patients with CAH, and 1 article with inconsistencies in the presented results. Characteristics of patients participating in the reviewed studies (3, 25, 38-154) are shown in Table 1. Similar to other rare disorders, the reported studies are mostly retrospective, and inconsistent regarding patient populations, inclusion criteria, treatment strategies, and outcome measures. They are also flawed by small sample sizes, sometimes lack of matched control groups, and lack of information about confounding variables. Despite all these drawbacks, the very broad range of included studies enables to provide a representative picture of the association between CAH and cardiometabolic health and disease. Although the results of all studies have been taken into consideration, a special emphasis was put on articles published over the last 4 years, on comparisons between cardiometabolic aspects of CAH and other forms of AI, and on issues not discussed in detail in a previous paper reviewing theoretical and clinical aspects of CAH (1).

**Table 1.** Characteristics of studied individuals with congenital adrenal hyperplasia due to 21-hydroxylase deficiency and controls according to the PICO format

Authors	Participants	Intervention	Controls	Outcomes	Study design	Study limitations
Abdel Meguid et al (38)	30 patients with C-CAH (11 HC-treated; 19 PDL-treated) F/M: 19/11 Age: 6.7 ± 2.3	G + FC HCED: HC: 15.2 ± 3.7 mg/m^2^; PDL: 21.0 ± 5.2 mg/m^2^ FCD: 100-150 μg^*e*^	66 (age-matched obese with BMI SDS >2)	Body mass, glucose homeostasis markers, lipids, blood pressure, intima–media thickness	Case–control	Small sample size, retrospective nature, sampling bias, recall bias, observation bias, confounding bias
Ahmed et al (39)	30 patients with C-CAH (8 HC-treated; 22 PD-treated) F/M: 19/11 Age: HC-treated patients: 7.4 ± 3.3; PD-treated patients: 6.4 ± 2.7	G + FC GD: HC: 15.2 ± 3.7 mg/m^2^; PD: 5.5 ± 1.1 mg/m^2^ (HCED: 27.5 ± 5.5 mg/m^2^) FCD: 100-150 μg^*e*^	30 (age-, and sex- matched healthy)	Body mass, glucose homeostasis markers, lipids, blood pressure, intima–media thickness	Case–control	Small sample size, retrospective nature, sampling bias, recall bias, observation bias, confounding bias
Akyürek et al (40)	25 patients with SW F/M: 16/9 Age: 9.1 ± 4.1	G + FC HCED: 15-18 mg/m^2^ FCD: 100-150 μg/m^2*e*^	25 (age- and sex-matched healthy)	Body mass, glucose homeostasis markers, lipids, blood pressure, intima–media thickness	Case–control	Small sample size, retrospective nature, sampling bias, recall bias, observation bias, confounding bias
Amr et al (41)	32 patients with C-CAH (SW: 24, SV: 8) F/M: 24/8 Age: 9.6 (6.5-10.1)^*b*^	G + FC HCED: 10-15 mg/m^2*e*^ FCD: 50-100μg/m^2*e*^	32 (age- and sex-matched healthy)	Body mass, glucose homeostasis markers, lipids, blood pressure, intima–media thickness	Case–control	Small sample size, retrospective nature, sampling bias, recall bias, observation bias, confounding bias
Amr et al (42)	47 patients with C-CAH (SW: 39, SV: 8) F/M: 16/31 Age: 6 (3.95-12)^*b*^	G + FC HCED: treated < 6 years: 12.6 ± 4.7 mg; treated > 6 years: 15.9 ± 4.0 mg FCD: 89.3 ± 44.3 μg	47 (age-, sex-, and body area-matched)	Blood pressure, echocardiogram	Case–control	Small sample size, retrospective nature, sampling bias, recall bias, observation bias, confounding bias
Apsan et al (43)	35 patients with C-CAH (SW: 25; SV: 10) F/M: 20/15 Age: treated twice daily: 6.0 (5.0-7.0)^*b*^; treated 3 times daily: 7.0 (5.5-8.0)^*b*^	G (NI about FC) HCED: mean dose: treated twice daily—12.1 mg/m^2^, treated 3 times daily—11.7 mg/m^2^	—	Body mass, blood pressure	Retrospective chart analysis	Small sample size, retrospective nature, sampling bias, observation bias, confounding bias
Ariyawatkul et al (44)	21 patients with C-CAH (SW: 10, SV: 11) F/M: 17/4 Age: 15.2 ± 5.8	G + FC HCED: 21.4 ± 5.8 mg/m^2^ FCD: 50-150 μg^*e*^	21	Body mass, glucose homeostasis markers, lipids, systemic inflammation markers, leptin	Case–control	Small sample size, retrospective nature, sampling bias, recall bias, observation bias, confounding bias
Arlt et al (3)	203 patients with CAH (21OHD: 199 [C-CAH: 168, NC: 31]; 11OHD: 3; 3HSD: 1) F/M: 137/65 Age: C-CAH women: 33 (18-66)^*d*^; NC women: 43 (22-69)^*d*^; C-CAH men: 32 (18-56)^*d*^; NC men: 19, 36 and 36	G + FC GD: depending on drug and form of CAH—median between: 20-25 mg^*e*^ (HC), 5-7.5^*e*^ mg (PDL), 0.25-0.5 mg^*e*^ (DXM) FCD: depending on drug and form of CAH—median between 50 and 150 μg	—	Body mass, glucose homeostasis markers, lipids, blood pressure	Cross-sectional	Sampling bias, inability to establish causality, recall bias
Auer et al (45)	40 patients with SW F/M: 22/18 Age: HC: 36.5 (23.7-41.8)^*b*^ SG: 28.5 (22.3-39.8)^*b*^	G + FC HCED: HC-treated: 15.3 (13.8-18.4)^*b*^ mg/m^2^; SG-treated: 17.0 (12.1-18.9)^*b*^ mg/m^2^ FCD: HC-treated: 75 (50-100)^*b*^ μg; SG-treated: 100 (50-100)^*b*^ μg	—	Blood pressure	Cross-sectional	Sampling bias, inability to establish causality, recall bias
Bachelot et al (46)	45 patients with CAH (SW: 23, SV: 12, NC: 10) F/M: 36/9 Age: SW: 27.5 ± 1.1; SV: 35.5 ± 2.2; NC: 25.9 ± 2.3	G + FC HCED: SW: 18.3 ± 0.9 mg/m^2^; SV: 17.2 ± 1.1 mg/m^2^; NC: 14.7 ± 2.2 mg/m^2^ FCD: SW: 81.2 ± 7.5 μg/m^2^; SV: 81.2 ± 11.9 μg/m^2^; NC: NT	—	Body mass, glucose homeostasis markers	Cross-sectional	Sampling bias, inability to establish causality, recall bias
Bachelot et al (47)	104 patients with CAH (SW: 53, SV: 17, NC: 34) F/M: 71/33 Age: 27.9 (16-52)	G + FC GD: NI FCD: NI	—	Body mass	Cross-sectional	Sampling bias, inability to establish causality, recall bias
Bacila et al (48)	101 patients with C-CAH F/M: 54/47 Age: 12.4 (10.3-15.1)^*d*^	G + FC HCED: 13.6 ± 3.6 mg/m^2^ FCD: 105.6 ± 49.3 μg/m^2^	83	Body mass, glucose homeostasis markers, lipids, blood pressure	Cross-sectional	Sampling bias, inability to establish causality, recall bias
Bayraktar et al (49)	50 patients with NC F/M: 50/0 Age: 22.1 ± 2.9	NI	25 control F, 50 PCOS	Glucose homeostasis markers, lipids, homocysteine	Case–control	Small sample size, retrospective nature, sampling bias, recall bias, observation bias, confounding bias
Ben Simon et al (50)	75 patients with NC F/M: 49/26 Age: 11.2 (8.2-14.7)^*b*^	G in 61 patients (81%) HCED: median dose: 6.59 mg/m^2^; lifetime cumulative dose: 7.62^*b*^ (2.55-12.90) g/m^2^	134 (age-, and sex-matched)	Body mass and composition, glucose homeostasis markers, lipids, blood pressure	Cohort	Confounding bias, recall bias, losses to follow up
Bonfig et al (51)	716 patients with C-CAH (SW: 571, SV: 145) F/M: 401/315 Age: 9.2 ± 3.9	G + FC HCED: 14.4 ± 6.4 mg/m^2^ FCD: 72.7 ± 38.8 μg/m^2^	—	Body mass, blood pressure	Cohort	Confounding bias, recall bias, losses to follow up
Borges et al (52)	23 patients with C-CAH (SW: 12, SV: 11) F/M: 16/7 Age: M: 23.1 ± 4.0; F: 22.9 ± 3.6	G + FC HCED: M: 12.8 ± 2.8 mg/m^2^; F: 13.5 ± 4.8 mg/m^2^ FCD: NI	20 (age-, sex-, ethnicity-, and physical activity- matched)	Body mass and composition, glucose homeostasis markers, lipids, leptin, adiponectin	Case–control	Small sample size, retrospective nature, sampling bias, recall bias, observation bias, confounding bias
Borges et al (53)	20 patients with C-CAH (SW: 12, SV: 8) F/M: 15/5 Age: M: 23.6 ± 4.3; F: 23.7 ± 3.6	G + FC HCED: M 12.5 ± 2.6 mg/m^2^; F: 13.1 ± 4.7 mg/m^2^ FCD: NI	16 (age-, sex-, ethnicity-, and physical activity- matched)	Body mass and composition, glucose homeostasis markers, blood pressure, intima–media thickness, leptin, adiponectin, echocardiogram	Case–control	Small sample size, retrospective nature, sampling bias, recall bias, observation bias, confounding bias
Borges et al (54)	18 patients with C-CAH (SW: 7, SV: 11) F/M: 12/6 Age: M: 24.0 ± 3.6; F: 23.5 ± 3.3	G + FC HCED: 13.9 ± 4.4 mg/m^2^ FCD: 25-100 μg^*e*^	19 (age-, sex-, ethnicity-, and physical activity- matched)	Body mass and composition, glucose homeostasis markers, blood pressure	Case–control	Small sample size, retrospective nature, sampling bias, recall bias, observation bias, confounding bias
Botero et al (55)	14 F/M: 10/4 Age: 1.1-10.0^*e*^	G + FC HCED: 10-20 mg/m^2^ (all used PD) FCD: NI	14	Lipids	Case–control	Small sample size, retrospective nature, sampling bias, recall bias, observation bias, confounding bias
Bouvattier et al (56)	219 patients with C-CAH (SW: 161, SV: 58) F/M: 0/219 Age: 32.1 ± 10.2	G + FC GD: SW: HC—27.7 ± 0.5 mg, DXM: 0.41 ± 0.29 mg, PD: NI; SV: HC—21.8 ± 1.1 mg FCD: 112.1 ± 3.6 μg	**—**	Body mass, glucose homeostasis markers, lipids, blood pressure	Cross-sectional	Sampling bias, inability to establish causality, recall bias
Cameron et al (57)	21 patients with C-CAH (SW: 18, SV: 3) F/M: 8/13 Age: 8-32^*e*^	G + FC GD: NI FCD: 17.4 ± 6.1 μg	21	Body mass	Cross-sectional	Small sample size, sampling bias, inability to establish causality, recall bias
Charmandari et al (58)	18 patients with CAH (SW: 12, SV: 4, 11OHD: 2) F/M: 6/12 Age: 7.2 ± 0.7	G + FC HCED: 21OHD: 14.8 ± 4.2 mg/m^2^; 11OHD: 18.3 and 20.3 mg/m^2^ FCD: 114 ± 48 μg (21OHD)	28	Glucose homeostasis markers, leptin	Retrospective cross-sectional analysis	Retrospective nature, sampling bias, recall bias, observation bias, confounding bias
Charoensri and Auchus (59)	254 patients with CAH (C-CAH: 147, NC: 107) F/M: 186/68 Age: 35 (28.25-46)^*b*^	G + FC HCED: 19.375 (0-30)^*b*^ mg FCD: C-CAH: M—100 (100-200)^*b*^ μg, F: 100 (50-150)^*b*^ μg; NC: 0 (0-0) μg^*b*^	—	Cardiovascular morbidity, body mass, lipids, blood pressure	Retrospective cross-sectional analysis	Retrospective nature, sampling bias, recall bias, observation bias, confounding bias
Christiansen et al (60)	18 patients with C-CAH (SW: 17, SV: 1) F/M: 8/10 Age: 18-33^*e*^	G + FC GD: NI FCD: NI	120	Fat content	Cross-sectional	Small sample size, sampling bias, inability to establish causality, recall bias
de Oliveira et al (61)	30 patients with C-CAH (SW: 15, SV: 15) F/M: 20/10 Age: 23.0 ± 3.7	G (NI about FC) HCED: 13.0 ± 4.7 mg/m^2^	21	Glucose homeostasis markers, lipids, blood pressure	Cross-sectional	small sample size, sampling bias, inability to establish causality, recall bias
de Oliveira et al (62)	22 patients with C-CAH F/M: 15/7 Age: F: 22.9 ± 3.7; M: 23.8 ± 4.5	G (NI about FC) HCED: F: 13.2 ± 4.8 mg/m^2^; M: 12.5 ± 2.9 mg/m^2^	17 (healthy)	Fat content	Case–control	Small sample size, retrospective nature, sampling bias, recall bias, observation bias, confounding bias
de Silva et al (63)	11 patients with CAH (SW: 5, SV: 4, 11OHD: 1, LAH: 1) F/M: 7/4 Age: 14.5 (8.5-27.2)^*c*^	G + FC HCED: 12.8 (3.6-30.3)^*c*^ mg/m^2^ FCD: 150 (100-250)^*c*^ μg	**—**	Glucose homeostasis markers, blood pressure	Prospective uncontrolled	Very small sample size, sampling bias, recall bias, observation bias, confounding bias
de Vries et al (64)	114 patients with all NC F/M: 92/22 Age: 7.9 ± 4.2	G HCED: 9.2 ± 4.5 mg/m^2^	**—**	Fat content, lipids	Retrospective cross-sectional	Retrospective nature, sampling bias, inability to establish causality, recall bias
Debor et al (65)	53 patients with C-CAH F/M: 24/29 Age: 5 months-11 years^*e*^	G (NI about FC) HCED: M: 12.5 (10-15)^*d*^ mg/m^2^; F: 12.1 (10-15)^*d*^ mg/m^2^	—	Body mass	Retrospective analysis	Retrospective nature, sampling bias, inability to establish causality, recall bias
Delai et al (66)	30 patients with NC F/M: 25/5 Age: 24 ± 10	G HCED: NC/NC group: 5.8 ± 2.1 mg/m^2^; C/NC group: 4.4 ± 3.3 mg/m^2^	25 (sex-, and BMI-matched healthy)	Fat distribution, glucose homeostasis markers, blood pressure, intima–media thickness, systemic inflammation markers, leptin	Case–control	Small sample size, retrospective nature, sampling bias, recall bias, observation bias, confounding bias
Dereli et al (67)	50 patients with NC F/M: 50/0 Age: 22.1 ± 3.2	NI	30 control F, 50 PCOS		Prospective uncontrolled	Sampling bias, uncontrolled, recall bias, observation bias, confounding bias
Dubinski et al (68)	10 patients with C-CAH F/M: 4/6 Age: 10.9 ± 3.3	G + FC HCED: 14.1 (7.1-21.0)^*c*^ mg/m^2^ FCD: 58 ± 28 μg	—	Glucose homeostasis markers	Cohort	Very small sample size, sampling bias, recall bias, observation bias, confounding bias
Espinosa-Reyes et al (69)	22 patients with CAH (SW: 8, SV: 5, NC: 9) F/M: 18/4 Age: 17.2 ± 5.7	G + FC HCED: SW: 37.5 (15-40)^*d*^ mg; SV: 27.5 (15-50)^*d*^ mg; NC: 15 (15-17.5)^*d*^ mg FCD: NI	22 (healthy matched for age, sex, BMI, and pubertal status)	Fat content, glucose homeostasis markers, lipids, blood pressure, endothelial function, intima–media thickness	Case–control	Small sample size, retrospective nature, sampling bias, recall bias, observation bias, confounding bias
Falhammar et al (70, 71)	61 patients with CAH (SW: 27, SV: 28, NC: 6) F/M: 61/0 Age: 30 (18-63)^*d*^	G + FC GD: HC: 33.3 ± 2.1 mg; PD: 6.3 ± 0.3 mg; CA: 40 ± 2.5 mg; DXM: 0.55 ± 0.08 mg FCD: 90 ± 10 μg	61	Body mass and composition, glucose homeostasis markers, lipids, blood pressure	Cohort	Confounding bias, recall bias, losses to follow up
Falhammar et al (72, 73)	30 patients with CAH (SW: 17, SV: 11, NC: 2) F/M: 0/30 Age: 35.7 ± 11.4	G + FC HCED: 17.4 ± 5.2 mg/m^2^ FCD: 110 ± 60 μg^*e*^	30/32	Body mass and composition, glucose homeostasis markers, lipids, blood pressure, homocysteine, 25-hydroxyvitamin D, heart rhythm	Cohort	Confounding bias, recall bias, losses to follow up
Falhammar et al (74)	588 patients with CAH (SW: 240, SV: 167, NC: 75, unknown: 106) F/M: 335/253 Age: 26 (0-92)^*d*^	NI	58 800	Cardiovascular and metabolic morbidity, body mass, glucose homeostasis markers, lipids, blood pressure	Cohort	Confounding bias, recall bias, losses to follow up
Falhammar et al (75)	226 (SW: patients with CAH 111, SW: 66, NC: 34, unknown phenotype 21OHD: 3, 11OHD: 6, 3HSD: 2, POR: 2, LAH: 1, unknown: 1) F/M: 221/5 Age: 30.4 ± 11.4	NI	>200 000	Cardiovascular and metabolic morbidity, body mass glucose homeostasis markers, lipids, blood pressure, 25-hydroxyvitamin D	Cross-sectional	Sampling bias, inability to establish causality, recall bias
Farghaly et al (76)	40 patients with C-CAH (SW: 30, SV: 10) F/M: 28/12 Age: 14.8 ± 2.6	G + FC GD: NI FCD: 50-100 μg/m^2*e*^	40 (healthy matched for age, sex, pubertal status, and socioeconomical status)	Body mass, glucose homeostasis markers, lipids, blood pressure, endothelial function, intima–media thickness, systemic inflammation markers	Case–control	Small sample size, retrospective nature, sampling bias, recall bias, observation bias, confounding bias
Finkielstain et al (77)	244 patients with C-CAH (SW: 123, SV: 60, NC: 61) F/M: 132/112 Age: children: 8.0 ± 5.5-10.5 ± 3.0; adults: 29.5 ± 12.5-34.3 ± 13.7	G (NI about FC) HCED: C-CAH—children: 15.0 ± 5.9 mg/m^2^; adults: 17.9 ± 7.6 mg/m^2^; NC-CAH: NI	—	Body mass, glucose homeostasis markers, lipids, blood pressure, 25-hydroxyvitamin D	Cross-sectional study	Sampling bias, inability to establish causality, recall bias
Gasparini et al (78)	21 patients with SW F/M: 15/6 Age: 25 (6-60 days)	NI	—	Platelet count	Uncontrolled	Small sample size, no control group, retrospective nature, sampling bias, recall bias, observation bias, confounding bias
Girgis and Winter (79)	28 patients with CAH (SW: 23, SV: 3, 17OHD: 2) F/M: 16/12 Age: tight control: 10.7 ± 4.8; fair control: 14.3 ± 3.5; poor control: 12.6 ± 4.2	HC + FC HCED: 10-15 mg/m^2^ FCD: 100-200 μg^*e*^	—	25-hydroxyvitamin D	Uncontrolled	Small sample size, no control group, retrospective nature, sampling bias, recall bias, observation bias, confounding bias
Green-Golan et al (80)	6 patients with C-CAH F/M: 3/3 Age: 17.3 ± 1.5	G + FC HCED: 15.7 (13.2-17.9) mg/m^2^ FCD: 105 (75-150) μg^*c*^	7 (age-, sex-, and BMI-matched healthy)	Glucose homeostasis markers, blood pressure, heart rhythm, exercise test	Case–control	Very small sample size, retrospective nature, sampling bias, recall bias, observation bias, confounding bias
Hagenfeldt et al (81)	13 patients with C-CAH F/M: 13/0 Age: 23.9 ± 0.8	G + FC GD: DXM: 0.5-0.7 mg^*e*^, PDL: 5.6-12.5 mg^*e*^, CA: 37.5 mg, TML: 8 mg, CA: 15 mg + PDL: 3.75 mg FCD: 75-150 μ^*e*^	12 (of similar age)	Body mass and composition	Case–control	Small sample size, retrospective nature, sampling bias, recall bias, observation bias, confounding bias
Han et al (82)	196 patients with CAH F/M: 131/65 Age: 34.4 ± 11.3	G (NI about FC) GD: NI	—	Body mass and composition, glucose homeostasis markers,	Cross-sectional study	Sampling bias, inability to establish causality, recall bias
Harrington et al (83)	14 patients with C-CAH (SW: 11, SV: 3) F/M: 7/7 Age: 14.8 ± 3.2	G + FC HCED: 13.3 ± 4.1 mg/m^2^ FCD: 108.3 ± 19.5 μg/m^2^	53 healthy and 28 obese children	Body mass and composition, glucose homeostasis markers, lipids, blood pressure, endothelial function, intima–media thickness	Case–control	Retrospective nature, case-controlled design despite claims about cross-controlled design sampling bias, recall bias, observation bias, confounding bias
Hashemi Dehkordi et al (84)	78 patients with C-CAH (SW: 51, SV: 27) F/M: 41/37 Age: 9.4 ± 4.1	G (NI about FC) HCED: 10-20 mg/m^2*e*^	—	Body mass, glucose homeostasis markers, blood pressure	Uncontrolled study	Uncontrolled design, retrospective nature, sampling bias, recall bias, observation bias, confounding bias
Hoepffner et al (85)	34 patients with C-CAH (SW: 28, SV: 4): 23 children; 11 adults F/M: 23/11 Age: children/adolescent: 13.2 ± 3.2; adults: 22.9 ± 2.9	G + FC HCED: children/adolescents: 18.7 ± 6.8 mg/m^2^; adults: 23.4 ± 7.2 mg/m^2^ FCD: children/adolescents: 70.85 ± 22.4 μg; adults: 104.5 ± 33.7 μg	—	Blood pressure	Cohort study	Uncontrolled design, sampling bias, recall bias, observation bias, confounding bias
Janus et al (86)	70 patients with CAH (SW: 51, SV: 10, NC: 9) F/M: 43/27 Age: 3.0-17.9	G + FC HCED: SW: 17.2 ± 4.2 mg/m^2^; SV: 19.5 ± 2.5 mg/m^2^; NC: 11.9 ± 3.5 mg/m^2^ FCD: SW: 66.5 ± 36.5 μg/m^2^; SV: 28.6 ± 15.5 μg/m^2^	—	Fat content, ambulatory blood pressure monitoring	Uncontrolled study	Uncontrolled design, retrospective nature, sampling bias, recall bias, observation bias, confounding bias
Jenkins-Jones et al (87)	255 patients with CAH F/M: 132/123 Age: children: 5.1 ± 5.0; adults: 35.4 ± 14.2	NI	2550	All-cause mortality, body mass, blood pressure	Retrospective, matched-cohort	Nonexposed controls, retrospective nature, sampling bias, recall bias, observation bias, confounding bias
Kara et al (88)	41 patients with C-CAH F/M: 34/7 Age: 30 ± 8	G (NI about FC) HCED: 17 ± 9 mg	38 (age-, sex-, and BMI-matched healthy)	Body composition, glucose homeostasis markers, lipids, blood pressure	Case–control	Small sample size, retrospective nature, sampling bias, recall bias, observation bias, confounding bias
Kępczyńska-Nyk et al (89)	21 patients with CAH (SW: 17, SV: 3, 11OHD: 1) F/M: 21/0 Age: 29.3 ± 7.6	G + FC GD: HC-treated: 30-40 mg^*e*^; PD-treated: 5-10 mg^*e*^ FCD: 500 μg	20 healthy F, 63 PCOS	Body mass and composition, glucose homeostasis markers, lipids	Case–control	Small sample size, retrospective nature, sampling bias, recall bias, observation bias, confounding bias
Kim et al (90, 91)	28 patients with C-CAH (SW: 20, SV: 8) F/M: 15/13 Age: 15.6 ± 3.2	G + FC HCED: 19.5 ± 5.4 mg/m^2^ FCD: 100 ± 50 μg	28/20 (healthy matched for age, sex, pubertal stage ethnicity, and BMI	Body mass and composition, glucose homeostasis markers, lipids, blood pressure, intima–media thickness, plasminogen activator inhibitor-1	Cross-sectional	Small sample size, sampling bias, inability to establish causality, recall bias
Koetz et al (92)	41 patients with CAH (SW: 20, SV: 13, NC: 6, 11OHD: 1, unknown: 1) F/M: 27/14 Age: 45.6 ± 12.4	G + FC HCED: 15.5 ± 7.8 mg/m^2^ FCD: 50-100 μg^*e*^	81 primary adrenal insufficiency	Body mass	Cross-sectional	Sampling bias, inability to establish causality, recall bias
Korkmaz et al (93)	25 patients with CAH F/M: 16/9 Age: 9.4 (1.5-16.8)^*d*^	G + FC HCED: 18.8 (9.2-23.9) mg/m^2*d*^ FCD: 100 μg	25 (age-, sex-, and body size-matched)	Glucose homeostasis markers, lipids, blood pressure	Case–control	Small sample size, retrospective nature, sampling bias, recall bias, observation bias, confounding bias
Kroese et al (94)	12 patients with CAH (SW: 8, SV: 3, NC: 1) F/M: 7/5 Age: 37.5 ± 8.9	G + FC HCED: 23.1 ± 8.0 mg FCD: 122 ± 118 μg	12	Fat content and distribution, glucose homeostasis markers, blood pressure, heart rhythm	Randomized, placebo controlled, crossover trial	Very small sample size, short treatment period
Krysiak et al (95)	8 patients with NC F/M: 8/0 Age: 34 ± 5	Metformin	10 (age- and weight-matched)	Glucose homeostasis, lipids	Cohort	Small sample size, no information about confounding factors
Krysiak et al (96)	8 patients with NC F/M: 8/0 Age: 38 ± 5	Metformin + Simvastatin	12 (age-, weight-, glucose- and lipid-matched)	Glucose homeostasis, lipids	Cohort	Small sample size, no information about confounding factors
Krysiak et al (97)	12 patients with NC F/M: 12/0 Age: atorvastatin-treated: 31 ± 6; untreated: 32 ± 6	Atorvastatin	—	Glucose homeostasis, lipids, uric acid, hsCRP, fibrinogen, homocysteine, 25-hydroxyvitamin D	Cohort	Small sample size, no information about confounding factors, lack of matched control group
Krysiak et al (98)	14 patients with NC F/M: 14/0 Age: 30 ± 5	NT	20 (age- and weight-matched)	Glucose homeostasis, lipids, uric acid, hsCRP, fibrinogen, homocysteine, 25-hydroxyvitamin D	Case–control	Retrospective, small sample size, no information about confounding factors
Kurnaz et al (99)	56 patients with NC F/M: 29/27 Age: prepubertal group: 7.0 ± 0.5; postpubertal F: 14.6 ± 2.3; postpubertal M: 15.9 ± 2.1	G + F HCED: prepubertal group: 12.1 ± 3.7 mg/m^2^; postpubertal F: 14.1 ± 8.6 mg/m^2^; postpubertal M: 16.5 ± 6.0 mg/m^2^ FCD: prepubertal group: 56.7 ± 54.7 μg/m^2^; postpubertal F:37.5 ± 27.1 μg/m^2^; postpubertal M: 31.7 ± 26.4 μg/m^2^	70 obese or overweight	Blood pressure	Case–control	Retrospective nature, case–control despite claims about cross-sectional nature, sampling bias, recall bias, observation bias, confounding bias
Liivak and Tillmann (100)	6 patients with SW F/M: 4/2 Age: 6.8 (5.0-9.7)^*c*^	G + FC HCED: 20.7 (15.9-24.3) mg/m^2*c*^ FCD: 100 (50-175)^*c*^ μg	—	Blood pressure	Uncontrolled	Very small sample size, uncontrolled design, retrospective nature, sampling bias, recall bias, observation bias, confounding bias
Lim et al (101)	164 patients with C-CAH (SW: 76, SV: 88) F/M: 93/71 Age: F: 28 (23-36)^*b*^; M: 27 (23-33)^*b*^	G + FC HCED: 30 (20-30)^*b*^ mg FCD: NI	451	Body mass and composition, glucose homeostasis markers, lipids, blood pressure	Cross-sectional matched	Sampling bias, inability to establish causality, recall bias
Liu et al (102)	78 patients with NC F/M: 78/0 Age: 29.1 ± 4.2	G GD: HC: 5-20 mg^*e*^; DXM: 0.25-0.75 mg^*e*^	—	Body mass, glucose homeostasis markers, lipids	Retrospective chart analysis	Retrospective nature, sampling bias, observation bias, confounding bias
Maccabee-Ryaboy et al (103)	180 patients with C-CAH (SW: 120, SV: 60) F/M: 93/87 Age: 0-18^*e*^	G + FC HCED: 0-4 yrs old: born 1970-1994 22 ± 4 mg/m^2^, born 1995-2013 18 ± 6 mg/m^2^; 5-18 yrs old: born 1970-1994 16 ± 6 mg/m^2^; born 1995-2013 14 ± 5 mg/m^2^ FCD: 0-4 yrs old: 100 ± 30 μg; 5-18 yrs old: 80 ± 30 μg	—	Blood pressure	Retrospective chart analysis	Retrospective nature, sampling bias, observation bias, confounding bias
Marra et al (104)	20 patients with C-CAH (SW: 15, SV: 5) F/M: 10/10 Age: 13.6 ± 2.5	G + FC HCED: 15.0 ± 3.9 mg/m^2^ FCD: 54.8 ± 22.6 μg/m^2^	20 (healthy matched sex, for pubertal status and physical activity)	Body mass and composition, glucose homeostasis markers, Lipids, blood pressure, electrocardiogram, exercise test	Case–control	Small sample size, retrospective nature, case–control despite claims about cross-sectional nature, sampling bias, recall bias, observation bias, confounding bias
Metwalley et al (105)	32 patients with C-CAH (SW: 26, SV: 6) F/M: 18/14 Age: 13.6 ± 2.5	G + FC HCED: 13.5 ± 3.5 mg/m^2^ FCD: 72.6 ± 28.5 μg/m^2^	32 (matched for age, sex, pubertal status and socioeconomical status)	Body mass, blood pressure, endothelial function, intima–media thickness, systemic inflammation markers, echocardiogram	Case–control	Small sample size, retrospective nature, sampling bias, recall bias, observation bias, confounding bias
Metwalley et al (106)	36 patients with C-CAH (SW: 30, SV: 6) F/M: 26/10 Age: 9.7 ± 2.2	G + FC HCED: 14.6 ± 5.3 mg/m^2^ FCD: 74.3 ± 29.4 μg/m^2^	36 (matched for age, sex, pubertal status and socioeconomical status)	Body mass, glucose homeostasis markers, lipids, homocysteine, intima–media thickness echocardiogram	Case–control	Small sample size, retrospective nature, sampling bias, recall bias, observation bias, confounding bias
Metwalley et al (107)	36 patients with C-CAH (SW: 30, SV: 6) F/M: 25/11 Age: 13.7 ± 2.4	G + FC GD: NI FCD: 50-100 μg/m^2*e*^	36 (matched for age, sex, pubertal status and socioeconomical status)	Body mass and composition, glucose homeostasis markers, blood pressure, epicardial fat assessment, intima–media thickness, echocardiogram	Case–control	Small sample size, retrospective nature, sampling bias, recall bias, observation bias, confounding bias
Minette et al (108)	9 patients with C-CAH F/M: 4/5 Age: 18.0 ± 3.0 days	G + FC HCED: ∼20 mg/m^2^ FCD: NI	6	Echocardiogram	Prospective case–control	Small sample size, sampling bias, recall bias, observation bias, confounding bias
Mnif et al (109, 110)	26 patients with CAH (SW: 10, SV: 8, NC: 8) F/M: 15/11 Age: 27.4 ± 8.2	G + FC GD: HC: C-CAH—17.3 ± 4.6 mg/m^2^, NC—16.0 ± 3.4 mg/m^2^; DXM: 0.25-0.75 mg^*e*^ FCD: NI	—	Glucose homeostasis markers, lipids, blood pressure, intima–media thickness	Uncontrolled	Small sample size, no control group, retrospective nature, sampling bias, recall bias, observation bias, confounding bias
Mooij et al (111)	26 patients with C-CAH (SW: 7, SV: 3, SW-SV:14) F/M: 14/10 Age: first year of life	G + FC GD: NI FCD: 100 (60-150)^*c*^—150 (62.5-187.5) μg^*c*^	—	Blood pressure	Uncontrolled	Small sample size, no control group, retrospective nature, sampling bias, recall bias, observation bias, confounding bias
Mooij et al (112)	27 patients with CAH (SW: 20; SV: 6, NC: 1) F/M: 15/12 Age: 32.5 ± 11.7	G + FC HCED: 12.6 ± 4.5 mg/m^2^ FCD: 110 ± 60 μg	27 (age-, sex-, and BMI-matched healthy)	Glucose homeostasis markers, lipids, blood pressure, systemic inflammation markers, leptin, adiponectin, heart rhythm, coagulation/fibrinolysis markers	Case–control	Small sample size, retrospective nature, sampling bias, recall bias, observation bias, confounding bias
Mooij et al (113)	27 patients with CAH (SW: 24; SV: 2, NC: 1) F/M: 10/17 Age: 11.7 (8.8-16.0)^*d*^	G + FC HCED: 12.2 (11.2-13.2) mg/m^2*a*^ FCD: 98.5 (75.8-121.1) μg/m^2*a*^	—	Body mass and composition, glucose homeostasis markers, Lipids, blood pressure, intima–media thickness	Uncontrolled	Small sample size, no control group, retrospective nature, sampling bias, recall bias, observation bias, confounding bias
Mooij et al (114)	27 patients with CAH (SW: 24, SV: 2, NC: 1) F/M: 10/17 Age: 12.2 ± 2.3	G + FC HCED: 12.2 ± 2.6 mg/m^2^ FCD: 98.5 ± 53.6 μg/m^2^	27 (age-, and sex-matched healthy)	Body mass, electrocardiogram	Case–control	Small sample size, retrospective nature, sampling bias, recall bias, observation bias, confounding bias
Moreira et al (115)	68 patients with C-CAH (SW: 34, SV: 34) F/M: 48/20 Age: 28.4 ± 8.6	G + FC HCED: F: 11.2 ± 4.5 mg; M: 10.5 ± 3.7 mg (all DXM) FCD: 50 ± 25 μg	—	Body mass and composition, glucose homeostasis markers, Lipids, blood pressure	Uncontrolled	No control group, retrospective nature, sampling bias, recall bias, observation bias, confounding bias
Moreira et al (116)	33 patients with C-CAH (SW: 20, SV: 13) F/M: 20/13 Age: 11.9 ± 3.6	G + FC HCED: SW: 11.0 ± 2.5 mg/m^2^; SV: 11.6 ± 4.4 mg/m^2^ FCD: 50 ± 25 μg	33 (age-, sex-, and BMI-matched)	Body mass and composition, glucose homeostasis markers, Lipids, blood pressure	Case–control	Small sample size, retrospective nature, sampling bias, recall bias, observation bias, confounding bias
Navardauskaite et al (117)	32 patients with C-CAH (SW: 20, SV: 12) F/M: 19/13 Age: 26.9 (17.9-31.6)^*b*^	G + FC HCED: 15.4 (13.2-17.7) mg/m^2*b*^ FCD: 100 (50-125) μg^*b*^	32 (age-, BMI-, and ethnicity-matched)	Body mass and composition, glucose homeostasis markers, Lipids, blood pressure	Case–control	Small sample size, retrospective nature, sampling bias, recall bias, observation bias, confounding bias
Nebesio and Eugster (118)	91 patients with CAH F/M: 49/42 Age: subjects with CAH and hypertension: 13.0 ± 3.5; normotensive subjects with CAH: NI	G + FC HCED: subjects with CAH and hypertension: 16.4 ± 1.6 mg/m^2^; normotensive subjects with CAH: NI FCD: subjects with CAH and hypertension: 90 ± 50 μg; normotensive subjects with CAH: NI	—	Blood pressure	Uncontrolled	Small sample size, no control group, retrospective nature, sampling bias, recall bias, observation bias, confounding bias
Nermoen et al (119)	64 patients with C-CAH (SW: 33, SV: 31) F/M: 41/23 Age: 38.5 (19-72)	G + FC HCED: M: 34.9 ± 13.7; F: 28.0 ± 14.0 mg FCD: NI	36	Body mass and composition, blood pressure, 25-hydroxyvitamin D	Cross-sectional	Sampling bias, inability to establish causality, recall bias
Neumann et al (25)	331 patients with SW F/M: 186/145 Age: 0 month-36 months	G + FC HCED: between 23.6 ± 12.8 mg/m^2^ (month 0) and 11.1 ± 3.0 mg/m^2^ (month 30) FCD: between 138.3 ± 60.4 μg (month 3) and 85.8 ± 40.9 μg (month 36)	—	Blood pressure	Cohort	Retrospective nature, confounding bias, recall bias, losses to follow up
Özdemir et al (120)	25 patients with CAH F/M: 16/9 Age: 9.4 (1.5-16.8)^*d*^	G + FC HCED: 18.8 (9.2-23.9) mg/m^2*d*^ FCD: 100 μg	25 (age-, sex-, and body size-matched healthy)	Body mass, glucose homeostasis markers, arterial stiffness lipids, blood pressure, intima–media thickness, echocardiogram	Case–control	Small sample size, retrospective nature, case–control design despite claims about cross-sectional design, sampling bias, recall bias, observation bias, confounding bias
Paizoni et al (121)	90 patients with C-CAH (SW: 61, SV: 29) F/M: 51/39 Age: 29 (18-62)^*d*^	G + FC HCED: M: 16.2 (12.6-19.3) mg/m^2*b*^; F: 14.3 (9.6-16.7) mg/m^2*b*^ FCD: M: 45 (27-62) μg/m^2*b*^; F: 47 (30-60) μg/m^2*b*^	73 (age-, sex-, BMI-, and smoking-matched healthy)	Body mass and composition, lipids, blood pressure, intima–media thickness	Case–control	Retrospective nature, case–control design despite claims about cross-sectional design, sampling bias, recall bias, observation bias, confounding bias
Pall et al (122)	23 women with NC F/M: 23/0 Age: 22 ± 8	NT	27 healthy F, 54 obese PCOS, 52 lean PCOS	Glucose homeostasis markers	Prospective controlled	Small sample size, sampling bias, recall bias, observation bias, confounding bias
Paula et al (123)	7 patients with CAH (C-CAH: 4, NC: 3) F/M: 7/0 Age: 28 ± 3	NI	9	Glucose homeostasis markers	Prospective controlled	Very small sample size, sampling bias, recall bias, observation bias, confounding bias
Poyrazoglu et al (124)	11 (all C-CAH) F/M: 5/6 Age: 1.6 ± 2.7	G + FC No treatment before the study, then HCED: 14.6 ± 4.0 mg/m^2^ for 3 months FCD: NT before the study, then 40 ± 10 μg for 3 months in 5 patients	25	Body mass, leptin	Prospective controlled	Very small sample size, sampling bias, recall bias, observation bias, confounding bias
Raizada et al (125)	15 patients with C-CAH (SW: 2, SV:13) F/M: 15/0 Age: 27.5 ± 6.2	NI	12	Body mass	Case–control	Small sample size, retrospective nature, case–control design despite claims about cross-sectional design, sampling bias, recall bias, observation bias, confounding bias
Riepe et al (126)	6 patients with C-CAH F/M: NI Age: 19.0 ± 4.4	G + FC GD: NI FCD: NI	6 (age-, and sex—matched healthy)	Leptin, heart rhythm, exercise test	Case–control	Very small sample size, retrospective nature, sampling bias, recall bias, observation bias, confounding bias
Roche et al (127)	38 patients with SW F/M: 23/15 Age: 11.2 (6.1-18.2)^*c*^	G + FC HCED: 17.5 ± 2.1 mg/m^2^ FCD: 120 ± 24 μg/m^2^	—	Blood pressure	Cross-sectional	Sampling bias, inability to establish causality, recall bias
Rodrigues et al (128)	40 patients with C-CAH (SW: 29, SV: 11) F/M: 32/8 Age: 14.3 ± 4.4	G + FC HCED: 14.6 ± 3.6 mg/m^2^ FCD: average dose: 100 μg	73 (age-, sex-, and pubertal status-matched, matched healthy)	Body mass and composition, glucose homeostasis markers, Lipids, blood pressure, intima–media thickness	Case–control	Small sample size, retrospective nature, case–control design despite claims about cross-sectional nature, sampling bias, recall bias, observation bias, confounding bias
Rosenbaum et al (129)	84 patients with CAH (SW: 42, SV: 16, NC:26) F/M: 58/26 Age: 30.0 ± 8.8	G + FC HCED: 24 ± 10 mg FCD: 92 ± 37 μg	85 age-, sex-, and smoking-matched healthy)	Glucose homeostasis markers, lipids, blood pressure, intima–media thickness, adiponectin	Case–control	Small sample size, retrospective nature, sampling bias, recall bias, observation bias, confounding bias
Sartorato et al (130)	19 patients with C-CAH (SW: 12, SV: 7) F/M: 10/9 Age: 28.0 ± 3.5	G + FC GD: HC: 25 ± 2 mg; DXM: 0.4 ± 0.1 mg FCD: 120 ± 20 μg	19 (healthy matched for age and anthropometric parameters)	Glucose homeostasis markers, lipids, blood pressure, intima–media thickness	Case–control	Small sample size, retrospective nature, sampling bias, recall bias, observation bias, confounding bias
Saygili et al (131)	18 patients with NC F/M: 18/0 Age: 25.7 ± 8.9	NT	26 (age-, and weight-matched women)	Glucose homeostasis markers, leptin	Case–control	Small sample size, retrospective nature, sampling bias, recall bias, observation bias, confounding bias
Schröder et al (132)	39 patients with C-CAH (SW: 36, SV: 3) F/M: 17/22 Age: 12 (14-19)^*d*^	G + FC HCED: 11.7 (7.4-17.8) mg/m^2*d*^ FCD: NI	—	Blood pressure	Crossover study	Small sample size, short follow-up, possibility of “carry over” treatment effect
Seraphim et al (133)	60 patients with C-CAH (SW: 30, SV: 30) F/M: 41/19 Age: onset of DXM treatment: 20.5 ± 9.8; last assessment: 31.9 ± 9.6	G + FC DXM: 0.18 ± 0.07 mg/m^2^ FCD: 55.7 ± 10.6 μg	—	Body mass, glucose homeostasis markers	Retrospective cohort	Small sample size, retrospective nature, confounding bias, recall bias, losses to follow up
Speiser et al (134)	6 women with NC F/M: 6/0 Age: 27.0 ± 9.8	NT	12 (women of similar age and with similar weight)	Body mass, glucose homeostasis markers	Case–control	Very small sample size, retrospective nature, sampling bias, recall bias, observation bias, confounding bias
Stikkelbroeck et al (135)	30 patients with CAH (SW: 24, SV: 3 NC: 3) F/M: 15/15 Age: M: 21.7 ± 2.4; F: 20.6 ± 2.0	G (no information about FC) GD: NI	30 (age-, and sex—matched healthy)	Body mass and composition	Case–control	Small sample size, retrospective nature, sampling bias, recall bias, observation bias, confounding bias
Subbarayan et al (136)	107 patients with C-CAH (SW: 79, SV: 28) F/M: 68/39 Age: 9.2 (0.4-20.5)^*d*^	G + FC HCED: 13.3 ± 4.4 mg/m^2^ FCD: 102 ± 50 μg/m^2^	—	Body mass, glucose homeostasis markers, lipids, blood pressure	Retrospective cross-sectional	Retrospective nature, sampling bias, inability to establish causality, recall bias
Tony Nengom et al (137)	19 patients with CAH (SW: 4, 11OHD: 15) F/M: 14/5 Age: 6.3 ± 3.8	NI	38 (age-, and sex-matched healthy)	Blood pressure, echocardiogram	Case–control	Small sample size, sampling bias, recall bias, confounding bias
Torky et al (138)	57 patients with C-CAH (SW: 39; SV: 18) F/M: 22/35 Age: first assessment: 5.4 (3.0-7.9)^*b*^; most recent: 23.3 (20.8-27.7)^*b*^	NI	**—**	Body mass, glucose homeostasis markers, lipids, blood pressure	Retrospective longitudinal study	Retrospective nature, sampling bias, recall bias, confounding bias
Tuhan et al (139)	44 patients with C-CAH (SW: 30, SV: 14) F/M: 22/22 Age: 10.3 ± 4.3	G (NI about FC) HCED: 13.4 ± 4.0 mg/m^2^	39 (age-, sex-, and pubertal status-matched healthy)	Body mass and composition, blood pressure, heart rhythm, echocardiogram	Prospective, controlled study	Small sample size, sampling bias, recall bias, confounding bias
Tuhan et al (140)	34 patients with C-CAH (SW: 22, SV: 12) F/M: 17/17 Age: 10.4 ± 3.9	G + FC HCED: 12.8 ± 4.1 mg/m^2^ FCD: 100 μg	31	Body mass, glucose homeostasis markers, lipids, blood pressure, intima–media thickness	Prospective controlled study	Small sample size, sampling bias, recall bias, confounding bias
Ubertini et al (141)	20 patients with C-CAH (SW: 15, SV: 5) F/M: 14/6 Age: 13.38 ± 4.11	G + FC HCED: 14.16 ± 5.46 mg/m^2^ FCD: 60 ± 20 μg	—	Blood pressure, endurance exercise, echocardiogram	Uncontrolled prospective study	Very small sample size, sampling bias, confounding bias
Virayan et al (142)	52 patients with C-CAH (SW: 35, SV: 17) F/M: 38/14 Age: 12 (3-21)^*d*^	G + FC HCED: 11.9 (5.1-17.7) mg/m^2*d*^ FCD: 50 (25-150) μg^*d*^	58 (age-matched healthy)	Body mass, glucose homeostasis markers, lipids, blood pressure	Case control	Retrospective nature, sampling bias, recall bias, observation bias, confounding bias
Völkl et al (143)	55 patients with C-CAH (SW: 45, SV: 10) F/M: 32/23 Age: 12.34 ± 4.06	G + FC HCED: 15.30 ± 4.83 mg/m^2^ FCD: 48.0 ± 21.2 μg/m^2^	—	Body mass, fat distribution, blood pressure	Cross-sectional	Sampling bias, inability to establish causality, recall bias
Völkl et al (144)	89 patients with C-CAH (SW: 78, SV: 11) F/M: 48/41 Age: 8.9 ± 4.7	G + FC HCED: 14.7 ± 4.8 mg/m^2^ FCD: 63.1 ± 38.8 μg/m^2^	—	Body mass, leptin	Cross-sectional	Sampling bias, inability to establish causality, recall bias
Völkl et al (145, 146)	51 patients with C-CAH (SW: 42, SV: 9) F/M: 30/21 Age: 12.0 ± 3.8	G + FC HCED: 16.0 ± 4.9 mg/m^2^ FCD: 46 ± 21 μg/m^2^	51 (matched for age, sex, pubertal development and BMI)	Body mass, leptin, adiponectin	Cross-sectional matched-pairs study	Sampling bias, inability to establish causality, recall bias
Wasniewska et al (147)	18 patients with CAH (C-CAH: 9, NC: 9) F/M: 8/10 Age: 16.2 ± 2.2	G + FC HCED: 17.1 ± 2.9 mg/m^2^ FCD: 100 ± 100 μg	16 (age-matched healthy)	Body mass and composition, glucose homeostasis markers, Lipids, blood pressure, intima–media thickness	Case control	Small sample size, retrospective nature, sampling bias, recall bias, observation bias, no BMI-matched despite claims, other confounding bias
Weise et al (148, 149)	9 (patients with C-CAH F/M: 4/5 Age: 15.0 ± 2.4	G + FC GD: HC: 14.8 ± 2.1 mg/m^2^; DXM: 0.35 mg FCD: 117 ± 39 μg	9 (age-, sex-, and percent body fat-matched healthy)	Exercise test, heart rhythm	Cohort/randomized, double blind, crossover	Very small sample size, sampling bias, recall bias, observation bias, confounding bias
Wierzbicka-Chmiel et al (150)	19 patients with SW F/M: 7/12 Age: 23.7 ± 3.8	G + FC HCED: 20.0 ± 6.0 mg/m^2^ FCD: 29 (26-58) μg/m^2*b*^	20 (age-, BMI-, and ethnicity-matched)	Glucose homeostasis markers, lipids, blood pressure, endothelial function, intima–media thickness	Case–control	Small sample size, retrospective nature, sampling bias, recall bias, observation bias, confounding bias
Williams et al (151)	37 patients with CAH (C-CAH: 25, NC: 12) F/M: 20/17 Age: C-CAH: 7.5 (5.9-9.1)^*a*^; NC: 12.0 (9.5-14.4)^*a*^	G + FC HCED: C-CAH: 13.9 (13.1-14.7) mg/m^2*a*^; NC: 8.2 (7.5-9.4) mg/m^2*a*^ FCD: C-CAH: 82 (69-95) μg/m^2*a*^; NC: 18 (0-38) μg/m^2*a*^	41	Body mass and composition, glucose homeostasis markers, lipids	Case–control	Small sample size, retrospective nature, between-group differences in age (youngest with C-CAH), sampling bias, recall bias, observation bias, other, confounding bias
Yoon and Cheon (152)	33 patients with C-CAH (SW: 30, SV: 3) F/M: 17/16 Age: 7.4 (0.1-23.8)^*d*^	G (NI about FC) HCED: current: 14.3 ± 5.7 mg/m^2^; average: 21.2 ± 7.2 mg/m^2^	—	Body mass, glucose homeostasis markers, lipids	Cross-sectional	Sampling bias, inability to establish causality, recall bias
Zhang et al (153)	30 patients with SV F/M: 30/0 Age: 21.0 ± 4.3	NT	30 (age-matched)	Body mass, glucose homeostasis markers, lipids, systemic inflammation markers, adiponectin	Case–control	Small sample size, retrospective nature, between-group differences in age (youngest with C-CAH), sampling bias, recall bias, observation bias, other, confounding bias
Zimmermann et al (154)	27 patients with C-CAH (SW: 12, SV: 15) F/M: 20/7 Age: 12.9 ± 7.6	G + FC HCED: SW: 21.5 ± 5.0 mg/m^2^; SV: 16.2 ± 4.7 mg/m^2^ FCD: 50-100μg^*e*^	27 (age-, sex-, and BMI-matched healthy)	Glucose homeostasis markers, lipids	Case–control	Small sample size, retrospective nature, between-group differences in age (youngest with C-CAH), sampling bias, recall bias, observation bias, other, confounding bias

The results are presented as means ± SD (unless otherwise stated).

Age is expressed in years (unlike otherwise stated).

Glucocorticoid dosage is expressed in hydrocortisone equivalent doses (unlike otherwise stated).

Abbreviations: 11OHD, 11β-hydroxylase deficiency; 17OHD, 17α-hydroxylase deficiency; 21OHD, 21-hydroxylase deficiency; 3HSD, 3β-hydroxysteroid dehydrogenase deficiency; BMI, body mass index; CA, cortisone acetate; CAH, congenital adrenal hyperplasia; C-CAH, classic congenital adrenal hyperplasia; DXM, dexamethasone; F, females; FC, fludrocortisone; FCD, daily fludrocortisone dose; G, glucocorticoids; GD, daily glucocorticoid dose; HC, hydrocortisone; HCED, daily hydrocortisone equivalent dose; LAH, lipoid adrenal hyperplasia; M, males; NC, nonclassic phenotype; NI, no information; NT, no treatment; PCOS, polycystic ovary syndrome; PD, prednisone; PDL, prednisolone; POR, cytochrome P450 oxidoreductase deficiency; SG, synthetic glucocorticoids; SV, simple virilizing phenotype; SW, salt-wasting phenotype; TML, triamcinolone.

^*a*^Mean (95% coincidence intervals).

^*b*^Median (25th-75th percentile).

^*c*^Mean (range).

^*d*^Median (range).

^*e*^Range.

## Mortality and Cardiometabolic Morbidity Associated With CAH

Before the implementation of newborn screening programs in many countries worldwide, undiagnosed and untreated SW-CAH resulted in in a high rate of morbidity and mortality mostly caused by severe adrenal crisis due to acute glucocorticoid, mineralocorticoid, and epinephrine deficiencies which led to hyponatremia, hyperkalemia, hypoglycemia, and dehydration ultimately culminating in hypovolemic shock if not promptly recognized and adequately treated. Nowadays, mortality in the early childhood is reported very rarely as education of parents and health care providers as well as availability of medication improved (155-158).

A more controversial question is the prevalence of morbidity and mortality at later stages of life. In a cohort study conducted in the UK, all-cause mortality was increased between the first and fourth year of life but not in older subjects; however, most deaths were in girls of Indian subcontinent origin (159). Furthermore, it should be noted that the study included only few individuals older than 35 years of age. A UK register–based study identified 270 patients with CAH and found an increased mortality in both females and males with CAH, but detailed cause of death was not available (87). Analysis of 588 patients with CAH from the Swedish national CAH registry showed increased lifetime mortality in both sexes. After the first year of life, the mortality rate was increased in females and, when clinical severity of CAH was analyzed, only in patients with unclear phenotype (160). Cardiovascular complications were after adrenal crisis the most frequent cause of death (32%) and in two-thirds of cases resulted from cerebrovascular cause. However, cardiovascular mortality might have been overestimated by coexisting severe infection with potentially undiagnosed adrenal crisis (160).

An increased total cardiometabolic risk was observed in another study of the same cohort, with hypertension, hyperlipidemia, atrial fibrillation, venous thromboembolism, obesity, diabetes (mainly type 2), and obstructive sleep disorder being increased compared with controls matched for sex, year, and place of birth (74). The increase in cardiovascular disease was mainly seen in females and the number of individual cardiovascular diseases increased with age compared with matched controls. Acute coronary syndrome was more prevalent in males with SW-CAH, venous thromboembolism was reported more frequently in females with either SW-CAH or NC-CAH, while the risk of stroke was elevated in females with NC-CAH (74).

A pan-European DSD study with 226 adults with CAH (almost all females) included found that cardiovascular disease was increased compared with the general population (75). Unfortunately, the different cardiovascular diseases were not separately analyzed.

A registry-based study from South Korea demonstrated that patients with CAH had a higher risk of cardiovascular disease and stroke than matched controls (161). Moreover, the presence of CAH was associated with higher risk for diabetes mellitus, dyslipidemia, and hypertension. In patients with CAH aged less than 40 years, the risk of diabetes mellitus, dyslipidemia, and hypertension was increased but disappeared in older individuals with CAH compared with controls. Thromboembolism, defined as thrombosis of the lower limb veins, pulmonary embolism, and embolism, or thrombosis of other deep veins (except for portal vein thrombosis), was more prevalent only in women older than 40 years. Although the number of individuals using oral contraceptives was not provided, the increased risk of thromboembolism probably was not associated with taking these agents, because they are much less frequently used in South Korea than in the United States and Europe, and are generally not recommended in this age group (162). The mortality was also increased in patients with CAH but the cause of death was not reported (161).

Lastly, in a recent 10-year retrospective cross-sectional analysis including 254 adult patients with 21OHD, aged 18 to 70 years, the prevalence of cardiovascular diseases was estimated at 7.5%, and the most commonly observed disorder was stroke (59). Interestingly, the risk of cardiovascular morbidity in this American study was exactly the same as in the Swedish register-based study (74) and almost the same as in the South Korean register-based study (7.4%) (161). The prevalence was greatest (25%) in patients older than 60 years, and in the adjusted model cardiovascular morbidity was significantly associated with hypertension, glucocorticoid dose and plasma renin activity. There were no differences in the prevalence of cardiovascular disorders between C-CAH and NC-CAH, as well as between males and females (59).

In summary, the few studies investigating definitive and clinically meaningful outcomes, such as death and morbidity, showed that patients with CAH had increased prevalence of cardiometabolic complications, which may have contributed to the increased mortality. However, many older individuals in the included studies were born before the introduction of neonatal screening for CAH resulting in delayed diagnosis.

## Body Mass and Composition

Most studies conducted so far have shown increased body mass index (BMI) in individuals with CAH compared with controls (Table 2). The percentage of individuals with CAH and obesity ranged from 10.3% to 70%. Although excessive weight gain has been often reported in childhood, in some studies including exclusively or mainly younger patients, differences in comparison with controls have not reached significance levels (Table 2). Data from expert centers indicate that only 4% of adults with C-CAH received antiobesity medications or underwent bariatric surgery (163). Such a low percentage results from the fact that antiobesity pharmacotherapy is expensive and often not reimbursed, while access to metabolic surgery is still limited. Moreover, newer FDA/EMA-approved antiobesity medications (naltrexone/bupropion, liraglutide, phentermine/topiramate, semaglutide, setmelanotide, and tirzepatide) have not been studied yet in patients with CAH.

**Table 2.** Body mass and fat distribution in patients with congenital adrenal hyperplasia

Authors	Major findings	Conclusion (consequences for cardiometabolic health)^*a*^
Abdel Meguid et al (38)	Obesity/overweight in 60% of children with C-CAH; hydrocortisone-treated: obesity—12.5%, overweight—50%, prednisolone-treated: obesity—32%, overweight—27.3%	C-CAH in children was accompanied by increased body mass (I)
Ahmed et al (39)	No differences in BMI vs controls; no differences in BMI between patients treated with hydrocortisone and prednisone	No association between C-CAH and changes in body mass in children (N)
Akyürek et al (40)	BMI higher vs controls; no differences in waist to hip ratio	C-CAH in children was accompanied by increased body mass but no changes in fat distribution (I)
Amr et al (41)	BMI SDS higher vs controls; no correlation of BMI SDS with free testosterone, glucocorticoid dose and treatment duration; no difference in weight SDS and BMI SDS between SW-CAH and SV-CAH	C-CAH in children was accompanied by increased body mass (I)
Apsan et al (43)	No differences in BMI *z*-score between children receiving hydrocortisone twice and 3 times daily	No association between frequency of hydrocortisone administration and body weight in children (X)
Ariyawatkul et al (44)	Obesity in 33%, overweight in 14% of patients; BMI and BMI SDS insignificantly higher, waist to hip and waist to height ratio higher vs controls; no differences in weight; no differences between obese and nonobese patients in age, pubertal stage, glucocorticoid type, hydrocortisone equivalent dose, and family history of metabolic syndrome	C-CAH in children and young adults was accompanied by increased risk of obesity and overweight, and increased visceral fat distribution (I)
Arlt et al (3)	BMI in both males and females with CAH higher than that in a national health survey; BMI higher in females with C-CAH than with NC; obesity in 41% and overweight in 37% of affected patients; after adjusting for age distribution: obesity in 37.1% of men with C-CAH, 52.2% of women with C-CAH and 34.9% of women with NC (more frequently than in the health survey data)	Increased body weight and high prevalence of obesity in adult population with CAH (I)
Bachelot et al (46)	No differences in BMI between patients with SW, SV, and NC, no association between BMI and hydrocortisone dose, duration of treatment, and 17OHP levels	Type of CAH did not determine body mass in young adults (X)
Bachelot et al (47)	BMI >25 kg/m^2^ in 44% of the cohort (53% if women, 40% of men); 17OHP, androstenedione, and renin higher in subjects ≤25 kg/m^2^ than >25 kg/m^2^, no difference in progesterone and ACTH; determinants of BMI >25 kg/m^2^: age and androstenedione	High prevalence of obesity/overweight in adult population (I)
Bacila et al (48)	Weight SDS, BMI SDS, waist circumference SDS, and hip circumference SDS higher vs control subjects; in subgroup analyses significant changes for: BMI SDS in girls and in patients aged 12-18 years, waist circumference SDS in girls and >12 years old; hip circumference: men aged 8-12 years; obesity in 27% and 22% of patients with CAH and 10.8% and 10.8% of controls; higher weight SDS and BMI SDS in patients with hydrocortisone equivalent dose <10 mg/m^2^ compared with the rest of the cohort; no correlation between anthropometric measures and hydrocortisone equivalent dose	C-CAH in children was accompanied by increased body mass (I)
Ben Simon et al (50)	BMI *z*-score, % fat, % truncal fat higher, muscle to fat ratio *z*-score lower vs controls	NC-CAH in children was accompanied by increased body mass and fat content (I)
Bonfig et al (51)	BMI SDS higher in SW than SV; correlation between BMI SDS and blood pressure	Body mass higher in children with SW-CAH than SV-CAH (X)
Borges et al (52)	BMI and % fat higher vs control subjects in males; insignificantly higher % fat in females	C-CAH in young adults was accompanied by increased body mass and increased fat content (I)
Borges et al (53)	BMI and % fat higher vs control subjects in males but not in women; no differences in % fat between affected and control men and women	C-CAH in young adults was accompanied by increased body mass and increased fat content (I)
Borges et al (54)	BMI and % fat higher vs control subjects in men; no differences in weight and BMI, and insignificantly higher % fat vs control subjects in women	C-CAH in young adults was accompanied by increased body mass and increased fat content (I)
Bouvattier et al (56)	Obesity in 30% and overweight in 22% of patients; higher BMI in dexamethasone-treated than hydrocortisone-treated subjects with CAH	High prevalence of obesity and overweight in patients with C-CAH (I)
Cameron et al (57)	No differences in BMI in both sexes vs control subjects; fat/lean ratio higher in affected men and normal in affected women	Increased fat content in boys and young adult males with C-CAH, despite unaltered body mass (I)
Charoensri and Auchus (59)	Obesity in 42.1% of patients; more frequent in C-CAH (54.4% of males and 46.7% of women) than in NC-CAH (18.2% of males and 33.3% of females); more frequent in late adulthood (65.6%) than in young adults (30%)	High prevalence of obesity in adults with CAH (particularly C-CAH) (I)
Christiansen et al (60)	Fat mass percentage in affected men and in all population of patients with CAH higher vs control subjects; no differences in fat mass percentage in women	Increased fat content in men with C-CAH (I)
de Oliveira et al (62)	No difference in BMI vs controls; % fat mass greater vs gender-matched controls; % fat mass android in women and % fat mass gynoid in M greater vs respective controls; bioimpedance parameters greater vs respective controls only in women	Increased fat content in young adults with C-CAH (I)
de Vries et al (64)	Body fat mass insignificantly higher in treated patients vs off-treatment; no difference in BMI, waist circumference, hip circumference, and waist to hip ratio	Active glucocorticoid treatment may increase fat content in children with NC-CAH (X)
Debor et al (65)	BMI SDS above 0 in both boys and girls with androstenedione levels under the lower detection limit; an increase in BMI SDS during the observation period	C-CAH may predispose children to higher weight gain (I)
Delai et al (66)	Waist to hip ratio greater vs sex- and BMI-matched control subjects; no differences in waist circumference, fat, and lean mass	NC-CAH may be associated with visceral fat distribution (I)
Espinosa-Reyes et al (69)	No differences in waist circumference and epicardial fat thickness vs sex-, age- and BMI-matched healthy subjects	Difficult to interpret because of BMI-matching (X)
Falhammar et al (70)	<30 years old: no difference in body weight, BMI, waist circumference, and waist to hip ratio vs controls; ≥ 30 years old: BMI, waist to hip ratio, and lean mass higher vs controls; weight, BMI, waist circumference, waist to hip ratio, and lean mass higher in CAH subjects ≥30 than <30 years old; similar percentage of body fat and total and regional fat mass vs controls in both age groups	CAH was associated with increased body mass and visceral tissue distribution in patients aged 30 or older (I)
Falhammar et al (72)	Obesity in 23% of men with CAH (controls 9%, which was not significant lower); ≥ 30 years old: BMI, waist to hip ratio, total fat mass, trunk fat mass, and total fat/total lean mass ratio higher vs controls; <30 years old: trunk/total fat mass ratio insignificantly lower vs Controls	CAH was associated with increased body mass, increased fat content and visceral tissue distribution in patients aged 30 or older (I)
Falhammar et al (74)	Over 10-fold increase in prevalence of obesity, observed both in men and females with CAH; obesity more prevalent in all subgroups of patients, except in I2G males and P30L females. Obesity most pronounced in males and females with NC	High prevalence of obesity in patients with CAH (I)
Falhammar et al (75)	Increased BMI more prevalent vs controls; obesity/overweight in >50% of subjects with CAH	CAH was associated with increased body mass and predisposes to obesity/overweight (I)
Farghaly et al (76)	BMI SDS higher vs controls; correlation of BMI SDS with neopterin levels	C-CAH was associated with increased body mass in children (I)
Finkielstain et al (77)	Obesity in 35% of children and in ∼1/3 of adults; no difference in frequency of obesity between patients with C-CAH and NC; obese children more frequently than nonobese on long-acting glucocorticoids; insulin, HOMA-IR, 17OHP, and androstenedione higher in obese vs nonobese children with CAH; no difference between obese and nonobese children in glucocorticoid dose, family history of obesity, sex or age; glucocorticoid dose higher in obese vs nonobese adults with CAH; leptin levels higher in obese than in nonobese adults with CAH; no differences between obese and nonobese adult subjects with CAH in CAH type, glucocorticoid type, androgen levels, age, sex, and family history of obesity	High prevalence of obesity in children and adults with both C-CAH and NC-CAH (I)
Hagenfeldt et al (81)	BMI, body weight, and body fat higher vs controls	C-CAH in young adult females was associated with increased body mass (I)
Han et al (82)	No differences in BMI and waist circumference between patients with CAH receiving hydrocortisone, prednisolone, hydrocortisone, and prednisolone or dexamethasone	Type of glucocorticoid did not determine body mass in CAH (X)
Harrington et al (83)	No differences in BMI z-score, waist to hip ratio vs healthy controls	C-CAH was not accompanied by changes in body weight in children (N)
Hashemi Dehkordi et al (84)	Positive correlation between BMI and 17OHP	Body weight in children may be increased if C-CAH was poorly controlled (I)
Janus et al (86)	Total body water % lower in subjects with SV, higher in case of Del/I2G and insignificantly higher in case of Del/Del; fat mass % highest in SV and lowest in Del/I2G subgroup; lean tissue % lowest in SV and highest in Del/I2G subgroup; no differences in BMI SDS between genotypes	Phenotype/genotype may determine fat mass in CAH (I)
Jenkins-Jones et al (87)	BMI in males and females higher vs controls	CAH was associated with increased body mass (I)
Kara et al (88)	Visceral adiposity index, %fat, and fat mass higher vs BMI-, age-, and gender-matched controls; waist circumference and fat mass lower in hydrocortisone- than dexamethasone-treated patients; correlations between visceral adiposity index and HOMA-IR, SBP, Framingham Risk Score, glucocorticoid dose, 17OHP, and androstenedione levels; correlation between dexamethasone use and waist circumference, %fat, and fat mass	C-CAH was associated with increased fat content and visceral fat distribution (I)
Kępczyńska-Nyk et al (89)	No difference in BMI vs healthy females and women with PCOS; waist circumference increased vs healthy females but not vs women with PCOS; no difference in % fat vs women with PCOS.	C-CAH was associated with visceral fat distribution in women (I)
Kim et al (90, 91)	Comparisons of BMI, BMI z-score, waist circumference, and waist to hip ratio between CAH and controls impossible (both groups were BMI-matched); increased amounts of visceral and subcutaneous adipose tissue and increased ratio of visceral to subcutaneous fat amount in individuals with CAH vs controls; no differences between males and females in amount of visceral and subcutaneous adipose tissue; correlations between amounts of visceral and subcutaneous fat and BMI z-score, waist circumference, waist to hip ratio, trunk, and total fat mass, HOMA-IR, plasma lipids (except for HDL cholesterol), plasminogen activator inhibitor-1, and hsCRP; inverse correlations between amounts of visceral and subcutaneous adipose tissue and sex hormone–binding globulin; no correlations with 17OHP and glucocorticoid dose	C-CAH was associated with visceral fat distribution in children and young adults (I)
Kroese et al (94)	No changes in amounts of subcutaneous and visceral fat, the visceral to subcutaneous fat ratio, and % liver fat after treatment with pioglitazone; reduction in % liver fat only in subjects with high baseline % liver fat	Impaired adipose tissue expression of peroxisome proliferator-activated receptor-γ (X)
Lim et al (101)	Weight, waist circumference, and, in females, BMI higher vs controls; obesity risk 2-fold higher in females with CAH than control	C-CAH was associated with increased mass and visceral fat distribution in young adults, and predispose to obesity (I)
Liu et al (102)	Obesity in 10.3% of patients; overweight in 23.1% of patients	Obesity/overweight common in young women with NC-CAH (I)
Marra et al (104)	BMI SDS, waist to hip ratio, total body fat higher vs controls in males and females; waist and hip circumference higher vs controls only in men; no differences in total body lean mass between patients and control	C-CAH was associated with increased body mass, increased fat content and visceral fat distribution in children (I)
Metwalley et al (105)	BMI SDS higher vs controls	C-CAH was associated with increased body mass in children (I)
Metwalley et al (106)	BMI SDS higher vs controls; correlation between BMI SDS and homocysteine levels	C-CAH was associated with increased body mass in children (I)
Metwalley et al (107)	BMI SDS, waist and hip circumference, waist to hip ratio, and epicardial fat thickness higher vs controls; correlations between epicardial fat thickness and BMI, waist circumference, SBP, DBP, HOMA-IR, hsCRP, 17OHP, total testosterone, carotid intima–media thickness, left ventricular mass index, and mitral deceleration time	C-CAH was associated with increased body mass in children (I)
Mnif et al (109, 110)	Obesity in 30.7% of patients, overweight in 30.7% of patients; increased fat mass in 46.1% of patients; obesity or overweight in 60% of individuals with SW, 75% of individuals with SV, and 50% of individuals with NC-CAH	High prevalence of obesity and overweight in patients with CAH, irrespectively of its form (I)
Mooij et al (113)	BMI SDS higher vs reference population and correlating with 17OHP and androstenedione; obesity in 14.8%, overweight in 25.9% of individuals with CAH; waist and hip circumference and waist to hip ratio increased vs reference population	CAH was associated with increased body weight and visceral fat distribution, as well as predisposes to obesity in children (I)
Mooij et al (114)	BMI SDS insignificantly higher vs controls	CAH may be associated with increased body weight in children (I)
Moreira et al (115)	Obesity in 23.5% of patients with CAH, not correlated with glucocorticoid dose and treatment duration; higher BMI in adult men than women; no difference in proportion of individuals with increased waist circumference; higher BMI and waist circumference in *Bcl*I heterozygous than homozygous carriers; correlations of BMI with SBP, DBP, triglycerides, HOMA-IR and reverse with HDL cholesterol; no correlations of BMI with glucocorticoid dose, treatment duration, testosterone, and androstenedione	C-CAH may be associated with increased risk of obesity in adults (I)
Moreira et al (116)	Obesity in 30.3% of patients; no differences in BMI z-score and waist circumference between CAH girls and boys, and between SW and SV; correlation of BMI z-score with SBP, HOMA-IR, total cholesterol, LDL cholesterol, and triglycerides; no correlation of BMI z-score with sex, clinical form, glucocorticoid dose, and treatment duration	High prevalence of obesity in children with C-CAH (I)
Navardauskaite et al (117)	Obesity more often in patients (30%) with CAH vs controls (0%), no differences in overweight (37 vs 31%); BMI-SDS and waist to hip ratio higher in patients with CAH vs controls, and in males with SV vs men with SW; total fat mass higher in patients with CAH vs controls; total fat mass, visceral adipose tissue mass, and subcutaneous adipose tissue mass higher in women with SV vs women with SW and in men with SV vs men with SW; no correlation between BMI, waist to hip ratio, total fat mass, visceral adipose tissue mass, and subcutaneous adipose tissue mass, and glucocorticoid type and dose; BMI SDS and total fat mass in patients with Null/Null and Null/I2G genotypes lower vs remaining genotypes	C-CAH predisposes to obesity in young adults (I)
Nermoen et al (119)	Males: waist to hip ratio higher vs age-matched controls, no differences in BMI and waist circumference; females: no differences in BMI, waist circumference, and waist to hip ratio vs controls; % total fat higher vs reference values in both males and females; no difference in fat mass between SW and SV; positive correlations between fat mass and glucocorticoid dose; negative correlation between total fat mass and testosterone concentration in males	C-CAH is associated with increased fat content in adults, and in males additionally with viscera; fat distribution (I)
Özdemir et al (120)	No difference in BMI and BMI SDS vs controls; BMI independently correlated with carotid intima–media thickness and aortic stiffness index	CAH in children was not associated with changes in body mass (N)
Paizoni et al (121)	Obesity in 26.7% of patients; BMI higher vs general German population and increasing with age; fat mass/lean mass ratio higher vs controls in both men and women; increased waist to hip ratio in 17.9% of men (>102 cm) and 34.6% of women (>88 cm); % body fat increased only in men; % body fat lower in men vs women with CAH and in hydrocortisone vs treated with synthetic glucocorticoids; comparisons of BMI with controls impossible (both groups BMI-matched); independent correlations between BMI and waist to hip ratio and intima–media thickness; correlations between BMI and age and urine pregnanetriol; waist to hip ratio correlated with age and free testosterone	C-CAH in adults was associated with increased body mass and visceral fat distribution, and predisposes to obesity (I)
Poyrazoglu et al (124)	No difference vs control subjects both before and after treatment	C-CAH was not associated with changed body mass in small children (N)
Rodrigues et al (128)	BMI Z score and waist to hip ratio higher vs controls	C-CAH was accompanied by increased body mass and visceral fat distribution in children and young adults (I)
Seraphim et al (133)	No effect of dexamethasone treatment for 11.5 ± 4.9 years on BMI SD and obesity/overweight and metabolic syndrome; dexamethasone-induced increase in waist to head ratio and HOMA-IR (correlating with each other); no difference in dexamethasone dose between patients with CAH and obesity/overweight compared with normal weight	Transition from short acting glucocorticoid to dexamethasone may increase visceral fat distribution (X)
Speiser et al (134)	No differences in BMI vs controls	Unaltered body mass in women with NC-CAH (N)
Stikkelbroeck et al (135)	BMI in both males and females higher vs controls; fat mass in legs in both sexes higher vs controls; fat mass in trunk in both sexes and in arms in women higher vs controls after adjustment for height; lean mass in trunk and legs in males lower vs controls; no difference in body weight vs controls	Increased fat mass in adolescents and young adults with CAH (I)
Subbarayan et al (136)	Obesity in 23.6% of individuals with CAH (33% males, 17.8% females); BMI SDS and weight SDS higher compared with population and correlating with age; BMI SDS inversely correlating with fludrocortisone dose but not correlating with hydrocortisone dose	High prevalence of obesity in children and young adults with C-CAH (particularly males) (I)
Torky et al (138)	Obesity in 70.2% of patients; most frequently in subjects <2 or >30 years old; an increase in prevalence from preschool to school-aged children, a downward trend when going through puberty and into young adulthood; no independent association of obesity with sex, glucocorticoid dose, type or percent nighttime dose and adrenal biomarkers; the only risk factor of obesity in adults was maternal obesity during childhood	High prevalence of obesity in patients with C-CAH, particularly in small children and adults (I)
Tuhan et al (139)	BMI, BMI SDS, and weight SDS higher while weight insignificantly higher vs controls	C-CAH may predispose children to increased body weight (I)
Tuhan et al (140)	BMI SDS higher vs controls both in males and females; no differences in weight SDS; BMI SDS higher in uncontrolled than controlled CAH	No conclusions concerning body mass can be drawn because of inconsistent findings (X)
Virayan et al (142)	Obesity in 27%, overweight in 33% of patients with CAH; BMI SDS and weight SDS higher vs controls; correlations between BMI SDS and SBP, DBP, and HOMA-IR; no correlations with glucocorticoid and fludrocortisone doses	High prevalence of obesity and overweight in children and young adults with C-CAH (I)
Völkl et al (143)	BMI SDS and subscapular skinfold thickness in males and females higher than expected for normal population; no difference in BMI between SW and SV; correlation of BMI and subscapular skinfold thickness with blood pressure	High body weight and high fat content in children and young adults with C-CAH (I)
Wasniewska et al (147)	Insignificantly higher BMI SDS and waist to hip ratio between patients with C-CAH than in controls; no differences between NC and controls and between C-CAH and NC	CAH may predispose adolescents and young adults to increased body weight (I)
Williams et al (151)	No differences in BMI SDS between C-CAH, NC, and controls; weight SDS insignificantly higher in subjects with NC than in subjects with C-CAH and controls; fat mass increased in C-CAH; fat-free mass increased in NC	C-CAH was associated with increased fat content in children (I)
Yoon and Cheon (152)	Overweight/obesity in 55.6% of patients >2 years old	High prevalence of obesity and overweight in children and young adults with C-CAH (I)
Zhang et al (153)	BMI higher vs controls; no differences in weight	Because of inconsistent results no conclusions can be drawn concerning untreated SV (X)
Zimmermann et al (154)	No difference in BMI and BMI Z score between SW and SV; comparisons with controls impossible (both groups BMI-matched)	Type of C-CAH did not determine body mass in children and young adults (X)

^*a*^Consequences for cardiometabolic health: I, increased cardiometabolic risk; N, no impact on cardiometabolic risk; D, decreased cardiometabolic risk; X, no conclusions concerning cardiometabolic risk can be drawn based on these findings.

Summary: Unfavorable impact of CAH on body mass and fat distribution in 55 studies (77.5%), no impact of CAH in 5 studies (7.0%); favorable impact of CAH 0 study (0%), while 11 studies (15.5%) inconclusive.

Abbreviations: 17OHP, 17-hydroxyprogesterone; ACTH, adrenocorticotropic hormone; BMI, body mass index; CAH, congenital adrenal hyperplasia; C-CAH, classic congenital adrenal hyperplasia; DBP, diastolic blood pressure; HDL, high-density lipoprotein; HOMA-IR, homeostatic model assessment for insulin resistance index; hsCRP, high-sensitivity C-reactive protein; LDL, low-density lipoprotein; NC-CAH, nonclassic phenotype; PCOS, polycystic ovary syndrome; SBP, systolic blood pressure; SDS, standard deviation score; SV, simple virilizing phenotype; SW, salt-wasting phenotype.

The presence of CAH seems also to affect fat distribution. Using computed tomography imaging, Kim et al showed that adolescents and young adults with CAH had increased amounts of both visceral and subcutaneous adipose tissue compared with age-, sex-, ethnicity-, and BMI-matched controls (90). Particularly the abdominal adiposity prevalence was increased in CAH (90). A higher proportion of the proinflammatory visceral than subcutaneous adipose tissue places patients with CAH at even greater risk for harmful metabolic sequelae from obesity (164). Moreover, others provided indirect evidence of abdominal obesity in CAH by showing increased waist circumference, percent truncal fat, visceral adipose tissue mass, visceral adiposity index, waist to hip ratio, and waist to height ratio (44, 50, 54, 62, 66, 88, 89, 104, 107, 113, 117). In 6 studies patients and controls statistically differed in BMI (50, 54, 104, 107, 113, 117), while in 3 studies the lack of such differences might have been attributed to the small sample size (44, 62, 89); however, the previously mentioned changes in fat distribution were also observed if patients with CAH and controls were deliberately matched for BMI (66, 88).

Interestingly, patients with CAH and elevated total testosterone concentrations also demonstrated an increased epicardial fat thickness (107), a parameter reflecting the amount of adipose tissue surrounding the heart and coronary vessels. Epicardial adipose tissue is a highly metabolically active structure and its thickening measured by echocardiography is a predictor of an increased risk of cardiovascular disease and metabolic syndrome (165). However, epicardial fat thickness was similar to that in healthy subjects in patients with CAH and good androgen control (53), and if individuals with CAH and controls were matched for BMI (69).

Children with C-CAH had an earlier age of adiposity rebound (36-40 months) than the general population (5-7 years), which was not associated with the treatment regimen (166-168). Adiposity rebound refers to a time point at which BMI begins to increase after its nadir (168). If it is early it is considered a predictive marker of obesity, overweight, and related metabolic diseases (metabolic syndrome, prediabetes and type 2 diabetes) in later childhood, adolescence, and adulthood (169).

Increased body weight is usually attributed to imperfections of glucocorticoid replacement (using supraphysiological doses, physiological doses with nonphysiological diurnal distribution or synthetic glucocorticoids) and/or to androgen excess (23). In a UK study, as many as 37% of men and 45% of women had 17OHP concentrations below 12 nmol/L (397 ng/dL), suggesting that supraphysiological glucocorticoid treatment may contribute to increased body weight (3). Glucocorticoid overtreatment in children, diagnosed based on complete suppression of androstenedione, was associated with BMI standard deviation score (SDS) above 0 and showing an increasing trend with time (65). Obese children with CAH were more often found to be on long-acting glucocorticoid therapy (which is not recommended in children) than their nonobese peers, while obesity in adults with CAH was more prevalent in those receiving higher doses of glucocorticoids (77). Moreover, the visceral adiposity index, an empirical mathematical model that has been proposed to assess fat distribution and function, correlated with glucocorticoid dose (88). However, most studies failed to document an association between increased BMI and glucocorticoid dose (41, 44, 48, 51, 56, 90, 117, 124, 136, 138, 142).

Type of glucocorticoid and specific glucocorticoid regimen affect body weight and fat distribution. Paizoni et al observed that body fat content was lower in individuals receiving hydrocortisone than synthetic glucocorticoids (121). Bouvattier et al reported that BMI in males with C-CAH was higher if they were treated with dexamethasone compared with hydrocortisone (56). Moreover, compared with hydrocortisone-treated patients, subjects receiving dexamethasone had increased waist circumference and fat mass (88). Obesity occurred more frequently in children with CAH receiving prednisolone, while overweight was more frequent in hydrocortisone-treated ones (38). In contrast, Han et al did not find differences in BMI and waist circumference between individuals with CAH receiving hydrocortisone, prednisolone, hydrocortisone together with prednisolone or dexamethasone (82). In 3 of these studies, details concerning doses of hydrocortisone and synthetic glucocorticoids were not provided, though Kara and colleagues declared that the doses were equivalent (82, 88, 121). In the remaining 2 studies, the authors did not statistically compare hydrocortisone equivalent doses but they might have been lower for dexamethasone than hydrocortisone in the study by Bouvattier et al (56), and higher for prednisone than hydrocortisone in the study by Abdel Meguid et al (38). Lastly, Seraphim et al did not observe changes in the prevalence of obesity (as well as in the prevalence of metabolic syndrome and hypertension) in subjects with CAH receiving low doses of dexamethasone (once daily at bedtime), who had earlier been treated with larger (by about 3 times) equivalent doses of cortisone acetate (133). Thus, differences in daily doses do not seem to explain well differences between natural and synthetic glucocorticoids in the impact on body weight and fat content. It should be remembered that both conventional hydrocortisone therapy and synthetic glucocorticoids, even if used in “physiologically” equivalent daily doses, fail to mimic the circadian rhythm of endogenous cortisol secretion, and result in periods of excessive glucocorticoid exposure (170, 171). In case of hydrocortisone, BMI *z*-score did not differ between prepubertal children receiving this agent twice or 3 times daily if the total daily doses were similar (43). Different glucocorticoid preparations and wide dose ranges that are used in patients with CAH, even in the same study, make it difficult to draw any strong conclusion on a relative risk of weight gain associated with long-term treatment with various glucocorticoid preparations. However, a meta-analysis of studies that compared at least 2 types of glucocorticoids found that BMI was lowest in hydrocortisone-treated patients with CAH and highest in dexamethasone-treated, with prednisolone-treated patients in between (172).

It is also possible that the impact of exogenous glucocorticoids on body weight and composition depends on transcriptional activity of the glucocorticoid receptor and may differ between various populations of patients with CAH (115). Genetic polymorphisms increasing glucocorticoid receptor sensitivity predispose to weight gain and obesity (173). Interestingly, patients with CAH and healthy controls were found to differ in the prevalence of at least 2 glucocorticoid receptor polymorphisms (*Bcl*I and *Tth111I*), which may suggest increased sensitivity of glucocorticoid substitution in patients with CAH (174, 175).

The question whether hyperandrogenism plays a role in the development of overweight and obesity in individuals with CAH is unclear. Although some studies reported that BMI and other obesity markers correlated with 17OHP and androstenedione concentrations (47, 77, 88, 113, 121), others failed to document such relationships (41, 46, 90, 138, 176). However, children and adolescents (of both sexes) with CAH were found to have correlations between epicardial fat thickness and 17OHP and total testosterone concentrations (107). Inverse correlations between the amount of visceral and subcutaneous adipose tissue and sex hormone–binding globulin concentrations suggest that changes in fat accumulation in CAH may be associated with a rise in free testosterone (90), the protein-unbound, biologically active fraction of testosterone. Unfortunately, the authors did not carry out separate analyses for males and females.

In a Swedish population-based cohort study, the greatest odds ratio for obesity was observed in subjects with NC-CAH (74). In a much smaller study, Wasniewska et al did not observe differences in BMI SDS and the waist to hip ratio between adolescents with C-CAH and NC-CAH (147). However, all participants in the latter study were treated with hydrocortisone, the consequence of which were similar 17OHP and androstenedione concentrations in both subgroups. Lastly, in the recent cross-sectional analysis, obesity was more prevalent in patients (both males and females) with C-CAH than in subjects with NC-CAH (mostly untreated) (59). We can only try to explain differences between these findings and the challenging results of the Swedish study. They may be associated with the fact that many individuals with NC-CAH receive glucocorticoids despite normal or only slightly reduced cortisol production, young women with NC-CAH are often prescribed potent and long-acting dexamethasone, increased BMI in patients with NC-CAH may reflect an increase in lean mass (secondary to prolonged androgen excess) (151), and bias because most patients with NC-CAH are never diagnosed (13).

Most studies reported no differences in BMI between individuals with SW-CAH and SV-CAH (41, 116, 143, 154). In contrast, Bonfig et al observed in a large cohort of 716 children and adolescents with CAH that BMI SDS was higher in SW-CAH than SV-CAH (51). Even if subjects with SW-CAH had increased body weight compared with SV-CAH, differences were small, which raises doubts whether they were clinically significant.

The risk to develop obesity rises with age and is greatest in individuals with CAH aged 30 years or older. Higher values of BMI are probably, at least in part, a consequence of increased lean mass in this group of patients, especially in females (70). Studies assessing whether sex determines body mass/composition in CAH have provided contrasting results. Some authors reported that fat content is increased in men but not women (52, 57, 60, 121). However, Bacila et al observed increased BMI SDS and waist circumference only in girls with CAH, while increased hip circumference was found only in boys aged 8-12 years (48). Moreover, in females, but not in males, CAH was associated with higher BMI and increased risk of obesity (101). Lastly, in the recent American study, obesity was more prevalent in women than in men, particularly in the subgroup with NC-CAH (59). Only in 1 study, were there no differences in BMI *z*-score and waist circumference between girls and boys with CAH (116). The reasons for this sexual dimorphism and between-study differences remain to be clarified. However, they may be associated with different hormonal profiles of males and females, different metabolic effects of androgens in both sexes, and/or with differences in lifestyle, physical activity, and glucocorticoid dose.

Since accurate body composition determinations are expensive and time-consuming, BMI and bioelectrical impedance analysis are often the only available measurements but have some limitations. BMI does not distinguish between fat mass and not-fat mass (muscle, bone, water and organs) (177). Thus, it does not inform about adipose tissue distribution (177) and may be affected by changes in the body's water volume, observed in case of inadequate glucocorticoid and mineralocorticoid replacement. Moreover, estimation of body composition using bioelectrical impedance analysis is determined by hydration status and glycogen levels (177).

In summary, patients with CAH seem to be at increased risk of developing overweight and obesity, associated with abdominal, and possibly also with epicardial, fat accumulation. The greater than expected gain in body weight already begun in childhood, may be more pronounced in subjects with NC-CAH, but there are no convincing data that it is determined by sex. The challenges of providing appropriate glucocorticoid replacement therapy while preventing excessive adrenal androgen production may contribute to unfavorable changes in body weight and body composition in individuals with CAH. Studies involving more physiological hormone replacement regimens as modified-release preparations or corticotropin-releasing factor type 1 receptor (CRF-1) antagonists that are currently in clinical trials and potentially allow reduction of glucocorticoid replacement doses will be essential to investigate the roles of glucocorticoids and androgens on these parameters.

## Glucose Homeostasis

The majority of studies conducted to date have documented impaired insulin action in individuals with CAH, independently of the method used for its assessment (Table 3). Although most researchers determined insulin sensitivity by measurement of homeostatic model assessment for insulin resistance index (HOMA-IR), some authors showed abnormal values of fasting insulin, glucose and insulin after oral glucose load, area under the curve for insulin or Δinsulin/Δglucose ratio after glucose load, and insulin sensitivity index (ISI_I0,120_). A limitation of most studies estimating insulin sensitivity was that they were based on surrogate markers. However, de Oliveira et al (61), Delai et al (66), and Kroese et al (94) observed lower values of the glucose infusion rate and the insulin sensitivity index in individuals with CAH than in healthy controls using the glucose clamp technique, which is the gold standard method for quantifying insulin secretion and action (178). Thus, the results of all 3 studies unanimously indicate that patients with CAH have reduced insulin sensitivity. Interestingly, a markedly reduced glucose infusion rate in patients with CAH was observed even if surrogate markers of insulin sensitivity did not differ from controls (66). Moreover, a meta-analysis of 12 longitudinal and 2 cross-sectional studies, including a group of 300 children/adolescents and 137 adults, showed that subjects with CAH have higher values of HOMA-IR, although no differences were observed for fasting insulin, fasting glucose, as well as for glucose and insulin levels 2 hours after oral glucose load (179). As Table 3 shows, increased risk of unfavorable changes in insulin sensitivity has been observed in studies including children, which may justify routine assessment of glucose homeostasis markers in the affected subjects already in the first decade of life. The degree of insulin sensitivity was similar (38), or more pronounced than in age-matched controls with obesity (83). Disturbances in glucose homeostasis seem to be more pronounced if CAH is poorly controlled, while less severe in subjects with well-controlled CAH (76, 106, 121).

**Table 3.** Glucose homeostasis in patients with congenital adrenal hyperplasia

Authors	Major findings	Conclusion (consequences for cardiometabolic health)^*a*^
Abdel Meguid et al (38)	No differences in fasting insulin, glucose, and HOMA-IR vs obese controls; positive correlation between HOMA-IR and glucocorticoid dose	Insulin sensitivity was similar in children with C-CAH and obese children (X)
Ahmed et al (39)	No differences in HOMA-IR and fasting glucose between patients treated with hydrocortisone or prednisone	Type of glucocorticoid had no impact on insulin sensitivity in children with C-CAH (X)
Akyürek et al (40)	HOMA-IR insignificantly higher vs controls; no differences in fasting glucose and insulin	C-CAH in children may be associated with reduced insulin sensitivity (I)
Amr et al (41)	HOMA-IR, fasting glucose, as well as glucose 30, 60, 90 and 120 minutes after oral glucose load higher vs controls; fasting insulin insignificantly higher vs controls; impaired fasting glucose in 34% of patients, impaired glucose tolerance in 19% of patients, insulin resistance in 34% of patients; glucose at 30 and 90 minutes after glucose load higher in SV than SW	C-CAH in children was associated with reduced insulin sensitivity and increased glucose concentration, and predisposes to prediabetes (I)
Ariyawatkul et al (44)	Fasting glucose lower vs controls; no differences in fasting insulin, HOMA-IR, and HbA_1c_; diabetes mellitus in 1 subject with CAH; no correlation between HOMA-IR and BMI, glucocorticoid dose, age, and 17OHP levels	C-CAH in children and young adults was associated with lower fasting glycemia (X)
Arlt et al (3)	Fasting hyperglycemia in 8% of patients (males with C-CAH: 6%, females with C-CAH: 7%, females with NC: 13%); insulin resistance (HOMA-IR>2.5): 29% of patients (males with C-CAH: 36%, females with C-CAH: 28%, females with NC: 24%)	High prevalence of reduced insulin sensitivity in adult population with CAH (I)
Bachelot et al (46)	Impaired glucose tolerance in 8% of patients; no cases of diabetes; correlations of HOMA-IR with BMI, 17OHP (even after adjusting for BMI) and testosterone (in women)	Young adults with CAH may develop prediabetes (X)
Bacila et al (48)	No differences in prevalence of insulin resistance vs controls; no differences in glucocorticoid dose between patients with high and normal HOMA-IR	C-CAH in children was associated with normal insulin sensitivity (N)
Bayraktar et al (49)	No differences in fasting glucose, fasting insulin, and HOMA-IR vs controls. Fasting glucose, fasting insulin, and HOMA-IR lower vs women with PCOS	Unaltered glucose homeostasis in women with NC-CAH (N)
Ben Simon et al (50)	No differences in fasting glucose vs controls	NC-CAH was not associated with changes in fasting glycemia in children (N)
Borges et al (52)	No differences in fasting glucose and insulin between CAH and controls, and between males and females with CAH; HOMA-IR not calculated	C-CAH was not associated with changes in fasting glycemia in young adults (N)
Borges et al (53)	No differences in fasting glucose, insulin, and HOMA-IR between men and women with CAH and control men and women	C-CAH was not associated with changes in glucose homeostasis in young adults (N)
Borges et al (54)	No differences in fasting glucose, insulin, and HOMA-IR between men and women with CAH and control men and women	C-CAH seemed to be not associated with changes in glucose homeostasis in young adults (N)
Bouvattier et al (56)	Hyperglycemia in 9.8% of patients with CAH; no cases of diabetes; mean fasting glucose within the reference range	Relatively high prevalence of hyperglycemia in patients with C-CAH (I)
Charmandari et al (58)	Insulin and HOMA-IR corrected for BMI higher than in control subjects; no differences in fasting glucose	C-CAH in children was associated with reduced insulin sensitivity (I)
Charoensri and Auchus (59)	Diabetes mellitus in 11% of patients; greatest prevalence (23.1%) between ages 40 and 49; no difference in the prevalence between males and females, and between CAH and NC-CAH	High prevalence of diabetes mellitus in adults with CAH (mainly in middle-aged ones) (I)
de Oliveira et al (61)	HOMA-IR and HbA_1c_ higher, insulin sensitivity index (ISI) lower vs controls; more insulin-resistant subjects with CAH than in controls; higher values of first phase insulin and total insulin, lower values of disposition index based on first phase and total (clamp 180 minutes) insulin secretory rate vs control subjects; reduced hepatic insulin clearance in subjects with CAH than in controls	C-CAH was accompanied by reduced insulin sensitivity in young adults (I)
de Vries et al (64)	No difference in fasting glucose, fasting insulin, and HOMA-IR between glucocorticoid-treated patients vs off-treated	Active glucocorticoid treatment did not affect glucose homeostasis in patients with NC-CAH (X)
Delai et al (66)	Fasting glucose, HbA_1c_, and glucose utilization/kg of fat-free mass/min (M_ffm_) in the hyperinsulinemic-euglycemic clamp lower vs controls, no differences in fasting insulin and HOMA-IR; lower M_ffm_ in dexamethasone- than cortisone acetate-treated patients; reverse correlation between M_ffm_ and duration of glucocorticoid treatment	NC-CAH was accompanied by reduced insulin sensitivity and possible periods of low glucose concentration (I)
Dubinski et al (68)	30% of patients with fasting hyperglycemia; higher mean glucose levels between 8 Pm and 8 Am than between 8 Am and 8 Pm in pubertal adolescents on reverse circadian glucocorticoid therapy; asymptomatic nocturnal hypoglycemia in 20% of patients; tissue glucose above 140-180 mg/dL in 50% of patients	C-CAH in children and adolescents was accompanied by high prevalence of fasting hyperglycemia, elevated glycemia in daily profile and asymptomatic nocturnal hypoglycemia (I)
Espinosa-Reyes et al (69)	Fasting glucose lower vs sex-, age- and BMI-matched controls; no difference in insulin, HOMA-IR, and percentage of insulin-resistant subjects	No conclusion can be drawn because of inconsistent finding (X)
Falhammar et al (70)	Lower fasting glucose in subjects <30 years old and insignificantly lower fasting glucose in subjects ≥30 years old vs controls; insignificantly higher fasting insulin in subjects ≥30 years old vs controls; higher fasting insulin in subjects ≥30 years old vs <30 years old; elevated fasting glucose or type 2 diabetes in 2 subjects ≥30 years old (3%); increased risk of gestational diabetes mellitus in subjects ≥30 years old vs controls	CAH may be associated with reduced insulin sensitivity in patients aged 30 years and older, and predisposes to gestational diabetes mellitus (I)
Falhammar et al (71)	Lower fasting glucose in nonobese subjects and in nonobese subjects <30 years old vs nonobese controls; insignificantly higher fasting insulin in nonobese subjects vs nonobese controls; higher fasting insulin and HOMA-IR in nonobese subjects ≥30 years old vs nonobese controls	CAH was associated with reduced insulin sensitivity in patients aged 30 years and older (I)
Falhammar et al (72)	No cases of diabetes, impaired glucose tolerance, and acanthosis nigricans; lower fasting glucose in subjects <30 years old vs controls; HbA1c insignificantly higher in patients ≥30 than <30 years old; increased area under the curve for insulin in oral glucose tolerance test in all patients; higher 2-hour insulin in patients ≥30 years old vs controls; higher area under the curve for insulin after oral glucose load in I172N group vs controls and vs null and I2G genotypes	CAH was associated with reduced insulin sensitivity in patients aged 30 years and older (I)
Falhammar et al (74)	Diabetes 3-times more prevalent vs sex-, year-, and place of birth-matched controls; increased risk in the entire cohort and in females; risk increased in women with SV, NC and with I172N genotype	High prevalence of diabetes in patients with CAH (I)
Falhammar et al (75)	No differences in prevalence of type 2 diabetes vs controls	CAH was not associated with increased risk of type 2 diabetes (N)
Farghaly et al (76)	Fasting glucose, fasting insulin, and HOMA-IR higher vs controls; HOMA-IR higher in patients with poor control than with good control	C-CAH was accompanied by increased fasting glycemia and reduced insulin sensitivity in children (I)
Finkielstain et al (77)	Higher HOMA-IR in children with C-CAH than NC; metabolic syndrome in 18% of adults and in 1 child with CAH; no association of metabolic syndrome with androgen concentrations, glucocorticoid type, and dose	High prevalence of metabolic syndrome in adults with CAH; the association of insulin sensitivity in children with type of CAH (I)
Green-Golan et al (80)	No differences in glucose vs controls; steady decline in glucose during exercise and increase during recovery in patients with CAH but not in controls; lack of overt hypoglycemia and glycopenic symptoms during exercise; no differences in insulin concentrations	Conclusions concerning glucose homeostasis in C-CAH difficult to draw because of inconsistent results (X)
Han et al (82)	HOMA-IR higher in subjects treated with dexamethasone than receiving hydrocortisone, prednisolone, and hydrocortisone/prednisolone combination; HOMA-IR higher if dexamethasone is administered once daily	Type of glucocorticoid determined insulin sensitivity in patients with CAH (X)
Harrington et al (83)	HOMA-IR higher vs controls but lower vs obese subjects	C-CAH was accompanied by slightly reduced insulin sensitivity in children (I)
Hashemi Dehkordi et al (84)	Elevated HOMA-IR in 29.3% of the affected patients; no correlations of HOMA-IR with 17OHP, DHEA-S, and testosterone concentrations	High prevalence of reduced insulin sensitivity in children with C-CAH (I)
Kara et al (88)	HOMA-IR higher vs BMI-, age- and gender-matched controls; no differences in HbA_1c_; no differences in HOMA-IR and HbA_1c_ between hydrocortisone- than dexamethasone-treated patients	C-CAH was accompanied by reduced insulin sensitivity (I)
Kępczyńska-Nyk et al (89)	Fasting glucose lower, fasting insulin higher vs healthy F; fasting glucose lower, HOMA-IR higher vs women with PCOS	CAH was associated with improved insulin sensitivity (D)
Kim et al (90)	Elevated HOMA-IR (adolescents >3.16, adults >2.5) in 18% of patients; correlation of HOMA-IR with amount of visceral and subcutaneous abdominal adipose tissue	C-CAH was accompanied by reduced insulin sensitivity in children and young adults (I)
Korkmaz et al (93)	Insulin and HOMA-IR correlated with SBP but not DBP; no comparison of insulin and HOMA-IR vs controls	Insulin sensitivity in children with CAH may determine SBP (X)
Kroese et al (94)	Lower values of glucose infusion rate and insulin sensitivity index vs controls; an increase in glucose infusion rate and insulin sensitivity index, a decrease in area under the curve for insulin in oral glucose tolerance test and an insignificant decrease in HOMA-IR after pioglitazone treatment; no changes in glucose and area under the curve for glucose in oral glucose tolerance test	CAH was accompanied by reduced insulin sensitivity in adults (improvement after pioglitazone) (I)
Krysiak et al (95)	Metformin-induced reduction in HOMA-IR, fasting glucose, and HbA_1c_ indifferent from women with normal adrenal function	Metformin improved insulin sensitivity in young adult women with NC-CAH (X)
Krysiak et al (98)	Higher HOMA-IR vs controls; no differences in fasting and 2-hour postchallenge glucose, positive correlations of HOMA-IR with 17OHP, DHEA-S, androstenedione, total testosterone, and free androgen index	NC-CAH was associated with reduced insulin sensitivity in young adult women with NC-CAH (I)
Lim et al (101)	Fasting glucose in males higher, HbA_1c_ in females lower vs respective controls; no differences in HbA_1c_ in men and fasting glucose in women	No conclusions concerning glucose homeostasis in C-CAH can be drawn because of inconsistent findings (X)
Liu et al (102)	Insulin resistance in 41.0% of patients, impaired glucose tolerance in 29.5% of patients, type 2 diabetes in 9.0% of patients, metabolic syndrome in 1 patient (1.3%); insulin resistance insignificantly more frequent in patients with 17OHP <2 ng/mL than ≥10 ng/mL	High prevalence of prediabetes, type 2 diabetes and all states associated with insulin resistance in young adult women with NC-CAH (I)
Marra et al (104)	Fasting glucose, fasting insulin, and HOMA-IR higher vs controls; the difference observed in both males and females; correlations between HOMA-IR and SBP and ΔSBP in response to exercise	C-CAH was accompanied by increased glucose concentration and reduced insulin sensitivity in children (I)
Metwalley et al (106)	Fasting glucose, fasting insulin, and HOMA-IR higher vs controls; HOMA-IR higher in patients with poorly controlled than with well-controlled CAH; correlations of fasting insulin and HOMA-IR with homocysteine, left ventricular mass index, carotid artery intima–media thickness, and mitral deceleration time	C-CAH, particularly poorly controlled, was accompanied by increased glucose concentration and reduced insulin sensitivity in children (I)
Metwalley et al (107)	Fasting glucose, fasting insulin, and HOMA-IR higher vs controls; correlation between HOMA-IR and epicardial fat thickness	C-CAH was accompanied by increased glucose concentration and reduced insulin sensitivity in children (I)
Mnif et al (109, 110)	Insulin resistance: 27.0%; carbohydrate metabolism disorders: 19.2% (impaired glucose tolerance: 15.4%, type 2 diabetes mellitus: 3.8%); metabolic syndrome: 3.8%; highest mean fasting insulin and HOMA-IR in subjects with SV and highest fasting and 2-hour postload glucose in subjects with NC (but no statistical comparisons were made)	High prevalence of insulin resistance states in patients with CAH (I)
Mooij et al (112)	No difference in fasting glucose, fasting insulin, and HOMA-IR vs controls	No association between CAH and impaired glucose homeostasis in adults (N)
Mooij et al (113)	Fasting glucose and HbA_1c_ within the reference range in all patients with CAH; median fasting insulin: 11.0 mU/L, median HOMA-IR: 2.64; HOMA-IR > 90th percentile in 29.6% of patients; correlations between HOMA-IR and daily hydrocortisone dose, renin, and BMI SDS	CAH is accompanied by reduced insulin sensitivity in children (I)
Moreira et al (115)	Metabolic syndrome in 7.3% of patients; HOMA-IR correlated with BMI. No differences in HOMA-IR between males and females, carriers and noncarriers of the *Bcl*I polymorphism, and between carriers of A3669G polymorphism and wild-type carriers	C-CAH may be associated with metabolic syndrome in children (X)
Moreira et al (116)	Fasting glucose lower, fasting insulin, and HOMA-IR higher vs controls; fasting insulin and HOMA-IR higher in girls than boys; no differences between SW and SV; metabolic syndrome in 12.2% of patients	C-CAH was accompanied by reduced insulin sensitivity in children (I)
Navardauskaite et al (117)	Fasting glucose lower, fasting insulin, and 2-hour postchallenge glucose higher in patients with CAH vs controls; elevated HOMA-IR more often in patients with CAH (62.5%) vs controls (18.8%); impaired fasting glucose in 1 patient (5%) with SW; impaired glucose tolerance in 3 patients (25%) with SV; no cases of metabolic syndrome in patients with CAH; inverse correlation between insulin and testosterone in patients with CAH; no correlation between glucose homeostasis markers and glucocorticoid type and dose	C-CAH was accompanied by reduced insulin sensitivity and may predispose to hyperglycemia in young adults (I)
Özdemir et al (120)	Fasting glucose, fasting insulin, and HOMA-IR within the reference range; no correlation with aortic stiffness and carotid artery intima–media thickness	CAH was accompanied by normal insulin sensitivity in children (N)
Paizoni et al (121)	Elevated fasting glucose and HOMA-IR >2.7 in 11.1% and 25.4% of patients respectively. Higher HOMA-IR in women with CAH, subjects with poor androgen control and with higher BMI; only 1 patient fulfilling criteria of metabolic syndrome	C-CAH was accompanied by reduced insulin sensitivity and may predispose to hyperglycemia in adults (I)
Pall et al (122)	No differences in markers of insulin sensitivity vs healthy subjects and lean women with PCOS; HOMA-IR, HOMA%B, fasting glucose and insulin, peak insulin, and area under the curve for glucose and insulin during oral glucose tolerance test and glucose 2 hours after glucose load lower than in obese women with PCOS	NC-CAH was accompanied by normal insulin homeostasis in adolescent girls and young adult women (N)
Paula et al (123)	Higher insulin 30 minutes after oral glucose load, higher Δinsulin/Δglucose ratio, insignificantly lower glucose uptake and nonoxidative utilization vs controls; no differences in glucose levels vs controls; insulin response to glucose load greater in C-CAH than in NC-CAH	CAH was accompanied by reduced insulin sensitivity in young adult women (I)
Rodrigues et al (128)	Glucose lower vs controls, no differences in fasting insulin and HOMA-IR	No conclusions concerning relevance of this finding can be drawn because of glycemia within the reference range (X)
Rosenbaum et al (129)	Fasting glucose lower vs controls; no differences in HOMA-IR	No conclusions concerning relevance of this finding can be drawn because of small between-group differences in glucose concentration (X)
Sartorato et al (130)	Fasting insulin and HOMA-IR higher than in controls; no differences in fasting glucose and in 2-hour postload glucose and insulin; no differences in metabolic control between SW and SV; no correlations between fasting and postload insulin and 17OHP, testosterone, and Δ_4_-androstenedione concentrations and cumulative doses of glucocorticoids	C-CAH was accompanied by reduced insulin sensitivity in young adults (I)
Saygili et al (131)	Fasting insulin, 2-hour postload insulin, and HOMA-IR higher vs controls; no differences in glucose; positive correlations of insulin with 17OHP and free testosterone concentrations	NC-CAH was accompanied by reduced insulin sensitivity in women (I)
Seraphim et al (133)	An increase in HOMA-IR after dexamethasone treatment; no changes in glucose levels	Dexamethasone reduced insulin sensitivity in C-CAH (X)
Speiser et al (134)	S_1_ lower than in controls and lower than expected for BMI; no correlation between S_1_ and 17OHP, testosterone Δ_4_-androstenedione, DHEA, and LH concentrations	NC-CAH was accompanied by reduced insulin sensitivity in women (I)
Subbarayan et al (136)	<10 years: fasting glucose and insulin, HOMA1-IR and HOMA%B lower than in historical controls; ≥10 years: HOMA-IR lower, fasting glucose and insulin insignificantly lower than in historical controls; values with normal limits; no differences in HOMA%B; HOMA-IR positively related to age but not to BMI SDS	No conclusions may be drawn concerning C-CAH in children and adolescents (X)
Torky et al (138)	Insulin resistance: 71.9% in childhood and 80.7% in adulthood (more frequently than in general population); fasting hyperglycemia: 75.4% in childhood and 45.6% in adulthood (only in childhood more frequently than in general population); metabolic syndrome: 40.4%; association of insulin resistance with % of daily glucocorticoid dose given at night but not with glucocorticoid dose and type; association of insulin resistance with obesity in childhood and with obesity and suppressed testosterone in adulthood; no association of metabolic syndrome with corticosteroid dose, genotype, phenotype, adult height SDS, maternal obesity, and androgens	High prevalence of fasting hyperglycemia, metabolic syndrome and reduced insulin sensitivity in children and adults with C-CAH (I)
Tuhan et al (140)	Glucose levels lower vs controls; the difference significant if males and females were compared separately and insignificant if both sexes were analyzed together; no difference between individuals with controlled and uncontrolled CAH	No conclusions concerning glucose homeostasis in C-CAH can be drawn because of inconsistent findings (X)
Virayan et al (142)	Fasting insulin and HOMA-IR higher, fasting glucose insignificantly lower vs controls; HOMA-IR correlated with SBP and DBP	C-CAH was accompanied by reduced insulin sensitivity in children and young adults (I)
Wasniewska et al (147)	HOMA-IR higher in subjects with C-CAH than in controls and insignificantly higher in subjects with C-CAH than in subjects with NC-CAH	CAH was accompanied by impaired insulin sensitivity in adolescents and young adults (I)
Weise et al (148, 149)	Short-term, high-intensity exercise does not increase whole-blood glucose concentration in patients with C-CAH; no differences in exercise-induced glucose and insulin concentrations between patients receiving single and double morning dose of hydrocortisone before exercising	Patients with C-CAH are characterized by impaired exercise-induced glycemic response, which cannot be corrected even by stress hydrocortisone doses (I)
Wierzbicka-Chmiel et al (150)	No differences in rate of metabolic syndrome vs controls	SW did not predispose to metabolic syndrome (N)
Williams et al (151)	All patients with CAH: slightly lower glucose, higher insulin/glucose ratio, 2-hour postload glucose and insulin, insulin response (0-30 minutes), and mean insulin (0-120 minutes) vs controls; no differences in insulin, HOMA%, insulin resistance index, and disposition index; patients with C-CAH: lower glucose vs controls; patients with NC-CAH: higher 2-hour postload glucose and insulin, insulin response (0-30 minutes), and mean insulin (0-120 minutes) vs controls	CAH may be associated with reduced insulin sensitivity in children (I)
Yoon and Cheon (152)	Impaired fasting glucose in 44.4% of patients >2 years old, more frequently in patients with high-risk genotype (large deletions/conversions) (71.4%) or in current high glucocorticoid dose group (64.3%) than current low glucocorticoid dose group (23.1%)	High prevalence of prediabetes in children and young adults with C-CAH (I)
Zhang et al (153)	Higher values of fasting insulin, HOMA-IR, 2-hour postload glucose, and the area under the curve of insulin, insignificantly higher values for the area under the curve of glucose, lower values of insulin sensitivity index (ISI) vs controls; no differences between both groups in fasting glucose, HOMA-β, and Δinsulin 30/Δglucose 30; increased risk of ≥1 component of metabolic syndrome; Insignificantly increased risk of ≥2 component of metabolic syndrome; no difference in rate of metabolic syndrome; correlation between HOMA-IR and adjusted testosterone	Untreated SV was accompanied by reduced insulin sensitivity in girls and young adult women (I)
Zimmermann et al (154)	Higher fasting glucose, fasting insulin, HOMA-IR, insulin resistance index (IRI) and HOMA-B, insignificantly lower insulin sensitivity index (ISI) vs controls; no differences between glucose and insulin 30 minutes, 60 minutes, 90 minutes and 120 minutes after glucose load, mean glucose and insulin, insulin/glucose ratio and C-peptide, and the area under the curve for glucose and insulin; no difference in glucose homeostasis between SW and SV; higher fasting insulin and HOMA-IR in genotype B vs genotype 0; insignificant correlations of fasting glucose with hydrocortisone equivalent dose and treatment duration but not with mean hydrocortisone dose; insignificant correlation between HOMA-IR and total hydrocortisone dose, significant correlations between IRI and total hydrocortisone dose and treatment duration; no correlation between glucose homeostasis markers and 17OHP, testosterone, and DHEA-S concentrations	C-CAH was accompanied by slightly reduced insulin sensitivity and increased glycemia (I)

^*a*^Consequences for cardiometabolic health: I, increased cardiometabolic risk; N, no impact on cardiometabolic risk; D, decreased cardiometabolic risk; X, no conclusions concerning cardiometabolic risk can be drawn based on these findings.

Summary: Unfavorable impact of CAH on glucose homeostasis in 42 studies (59.2%), no impact of CAH in 11 studies (15.5%), favorable impact of CAH in 1 study (1.4%), while 17 studies (23.9%) were inconclusive.

Abbreviations: 17OHP, 17-hydroxyprogesterone; ACTH, adrenocorticotropic hormone; BMI, body mass index; CAH, congenital adrenal hyperplasia; C-CAH, classic congenital adrenal hyperplasia; DBP, diastolic blood pressure; DHEA, dehydroepiandrosterone; DHEA-S, dehydroepiandrosterone-sulphate; HbA_1c_, glycated hemoglobin; HOMA-IR, homeostatic model assessment for insulin resistance index; NC, nonclassic phenotype; PCOS, polycystic ovary syndrome; SBP, systolic blood pressure; SDS, standard deviation score; SV, simple virilizing phenotype; SW, salt-wasting phenotype.

Prevalence of diabetes, prediabetes and metabolic syndrome in patients with CAH has been investigated in several studies. Analysis of the Swedish national registry revealed that diabetes (mainly type 2) was 3 times more frequent in subjects with CAH than in matched controls (74). Moreover, Mnif et al estimated the risk of type 2 diabetes at 3.8%, while impaired glucose tolerance was observed almost 4 times more frequently (109). In a pan-European study of 226 adults with CAH (almost all females), 2.3% had been diagnosed with type 2 diabetes, which was similar to general population (75). However, according to the authors of this study, the compared groups might have slightly differed in age and countries of origin. In the recent cross-sectional American study, the risk of diabetes has been estimated at 11%, and was highest (23.1%) in individuals aged between 40 and 49 years (59). Although the overall prevalence of diabetes in all these studies ranged from 2.3% to 11%, the mean or median age of study populations was low, ranging from 26 to 35 years (in the study with the highest prevalence). For comparison, the prevalence of young-onset type 2 diabetes in Europe, where many of these studies were conducted (59, 74, 75), is estimated at about 1% (180). Women with CAH were also found to have increased prevalence of gestational diabetes mellitus (5-21%) (33, 70, 181), which makes them more prone to the future development of type 2 diabetes (182). In a recent meta-analysis by Guo et al, the prevalence of gestational diabetes mellitus in females with CAH was estimated at 7.3%, which was a relative risk of 2.57 (183). The risk increased with age and was found to be more pronounced in women aged 30 years or older (70). Certainly, it cannot be totally excluded that increased prevalence of gestational diabetes mellitus reflected the impact of other factors, including comorbidities and concomitant therapies. This may explain why in a large United States database study, the increase of gestational diabetes in CAH disappeared after correction for confounding factors (mainly age and obesity) (184). In turn, a multicenter study of 244 adults with C-CAH showed type 2 diabetes in 7% and hyperinsulinemia without diabetes in 15% of the affected subjects treated for at least 1 comorbidity (163). Glucose-lowering agents, mainly insulin (19%) and metformin (75%), were prescribed to 16 individuals (11 patients with SW-CAH and 5 patients with SV-CAH), which was the second most common treatment in the studied population (only osteoporosis/osteopenia drugs were more frequently used). In the mentioned study, the median age of starting hypoglycemic treatment (mid-third decade) was much earlier than in general population (163). Lastly, impaired fasting glucose was present in 44.4% of Korean children and young adults with CAH, and its prevalence was particularly high if they were currently treated with hydrocortisone equivalent dose ≥13.63 mg/m^2^ daily (64.3%), or CAH was secondary to large gene deletions or conversions (71.4%) (152). The data concerning the risk of metabolic syndrome are also inconclusive because the percentage of individuals with CAH meeting the criteria of metabolic syndrome ranged from as little as 1.1% (121), if no routine screening was done, to as much as 40.4%, if patients were routinely screened (138). As in case of type 2 diabetes mellitus, impaired fasting glucose, impaired glucose tolerance and metabolic syndrome were diagnosed mainly in young patients. Thus, the presented data may underestimate their prevalence in the whole population of patients with CAH.

Worsened insulin sensitivity may be associated with many negative cardiometabolic consequences of CAH. Beyond the increased risk of carbohydrate metabolism disturbances, impaired insulin action in individuals with this disorder was found to be related to higher systolic (SBP) (104, 142) and diastolic blood pressure (DBP) (142), thickening of the carotid artery intima–media complex (107), as well as to increased left ventricular mass and diastolic dysfunction (107).

A few studies compared glucose homeostasis between various phenotypes and genotypes of CAH, suggesting that such an association may exist. Interestingly, with the exception of 1 study not reporting BMI (74), which is often a confounder for findings related to glucose and insulin metabolism, the analyzed subgroups did not differ in BMI (41, 151, 154), or when not formally analyzed by the authors, BMI seemed similar (72, 77, 110, 123). Williams et al observed that insulin sensitivity was more disturbed in subjects with NC-CAH than in individuals C-CAH (151). Paula et al observed a stronger insulin response to oral glucose load in C-CAH than in NC-CAH (123), while Finkielstain et al reported higher values of HOMA-IR in children with C-CAH than NC-CAH (77). Falhammar et al reported a greater area under the curve for insulin in patients with I172N than in the null and I2G genotype groups (72). In another study of the same research team, the authors observed increased prevalence of diabetes (mostly type 2) in women with SV-CAH (as well as I172N genotype) and NC-CAH (74). A greater risk to develop diabetes in milder forms of CAH may result from overtreatment in these groups of patients. In line with this explanation, despite various enzyme activity, Swedish patients with different phenotypes and genotypes were treated with similar doses of glucocorticoids (72). Lastly, in the study by Zimmermann et al, though there were no differences in glucose homeostasis markers between SW-CAH and SV-CAH, subjects with I172N and P30L genotype showed higher values of fasting insulin and HOMA-IR than those with null genotype (154).

Only 2 studies (of the same research group) investigated the potential role of the glucocorticoid receptor polymorphisms. Both, including adults with C-CAH, did not show differences in HOMA-IR between carriers and noncarriers of the *Bcl*I polymorphism, as well as between carriers of A3669G polymorphism and wild-type carriers (115, 185). These findings may suggest a less important role of differences in glucocorticoid receptor activity for glucose homeostasis than for determination of body mass and composition, plasma lipids and BP.

Despite correlations with BMI (113, 115, 121), and with the amount of abdominal adipose tissue (both visceral and subcutaneous) (90), impaired insulin sensitivity cannot be regarded only as a simple consequence of increased fat content. Disturbances in glucose homeostasis were observed not only in obese but also in nonobese subjects with CAH (61, 71). The insulin sensitivity marker (S_1_) in individuals with CAH was found to be lower than expected for BMI (134). Lastly, individuals with CAH treated with glucocorticoids were less insulin-sensitive than healthy controls, although both groups were matched for BMI (94). Moreover, the half-life of insulin in CAH may be prolonged (61).

It seems that impaired insulin sensitivity in subjects with CAH may be in part associated with either overtreatment or, at least in women, with using glucocorticoids in doses which are insufficient to normalize ACTH secretion and androgen production (186). In the case of overtreatment, the mechanism of impaired insulin action is similar to that observed in Cushing syndrome, while in the second, the mechanism resembles that in polycystic ovary syndrome (PCOS) (187). The correlations between insulin resistance index and total hydrocortisone dose, and between insulin resistance index and treatment duration, as well as of a weak correlation between HOMA-IR and total hydrocortisone dose are in favor of the association of impaired insulin sensitivity in CAH with the cumulative effect of long-term glucocorticoid treatment (154). Correlations between insulin sensitivity and glucocorticoid dose and treatment duration were also recently observed by others (38, 66). These findings justify avoiding supraphysiological glucocorticoid doses. A greater risk to develop diabetes in women than men with CAH may be associated with using higher doses of glucocorticoids by females, in whom symptoms of androgen excess are more pronounced than in males (74). In contrast, Bacila et al observed that patients with high and normal values of HOMA-IR did not differ in current glucocorticoid dose (48). There were no correlations between insulin sensitivity and current as well as cumulative doses of glucocorticoids, although in some studies daily hydrocortisone equivalent doses were within recommended guidelines (40, 44, 77, 117, 138). Moreover, fasting glucose and insulin as well as HOMA-IR did not differ between patients with NC-CAH on glucocorticoids or not (64). This incongruency may be partially explained by the intermittent glucocorticoid excess in patients on conventional glucocorticoid replacement. Moreover, blood sampling was performed in early hours before intake of the morning dose and therefore morning values of glucose homeostasis markers may not reflect daily glucocorticoid supplementation. There may also be differences between various glucocorticoids in their impact on glucose homeostasis. Firstly, despite greater suppression of androgen production compared with subjects receiving prednisolone or hydrocortisone, dexamethasone-treated individuals with CAH were more insulin-resistant (82). Secondly, chronic treatment with dexamethasone at bedtime was associated with an increase in HOMA-IR (133). Thirdly, insulin sensitivity assessed using the clamp was lower in subjects receiving dexamethasone than cortisone acetate (66). However, some studies did not show the association between insulin sensitivity and glucocorticoid type (77, 88, 117, 138). The risk of disturbances in glucose homeostasis may also depend on diurnal variation in glucocorticoid levels. In line with this explanation, impaired insulin sensitivity correlated with a percentage of daily dose given at bedtime (125). Moreover, reduced insulin sensitivity was more likely if dexamethasone was administered once than twice daily, which may result from its higher peak levels in case the drug was given as single dose at night (82). Lastly, pubertal adolescents with CAH on reverse circadian glucocorticoid therapy had higher mean glucose levels between 8 Pm and 8 Am than between 8 Am and 8 Pm (68).

The role of androgen excess in deterioration of glucose metabolism is supported by the correlations between markers of insulin sensitivity and free and total testosterone concentrations (98, 131). Moreover, correlations between HOMA-IR and testosterone concentrations were observed after adjustment for plasma glucose, BMI, plasma lipids, plasma adiponectin, and BP (153). The role of elevated androgen concentrations is also supported by finding impaired insulin sensitivity in untreated subjects with NC-CAH (131, 133) or SV-CAH (153). The lack of association between insulin sensitivity and androgen concentrations in adolescents may result from the fact that the impact of these hormones on glucose homeostasis may overlap with the impact of puberty itself (188). Worsening of insulin sensitivity in this age group is probably secondary to the increase in gonadal steroid production and an indirect consequence of physiologically increased growth hormone secretion (134). Moreover, a single measurement in the morning instead of data from several time points may not reflect daily androgen production with a typical circadian rhythm (121). It is likely that impaired insulin action and hyperandrogenemia are a part of the vicious circle observed in CAH. Insulin excess was found to induce adrenal steroid production, and this effect is attributed to stimulation of 17,20-lyase and 17α-hydroxylase activities (189). The resultant increase in androgen concentrations may lead to disturbances in menstruation, as well as to polycystic ovary morphology on ultrasonography, which are frequent findings in women with CAH (190).

There are arguments suggesting that impaired insulin action in individuals with CAH is reversible and may improve after metformin and pioglitazone (peroxisome proliferator–activated receptor-γ agonist available only in some countries). A beneficial effect of metformin therapy on insulin sensitivity was observed in Polish women with NC-CAH (95). The metformin-induced decrease in HOMA-IR was accompanied by a reduction in androgen concentrations (95), which suggests that metabolic and hormonal changes associated with the treatment are reciprocally related. This finding may be explained by the fact that metformin dose-dependently reduces activities of 17α-hydroxylase/17,20 lyase and 3β-hydroxysteroid dehydrogenase, 2 key enzymes in androgen biosynthesis (191). Metformin may also prevent androgen excess by an inhibitory effect on expression of MCR-2 receptor, mediating the impact of ACTH on adrenal steroidogenesis (192). In turn, pioglitazone increased glucose infusion rate and insulin sensitivity index, and decreased the area under the curve for insulin in the oral glucose tolerance test. However, the drug only tended to reduce HOMA-IR, and did not affect glucose concentration in the oral glucose tolerance test (94).

Unlike the majority of studies, some authors reported that patients with CAH had reduced fasting glucose concentration (44, 69-71, 116, 129, 136, 140, 142), which seems to be associated with adrenomedullary failure (which is a biomarker of disease severity) (72, 193, 194), though the involvement of early-morning cortisol insufficiency cannot be completely ruled out (22, 195). The close proximity of the adrenal cortex and medulla and possible underdevelopment of the latter structure in C-CAH cause that exogenous glucocorticoids may not normalize adrenal medulla secretory function, leading to an increase in insulin secretion, and subsequently to low glucose levels. Moreover, adrenomedullary failure and, though less likely, cortisol insufficiency may explain asymptomatic nocturnal hypoglycemia reported recently in as many as 20% of children and adolescents with C-CAH on continuous glucose monitoring (68). Lastly, decreased epinephrine reserve, but not glucocorticoid undertreatment, may be responsible for a decline in plasma glucose levels during prolonged moderate-intensity exercise in patients with CAH, which is absent in controls (80), as well as for defective glucose elevation in response to high-intensity exercise (148, 149). It seems that hypoglycemia in adults, unrelated to treatment of diabetes, may per se be a factor increasing cardiometabolic risk. Even mildly reduced glucose concentrations were associated with impaired endothelial function, enhanced proinflammatory response, platelet activation, enhanced coagulation, and impaired fibrinolysis (196). Moreover, mild hypoglycemia was related to an increase in the prevalence in ischemic heart disease (197), and to worse outcomes including increased mortality in acute cardiac events (198). Thus, long-term consequences of subnormal glucose concentration in individuals with CAH require further research.

In summary, patients with CAH may have reduced insulin sensitivity. Individuals with CAH seem to be also at high-risk of early-onset type 2 diabetes, gestational diabetes, and other disturbances of glucose homeostasis. These complications can be only partially explained by increased body weight and changes in adipose tissue distribution. They are also determined by the degree of 21OH deficiency, are partially related to imperfections of the glucocorticoid replacement and may improve after treatment with insulin-sensitizing drugs. Some subjects with CAH may, however, develop morning, nocturnal and maybe also exercise-induced hypoglycemia, with potential negative cardiovascular consequences.

## Plasma Lipids

Studies measuring circulating lipid levels have provided contrasting results, which may be associated with the fact that most of them included only a limited number of participants (Table 4). Falhammar et al did not observe differences in plasma lipids between adult women with CAH and healthy controls younger than 30 years (70). In women with CAH aged 30 years old or older, they found higher values of the high-density lipoprotein (HDL)/low-density lipoprotein (LDL) cholesterol ratio and a tendency to higher HDL concentrations than matched controls (70). In adult men with CAH, the same authors reported similar concentrations of total cholesterol, LDL cholesterol, HDL cholesterol, triglycerides, and lipoprotein (a) compared with matched controls (72). In men with CAH aged 30 years or more, levels of HDL cholesterol and triglycerides were higher while the HDL/LDL cholesterol ratio was lower than in younger men with CAH, even after excluding men treated with statins (72). In the United Kingdom Congenital Adrenal Hyperplasia Adult Study (CaHASE), total cholesterol levels above 5.0 mmol/L (193 mg/dL) were observed in 36% of men and 48% of women with C-CAH, LDL cholesterol levels above 3.0 mmol/L (116 mg/dL) in 35% of men and 37% of women with C-CAH, HDL cholesterol below 1.0 mmol/L (39 mg/dL) in 14% of men with C-CAH, and HDL cholesterol below 1.2 mmol/L (46 mg/dL) in 8% of women with C-CAH (3). In a study of 244 patients with CAH (183 with C-CAH, 61 with NC-CAH), elevated cholesterol levels were reported in 2% of children and 6% of adults, while decreased HDL cholesterol levels were found in approximately 10% of children and 15% of adults (77). The authors did not perform a separate analysis for C-CAH and NC-CAH. In a European study, dyslipidemia, though observed in only 3.7% of patients with CAH, was reported more frequently than in controls (0.4%) (75). Moreover, Paizoni et al reported a high prevalence of abnormalities in the lipid profile in adult men and women with C-CAH. However, only triglyceride levels were independently associated with androstenedione concentrations. Unfortunately, many patients (44% of men and 63% of women) were either undertreated or overtreated, subjects with SV-CAH received relatively high glucocorticoid doses, low and high lipid levels were not defined, and there was no data on plasma lipids in the control group (121). In turn, in a recent UK study including 101 patients with C-CAH aged 8-18 years, elevated total cholesterol, low HDL cholesterol, high LDL cholesterol, and high triglycerides were found in, respectively, 3%, 3%, 7%, and 5% of patients (48). In another study, criteria of dyslipidemia were met by 33.3% of Korean children and young adults with C-CAH (152). In the recent study by Charoensri and Auchus, dyslipidemia was present in 15% of patients with CAH, with no difference between C-CAH and NC-CAH, and between both sexes. Although dyslipidemia was significantly associated with the prevalence of cardiovascular diseases in univariable analysis, this relationship was not observed in the adjusted model (59). Lastly, the meta-analysis by Tamhane et al showed no differences in concentrations of total cholesterol, LDL cholesterol, HDL cholesterol, and triglycerides between 437 patients with CAH receiving replacement therapy with glucocorticoids and/or mineralocorticoids and control subjects (179). The inconsistent results of smaller cohort studies, mentioned in Table 4, no difference compared with control subjects in many studies, and frequent prevalence of dyslipidemia in non-CAH individuals suggest that the impact of CAH on plasma lipids is limited. A recent structured questionnaire sent to leading expert centers managing adults with CAH showed the use of lipid-lowering agents (mainly statins) in 17 out of 244 patients (7%), which was greater in men (9%) than in women (5%) (163). Even considering that only 65% of the centers screened for dyslipidemia by assessing fasting lipids, it seems that dyslipidemia is an uncommon complication in CAH.

**Table 4.** Plasma lipids in patients with congenital adrenal hyperplasia

Authors	Major findings	Conclusion (consequences for cardiometabolic health)*^a^*
Abdel Meguid et al (38)	LDL cholesterol >2.7 mmol/L more frequently in patients with CAH (26.7%) than obese controls (7.5%); no differences in the remaining lipid fractions vs obese controls; LDL cholesterol higher in prednisolone- than hydrocortisone-treated patients	C-CAH in children was associated with higher LDL cholesterol concentrations (I)
Ahmed et al (39)	Higher LDL levels in prednisone- vs hydrocortisone-treated subjects; no differences in total cholesterol, HDL cholesterol, and triglycerides	Type of glucocorticoid may partially determine LDL cholesterol concentrations in children with C-CAH (X)
Akyürek et al (40)	Dyslipidemia present in 12% of patients; no difference in total cholesterol, LDL cholesterol, HDL cholesterol, and triglycerides vs controls	Some children with SW had dyslipidemia (but it was difficult to say whether more often than in their peers) (X)
Amr et al (41)	No difference in total cholesterol, LDL cholesterol, HDL cholesterol, and triglycerides vs controls; no differences in lipid profile SW vs SV	C-CAH was not accompanied by changes in lipid concentrations in children (N)
Ariyawatkul et al (44)	HDL/total cholesterol ratio lower vs controls; no difference in total cholesterol, LDL cholesterol, HDL cholesterol, and triglycerides	C-CAH may be accompanied by more atherogenic lipid profile in children and young adults (I)
Arlt et al (3)	Total cholesterol >5.0 mmol/L: 46% (C-CAH men: 36%, C-CAH women: 48%, NC women: 59%); LDL cholesterol >3.0 mmol/L: 39% (C-CAH men: 37%, C-CAH women: 35%, NC women: 60%); HDL cholesterol <1.0 mmol/L: C-CAH men: 14%; HDL cholesterol <1.2 mmol/L: C-CAH women: 8%, NC women: 7%	Dyslipidemia was frequently found in adults with CAH (but it is difficult to say whether more often than in the general population) (X)
Bacila et al (48)	Raised total cholesterol: 3% of patients; low HDL cholesterol: 3% of patients, raised LDL cholesterol: 7% of patients, raised triglycerides: 5% of patients	Some children with C-CAH have dyslipidemia (but it was difficult to say whether more often than in their peers) (X)
Bayraktar et al (49)	No difference in total cholesterol, LDL cholesterol, HDL cholesterol, triglycerides, and lipoprotein (a) vs controls; higher HDL cholesterol and lower triglycerides vs women with PCOS	NC-CAH was not accompanied by changes in lipid levels in young adult women (N)
Ben Simon et al (50)	No differences in total cholesterol, HDL cholesterol, non-HDL cholesterol, LDL cholesterol, and triglycerides vs controls	NC-CAH in children did not predispose to changes in plasma lipids (N)
Borges et al (52)	Higher LDL/HDL cholesterol ratio vs controls in females but not males; no difference in total cholesterol, LDL cholesterol, HDL cholesterol, and triglycerides vs controls	C-CAH may be accompanied by more atherogenic lipid profile in young adults (I)
Botero et al (55)	Higher triglyceride levels vs controls; no difference in total cholesterol, LDL cholesterol, HDL cholesterol, and HDL/total cholesterol ratio; a higher percentage of subjects with triglycerides over the cut-off point vs controls	CAH was accompanied by higher triglyceride concentrations (I)
Bouvattier et al (56)	Mean total cholesterol and triglycerides within the reference range	C-CAH did not predispose to changes in plasma lipids (N)
Charoensri and Auchus (59)	Dyslipidemia in 15% of all patients with CAH, 42.1% of patients with concomitant cardiovascular disease and 12.8% of patients without cardiovascular disease; no difference in the prevalence between males and females, and between CAH and NC-CAH; positive association with the prevalence of established cardiovascular disease only in univariable analysis but not in the adjusted model	Dyslipidemia was frequently found in adults with CAH (but it is difficult to say whether more often than in the general population) (X)
de Oliveira et al (61)	No difference in total cholesterol, LDL cholesterol, HDL cholesterol, and triglycerides vs controls; triglyceride levels insignificantly higher in SV than SW; no difference in total cholesterol, LDL cholesterol, and HDL cholesterol between SW and SV	C-CAH was not accompanied by changes in plasma lipids in young adults with CAH (N)
de Vries et al (64)	No difference in total cholesterol, LDL cholesterol, HDL cholesterol, and triglycerides between glucocorticoid treated patients with NC vs off-treated; association between HDL cholesterol and glucocorticoid treatment; association between LDL cholesterol and current glucocorticoid dose	Glucocorticoid dose may have some (probably small) effect on lipid levels in children with NC-CAH (X)
Delai et al (66)	Lower HDL cholesterol vs controls; no differences in total cholesterol, LDL cholesterol, and triglycerides	NC-CAH predisposed to low HDL cholesterol (I)
Espinosa-Reyes et al (69)	Triglycerides and total cholesterol/HDL cholesterol ratio (atherogenic index) higher *vs* sex-, age-, and BMI-matched controls; no difference in total, LDL, and HDL cholesterol	CAH in children and young adults was accompanied by more atherogenic lipid profile (I)
Falhammar et al (70)	<30 years: No difference in total cholesterol, LDL cholesterol, HDL cholesterol, triglycerides, and HDL/LDL cholesterol ratio vs controls; ≥30 years: higher HDL/LDL cholesterol ratio and insignificantly higher HDL cholesterol vs controls; total and HDL cholesterol higher in women with CAH ≥30 years than <30 years	No conclusions can be drawn based on inconsistency in results (X)
Falhammar et al (72)	No difference in total cholesterol, LDL cholesterol, HDL cholesterol, triglycerides, lipoprotein (a), and HDL/LDL cholesterol ratio vs controls; higher LDL cholesterol and triglycerides, lower HDL/LDL cholesterol ratio in men with CAH ≥30 years than <30 years	CAH was not accompanied by changes in lipid concentrations (N)
Falhammar et al (74)	Greater risk of dyslipidemia in the whole cohort, especially in subjects with SW and males with null genotype	CAH predisposed to dyslipidemia (I)
Falhammar et al (75)	Dyslipidemia more prevalent vs controls	CAH predisposed to dyslipidemia (I)
Farghaly et al (76)	No difference in total cholesterol, LDL cholesterol, HDL cholesterol, and triglycerides vs controls	C-CAH was not accompanied by changes in plasma lipids in children (N)
Finkielstain et al (77)	Children—elevated cholesterol: 2%, decreased HDL: 10%; adults—elevated cholesterol: 6%, decreased HDL: 15%	Some patients with CAH had dyslipidemia (but it was difficult to say whether more often than their peers) (X)
Harrington et al (83)	No difference in total cholesterol, LDL cholesterol, HDL cholesterol, and triglycerides vs controls	C-CAH was not accompanied by changes in lipid concentrations in children (N)
Kara et al (88)	Triglycerides lower vs BMI-, age-, and gender-matched controls; no differences in HDL and LDL cholesterol; triglycerides lower in hydrocortisone- than dexamethasone-treated patients	C-CAH was accompanied by lower triglyceride concentrations (D)
Kępczyńska-Nyk et al (89)	No differences in HDL cholesterol and triglycerides vs healthy women and women with PCOS.	C-CAH was not accompanied by changes in plasma lipids in women with CAH (N)
Kim et al (90)	Correlations between total cholesterol, LDL cholesterol, very low-density lipoprotein cholesterol, and triglycerides and amount of visceral and subcutaneous fat	Fat content and distribution may determine lipid concentrations in children and young adults with C-CAH (X)
Kim et al (91)	Higher HDL cholesterol and lower triglycerides vs controls; no difference in total and LDL cholesterol	C-CAH may be associated with increased HDL cholesterol concentrations in children and young adults (D)
Korkmaz et al (93)	No difference in total cholesterol, LDL cholesterol, HDL cholesterol, and triglycerides vs controls	CAH was not accompanied by changes in lipid concentrations in children (N)
Krysiak et al (95)	No differences in metformin action on plasma lipids (reduction in triglycerides) vs controls	NC-CAH did not determine metformin action on plasma lipids in young adult women (X)
Krysiak et al (96)	Impact of simvastatin on plasma lipids (reduction in total and LDL cholesterol, no changes in HDL cholesterol and triglycerides similar in metformin-treated women with NC and controls	NC-CAH did not determine simvastatin action on plasma lipids in young adult women (X)
Krysiak et al (97)	A decrease in total and LDL cholesterol after treatment with atorvastatin; no effect of treatment on HDL cholesterol and triglycerides	Atorvastatin improved plasma lipids in young adult women with NC-CAH (X)
Krysiak et al (98)	Higher triglycerides and lower HDL cholesterol vs controls; no differences in total and LDL cholesterol	NC-CAH was associated with atherogenic changes in plasma lipids in young adult women (I)
Lim et al (101)	Males: HDL cholesterol higher vs respective controls; Females: total cholesterol, HDL cholesterol, and triglycerides higher vs respective controls	Because of inconsistent results, it was difficult to draw conclusions concerning lipid concentrations in young adults with C-CAH; possible between-sex differences (X)
Liu et al (102)	Dyslipidemia in 32.1% of patients; total cholesterol lower in patients with 17OHP <2 ng/mL than ≥10 ng/mL, no differences in LDL cholesterol, HDL cholesterol, and triglycerides	Dyslipidemia was frequently observed in young women with NC-CAH (but it is difficult to say whether more often than in their peers) (X)
Marra et al (104)	No difference in total cholesterol, LDL cholesterol, HDL cholesterol, and triglycerides vs controls; similar concentrations in men and women	C-CAH was not accompanied by changes in plasma lipids in children (N)
Metwalley et al (106, 107)	Higher total cholesterol, LDL cholesterol, and triglycerides, lower HDL cholesterol vs controls	C-CAH was accompanied by changes in plasma lipids in children (I)
Mnif et al (109, 110)	Alternations in lipid profile in 38.4% of patients: isolated hypercholesterolemia in 2 patients (7.7%), isolated hypertriglyceridemia in 3 patients (11.5%), isolated low HDL cholesterol in 5 patients (19.2%); higher total cholesterol levels in patients with NC than SW; no differences in total cholesterol between SV and SW, and between SV and NC; no differences in HDL cholesterol and triglycerides between SW, SV, and NC	Dyslipidemia was frequently observed in patients with CAH (but it is difficult to say whether more often than in the general population) (X)
Mooij et al (112)	HDL cholesterol higher vs controls; no difference in total cholesterol, LDL cholesterol, and triglycerides	CAH was accompanied by higher HDL cholesterol concentrations (D)
Mooij et al (113)	Total cholesterol, LDL cholesterol, and triglycerides below 50th percentile in 63-78% of patients with CAH; HDL cholesterol >50th percentile in 52% of patients; negative correlation between total cholesterol and androstenedione; no associations with therapy control, hydrocortisone dose, BMI SDS, and HOMA1-IR	Because of inconsistent results, it was difficult to draw conclusions concerning lipid concentrations in children with CAH (X)
Moreira et al (115)	Higher LDL cholesterol and triglycerides, lower HDL cholesterol in men with CAH vs women with CAH; low HDL cholesterol in 30% patients, more frequently in SW than SV; increased triglycerides in 10% of patients; higher triglycerides in *Bcl*I. heterozygous carriers than *Bcl*I homozygous carriers	C-CAH in adults was accompanied by decreased HDL cholesterol, and is characterized by sex-dependent differences in lipid concentrations (I)
Moreira et al (116)	Insignificantly lower triglycerides and HDL cholesterol vs controls; dyslipidemia in 70% of patients vs 45% in controls; no difference in total cholesterol, LDL cholesterol, HDL cholesterol, and triglycerides between girls and boys with CAH; total cholesterol, LDL cholesterol, and triglycerides higher in SW than in SV	High prevalence of dyslipidemia in children with C-CAH (more frequent than in their peers) (I)
Navardauskaite et al (117)	Triglycerides higher in patients with CAH vs controls; total cholesterol higher, HDL cholesterol lower in patients with SW vs patients with SV; correlation between glucocorticoid dose and LDL cholesterol in men and triglycerides in women; no correlation between lipid levels and glucocorticoid type; total cholesterol and LDL cholesterol in patients with Null/Null and Null/A genotypes lower vs remaining genotypes	C-CAH was accompanied by higher triglyceride concentrations, and lipid profile may be worse in SW than in SV (I)
Özdemir et al (120)	No difference in total cholesterol, LDL cholesterol, HDL cholesterol, and triglycerides vs controls	CAH was not associated with changes in lipid concentrations in children (N)
Paizoni et al (121)	High total cholesterol in 62% and 50%, high LDL cholesterol in 32% and 36%, high triglycerides in 79% and 93% and low HDL cholesterol in 14% and 4% of, respectively, men and women with CAH; no differences in abnormal lipid levels between affected men and women; correlations between LDL cholesterol and percentage body fat, between HDL cholesterol and female sex, and between androstenedione and triglycerides	Dyslipidemia was frequently observed in adults with C-CAH (but it was difficult to say whether more often than in the general adult population) (X)
Rodrigues et al (128)	HDL cholesterol lower vs controls; no difference in total cholesterol, LDL cholesterol, and triglycerides	C-CAH was associated with decreased HDL cholesterol concentrations in children and young adults (I)
Rosenbaum et al (129)	LDL cholesterol higher vs controls; no difference in total cholesterol, HDL cholesterol, and triglycerides	CAH was associated with elevated LDL cholesterol concentrations (I)
Sartorato et al (130)	No difference in total cholesterol, HDL cholesterol, and triglycerides vs controls	C-CAH was not associated with changes in lipid concentrations in young adults (N)
Subbarayan et al (136)	Elevated triglycerides in 9.5% of patients with CAH, high cholesterol in 3% of patients with CAH; no differences between girls and boys; no differences between SW and SV; no correlations with age, BMI SDS, blood pressure, and hydrocortisone dose	C-CAH in children and young adults may be accompanied by dyslipidemia (but it is difficult to say whether more often than in their peers) (X)
Torky et al (138)	Childhood: increased risk of low HDL cholesterol; adulthood: insignificantly higher total cholesterol and insignificantly lower LDL cholesterol; inverse correlation between 17OHP and high cholesterol in childhood; positive correlation between 17OHP and HDL cholesterol in childhood; inverse correlation between HDL and mineralocorticoid dose in childhood; inverse correlation between androstenedione and LDL cholesterol in adulthood	Association between C-CAH and lipid concentrations depended on age (X)
Tuhan et al (140)	No difference in total cholesterol, LDL cholesterol, HDL cholesterol, and triglycerides vs controls	C-CAH was not associated with changes in lipid concentrations in children (N)
Virayan et al (142)	Insignificantly lower HDL cholesterol vs controls; no difference in total cholesterol and LDL cholesterol	C-CAH was probably not associated with changes in lipid concentrations in children and young adults (N)
Wasniewska et al (147)	Higher HDL cholesterol in C-CAH vs controls; higher LDL cholesterol in NC vs controls; no difference in total cholesterol, triglycerides, and triglyceride/HDL ratio	Because of inconsistent results, it was difficult to draw conclusions concerning lipid concentrations in adolescents and young adults with CAH (X)
Wierzbicka-Chmiel et al (150)	No difference in total cholesterol, LDL cholesterol, HDL cholesterol, and triglycerides vs controls; dyslipidemia in 53% of patients vs 45% in controls	SW was not associated with changes in lipid concentrations in young adults (N)
Williams et al (151)	HDL cholesterol higher and total cholesterol/HDL cholesterol ratio lower in patients with C-CAH vs controls; no difference in total cholesterol, LDL cholesterol, HDL cholesterol, and triglycerides in subjects with NC vs controls	CAH may be associated with favorable changes in lipid concentrations in children (D)
Yoon and Cheon (152)	Dyslipidemia in 33.3% of patients >2 years old, more frequently on low (53.8%) than high (14.3%) average daily glucocorticoid dose	Dyslipidemia was often diagnosed in children and young adults with C-CAH (I)
Zhang et al (153)	HDL cholesterol lower while triglycerides higher vs controls; positive correlation between triglycerides and 17OHP and inverse correlation between HDL cholesterol and 17OHP; after adjustment for BMI, positive correlation between triglycerides and testosterone and inverse correlation between HDL cholesterol and testosterone	Untreated SV was accompanied by unfavorable changes in HDL cholesterol and triglycerides in girls and young adult women (I)
Zimmermann et al (154)	Higher small dense LDL and lower HDL cholesterol vs controls; no difference in total cholesterol, triglycerides, and LDL/HDL cholesterol ratio; insignificantly higher triglycerides in SW than SV; higher small dense LDL in I2G genotype than null genotype; no correlation between lipids and 17OHP, testosterone and DHEA-S	C-CAH may be associated with unfavorable changes in lipids in children and young adults (I)

Summary: Unfavorable impact of CAH on plasma lipid concentrations in 18 studies (31.0%), no impact of CAH in 16 studies (27.6%); favorable impact of CAH in 4 studies (6.8%), while 20 studies (34.5%) were inconclusive.

Abbreviations: 17OHP, 17-hydroxyprogesterone; BMI, body mass index; CAH, congenital adrenal hyperplasia; C-CAH, classic congenital adrenal hyperplasia; DHEA-S, dehydroepiandrosterone-sulphate; HDL, high-density lipoprotein; HOMA-IR, homeostatic model assessment for insulin resistance index; LDL, low-density lipoprotein; NC-CAH, nonclassic congenital adrenal hyperplasia; PCOS, polycystic ovary syndrome; SV-CAH, simple virilizing congenital adrenal hyperplasia; SW-CAH, salt-wasting congenital adrenal hyperplasia.

^*a*^Consequences for cardiometabolic health: I, increased cardiometabolic risk; N, no impact on cardiometabolic risk; D, decreased cardiometabolic risk; X, no conclusions concerning cardiometabolic risk can be drawn based on these findings.

## Blood Pressure

The relationship between BP and CAH is complex. Excessive mineralocorticoid or glucocorticoid dosages can lead to hypertension. Obesity can be associated with hypertension. Analyzing BP should be done in the context of confounding and contributing factors including BMI, glucocorticoid dose, mineralocorticoid dose, and other potential confounders.

Most studies conducted so far showed that CAH is associated with higher BP in comparison to those observed in controls (Table 5). The difference in SBP was reported more frequently than that in DBP (41, 83, 115, 120, 136, 153). Both higher SBP and DBP levels in subjects with CAH on glucocorticoid replacement therapy than in controls were also observed in a meta-analysis of 14 studies (12 longitudinal and 2 cross-sectional) (179). Elevated BP, even if the values did not meet the criteria of hypertension, were accompanied by impaired flow-mediated dilation (76), increased intima–media thickness (137), increased aortic stiffness (120) as well as structural changes in the heart (114). Moreover, it is likely that higher BP levels in CAH may impair cardiac function, leading to cardiovascular complications in later stages of life (74). In the recent retrospective cross-sectional American study, hypertension was diagnosed in 18.9% of all patients with CAH and in 57.9% of patients with concomitant cardiovascular disease. The positive association with established cardiovascular disorders was for hypertension stronger than that for other cardiovascular risk factors (59).

**Table 5.** Blood pressure in patients with congenital adrenal hyperplasia due to 21-hydroxylase deficiency

Authors	Major findings	Conclusion (consequences for cardiometabolic health)*^a^*
Abdel Meguid et al (38)	Hypertension (6.6%) less frequent vs obese controls (12.1%); correlations between SBP and DBP, and BMI and weight SDS, but not with 17OHP and glucocorticoid dose	C-CAH predisposed to hypertension in children (I)
Ahmed et al (39)	No differences in SBP and DBP vs controls; SBP and DBP higher in subjects receiving prednisone than hydrocortisone	C-CAH in children was not accompanied by changes in BP, which may be determined by glucocorticoid type (N)
Amr et al (41)	SBP higher vs controls; no differences in DBP; correlations of SBP and DBP with treatment duration but not with the daily hydrocortisone dose	C-CAH in children was accompanied by increased SBP (I)
Amr et al (42)	DBP percentiles higher vs controls; hypertension in 8.5% of patients; correlations of SBP and DBP and duration of treatment, cumulative glucocorticoid dose and mean daily glucocorticoid dose but not with glucocorticoid and mineralocorticoid doses at the time of examination	C-CAH in children predisposed to hypertension and is accompanied by higher DBP (I)
Apsan et al (43)	Lower DBP in children receiving hydrocortisone 3 times than twice daily. No between-group differences in SBP. In both groups, blood pressure values within the reference range	Association between frequency of hydrocortisone administration and DBP in children with C-CAH (X)
Arlt et al (3)	SBP lower vs population-based controls in men with C-CAH but not in women with C-CAH or NC; DBP higher vs population-based controls in women with C-CAH but not men with C-CAH and women with NC	Inconsistent results, did not allow to draw conclusion about BP in adults with CAH (X)
Auer et al (45)	24-hour DBP, daytime SBP, and daytime DBP in adults on synthetic glucocorticoids higher vs subjects receiving hydrocortisone; nondipping (dipping <10%) in 62.5% of patients receiving hydrocortisone and 29.2% of patients receiving synthetic glucocorticoids; systolic dipping in subjects on synthetic glucocorticoids more pronounced vs subjects receiving hydrocortisone; correlations between systolic and diastolic dipping with sodium (negative) and plasma renin concentration (positive) only in subjects receiving synthetic glucocorticoids	SW in adults was often accompanied by nondipping; type of glucocorticoid may determine BP in ambulatory BP monitoring (I)
Bacila et al (48)	Raised BP in 5 patients with CAH (5%) and 1 control (1%); in 4 out of 5 patients fludrocortisone dose between 60 and 200 μg/m^2^	C-CAH in children may predispose to increased BP (I)
Ben Simon et al (50)	No differences in SBP and DBP vs controls	NC-CAH in children was not accompanied by changes in BP (N)
Bonfig et al (51)	Hypertension in 12.5% of patients with C-CAH; hypertension more frequent in children than adolescents; in the age range from 12 to 18 years hypertension more prevalent in girls (12%) than boys (5.3%), in adults prevalence of hypertension not increased; SBP z-score more elevated then DBP z-score; SBP and DBP z-scores higher in SW than SV; BP correlated with age, BMI SDS, and fludrocortisone dose (≤8 years old); no correlation with hydrocortisone equivalent dose and height SDS	High prevalence of hypertension in children with C-CAH, association of hypertension with age, sex and type of C-CAH (I)
Borges et al (53)	No differences in SBP and DBP vs control men and women	C-CAH in young adults was not accompanied by changes in BP (N)
Borges et al (54)	No differences in 24-hour SBP and DBP, daytime SBP and DBP, nocturnal and DBP, pulse pressure (24-hour, daytime and nocturnal), load of SBP and DBP, and nocturnal dipping in SBP and DBP vs controls; no correlation between 24-hour, daytime and nocturnal SBP and DBP and hydrocortisone equivalent dose and serum androstenedione	C-CAH in young adults is not accompanied by changes in BP (N)
Bouvattier et al (56)	SBP and DBP lower vs healthy French men	C-CAH was associated with lower BP (D)
Charoensri and Auchus (59)	Hypertension in 18.9% of individuals with CAH no difference in the prevalence between males and females, and between CAH and NC-CAH; higher prevalence in subjects with (57.9%) than without (15.7%) established cardiovascular diseases; association with established cardiovascular disease in both univariable analysis and in the adjusted model	CAH was often accompanied by hypertension in adults (but it was difficult to say whether more frequent than in general population) (X)
de Oliveira et al (61)	No differences in SBP and DBP vs controls; SBP and DBP lower in SW than SV	C-CAH was not associated with changes in BP in young adults (N)
de Silva et al (63)	BP load >90th percentile in 5 patients (45.5%); no cases of BP load >95th percentile; mean SBP and DBP in awake (but not sleep) period higher in patients with than without BP load at 90th percentile, absent nocturnal drop of SBP and/or DBP in 7 patients (63.6%) (systolic nondippers: 63.6%, systolic and diastolic nondippers:36.4%); daytime systolic hypertension in 1 patient (9.1%); nocturnal hypertension in 6 patients (54.5%); no association with age, height, weight, BMI, and glucocorticoid dose and treatment duration	High prevalence of BP load >90th percentile, nondipping and nocturnal hypertension (I)
Delai et al (66)	No difference in SBP and DBP vs controls	It is difficult to draw conclusions because controls were older than patients by an average of 10 years (X)
Espinosa-Reyes et al (69)	No differences in SBP and DBP vs sex-, age-, and BMI-matched control subjects	CAH was not associated with changes in BP in children and young adults (N)
Falhammar et al (70)	No differences vs controls in supine and standing SBP and DBP in females. Standing DBP higher in women <30 years than ≥30 years; hypertension in 4 women with CAH (12%) ≥30 years and in no control; 3 women with CAH (9%) ≥30 years and no control were treated with antihypertensives	Relatively high prevalence of hypertension in women with CAH aged 30 years or older (but difficult to say whether more frequent than in their peers) (X)
Falhammar et al (72)	Insignificantly increased 24-hour DBP in subjects <30 years; no differences in 24-hour SBP, nighttime SBP, and nighttime DBP; increased 24-hour heart rate in the entire cohort and in men ≥30 years of age	CAH may be associated with increased 24-hour DBP in patients younger than 30 years (I)
Falhammar et al (74)	Increased prevalence of hypertension in the whole population of patients with CAH and in women with CAH but not in men with CAH	Increased prevalence of hypertension in women with CAH (I)
Falhammar et al (75)	Hypertension more prevalent vs controls	Increased prevalence of hypertension in patients with CAH (I)
Farghaly et al (76)	SBP and DBP higher vs controls; SBP and DBP higher in patients with poorly- vs well-controlled CAH; mean BP even in poorly controlled patients not abnormally high; correlations of SBP and DBP with neopterin and reverse correlations with flow-mediated dilation; no differences in heart rate	Increased prevalence of hypertension in children with C-CAH (particularly poorly controlled) (I)
Finkielstain et al (77)	Elevated BP more common in children and adults with C-CAH vs NC; no differences in BP between SW and SV; hypertensive BP correlates with suppressed plasma renin activity and younger age in children, and with male sex and 17OHP levels in adults; no correlation with BMI, obesity, and fludrocortisone dose	Type of CAH determines BP (X)
Green-Golan et al (80)	No differences in SBP, DBP, and heart rate vs controls throughout exercise and recovery	C-CAH in adolescents and young adults was not accompanied by changes in BP (N)
Harrington et al (83)	Higher SBP vs controls; no differences in DBP; no association between SBP and DBP and renin levels	C-CAH was accompanied by increased SBP in children (I)
Hashemi Dehkordi et al (84)	Positive correlation between DBP and 17OHP	DBP in children may be increased if C-CAH was poorly controlled (I)
Hoepffner et al (85)	Absence of nocturnal dip in 28% of in-patient measurements and in 27% 24-hour ambulatory blood pressure measurements; SBP and DBP on admission higher than in outpatient clinics in children/adolescents but not in adults; outpatient SBP >95th percentile: 13% of children/adolescents; white-coat hypertension: 9% of children/adolescents; DBP >95th percentile: 0% of children/adolescents; average BP in adults in the upper normal range; no correlation with sex, BMI, and mineralocorticoid dose	C-CAH predisposed to systolic hypertension, white-coat hypertension and nondipping, as well as higher admission than office BP in children (I)
Janus et al (86)	No cases of overt hypertension; 24-hour SBP loads and daytime SBP loads higher in Del/Del vs other subgroups; nighttime SBP load higher in Del/Del and Del/I2 spice vs other genotypes; 24-hour SBP loads, daytime SBP loads and nighttime SBP loads lowest in NC and I2G/I2G subgroup. 24-hour DBP, daytime DBP loads and nighttime DBP loads higher in SV and lower in I2G /I2G, Del/I2G and NC vs other groups; abnormal nighttime dip in 57% of patients (highest in I2G /I2G); 24-hour, daytime and nighttime SBP loads higher in patients with advanced bone age and higher in females than males with advanced bone age; higher night DBP load in females with advanced bone age; no differences in 24-hour DBP loads and day DBP loads between the groups; night dipping greater in affected females with than without advanced bone age	24-hour ambulatory BP measurements in children with CAH depended on phenotype and genotype (X)
Jenkins-Jones et al (87)	No differences in SBP in males and females vs controls; no data on DBP	Uncomplete data concerning BP but suggested unaltered SBP in CAH (X)
Kara et al (88)	No differences in SBP and DBP vs BMI-, age-, and gender-matched controls; no differences in SBP and DBP between patients receiving hydrocortisone and dexamethasone	C-CAH was not associated with changes in BP (N)
Kim et al (90)	<18 years: hypertension: 14%, prehypertension: 11%; all 4 patients ≥18 years had normal BP; no difference in fludrocortisone dose between normotensive and hypertensive patients	High prevalence of hypertension and prehypertension in children with C-CAH {I)
Kim et al (91)	No differences in SBP and DBP vs controls	C-CAH was not associated with changes in BP in children and young adults (N)
Korkmaz et al (93)	SBP higher vs controls; no differences in DBP; no correlations with hydrocortisone dose; no differences in SBP and DBP between fludrocortisone-treated and fludrocortisone-naïve patients; correlations of SBP with weight, height, BMI, bone age, treatment duration, total cholesterol, LDL cholesterol, insulin, and HOMA1-IR; correlations of DBP with weight, height, BMI, bone age, and treatment duration	CAH was associated with increased SBP in children (N)
Kroese et al (94)	Lower values of 24-hour, daytime and nighttime SBP, DBP, and mean arterial pressure, as well as 24-hour and nighttime heart rate in pioglitazone- than placebo-treated patients with CAH; no differences between the groups in nocturnal drop of SBP and DBP	Pioglitazone treatment was associated with lower ambulatory 24-hour BP (X)
Kurnaz et al (99)	No differences in SBP and DBP vs controls	C-CAH was not associated with changes in BP in children and young adults (N)
Liivak and Tillmann (100)	Mean SBP SDS and DBP SDS below normal population; 24-hour, daytime and nighttime SBP SDS and 24-hour DBP SDS higher if higher hydrocortisone dose was taken in the evening than in the morning; mean drop in nighttime SBP: 8.8% (higher hydrocortisone dose in the morning) and 8.0% (higher hydrocortisone dose in evening)	Conclusions concerning SW in children unclear because of inconsistent results (X)
Lim et al (101)	SBP and DBP in men and women with CAH higher vs respective controls; increased risk of hypertension in men with CAH	C-CAH was accompanied by higher SBP and DBP in young adults, and predisposed to hypertension in men (I)
Maccabee-Ryaboy et al (103)	Rate of hypertension higher in fludrocortisone-treated (55%) vs fludrocortisone-naive (31%) children (observed in both males and females); rate of hypertension higher in SW (58%) vs SV (35%); SW: hypertension cases—60% (males) and 55% (females), hypertension before age of 5: 91% (males) and 50% (females), more rapid decline with age in males than females; SV: 21% (males) and 48% (females), most cases in both sexes aged 5 years or older; incidence rate above 50% through aged 10-18 for both SW and SV; higher risk of hypertension in subjects with oversuppression, especially treated with fludrocortisone; no association of hypertension with glucocorticoid dose	High prevalence of hypertension in children with C-CAH, more pronounced in SW than SV and in treated with fludrocortisone, and changing with age (I)
Marra et al (104)	No differences in resting SBP vs controls; a trend to higher DBP vs controls; higher peak SBP and ΔSBP in response to exercise in both males and females; no differences in peak DBP and ΔDBP; correlations between SBP and ΔSBP and HOMA-IR; no differences in heart rate	C-CAH in children was associated with higher exercise-induced SBP and may be also with resting DBP (I)
Metwalley et al (105)	No differences in SBP and DBP vs controls	C-CAH was not associated with changes in BP in children (N)
Metwalley et al (107)	SBP and DBP higher vs control subjects; blood pressure in all children within normal range; correlations between SBP and DBP and epicardial fat thickness	C-CAH was not associated with increased SBP and DBP in children (I)
Mnif et al (109, 110)	No differences in 24-hour, daytime and nighttime SBP and DBP between patients with SW, SV, and NC; daytime systolic hypertension in 1 patient (4%); nocturnal dipping absent in 19.2% of patients	CAH was accompanied by increased risk of nondipping (I)
Mooij et al (111)	Rapidly changing BP in the first 8 weeks of life; mean peak SBP within the reference range except for females aged 9-16 weeks; positive correlation of peak BP with renin concentration in weeks 0-2; negative correlation between peak SBP and renin concentration in weeks 25-32; no correlation of BP with 17OHP, androstenedione, and fludrocortisone dose	Because of inclusion criteria, results did not allow to conclude about long-term consequences (X)
Mooij et al (112)	No difference in supine and upright office SBP, DBP, and heart rate vs controls; mean 24-hour SBP, DBP, mean BP, and heart rate higher vs controls; daytime and nighttime DBP but not SBP higher vs controls; no difference vs controls in nighttime BP dip	CAH in adults was associated with higher 24-hour BP (but not office BP) (I)
Mooij et al (113)	Patients with systolic hypertension: 18.5%; patients with prehypertensive values of SBP: 14.8%; patients with prehypertensive values of DBP: 11.1%; dip of SBP <10% during sleep: 48.1% of patients; dip of mean blood pressure <10% during sleep: 40.7% of patients; mean sleeping SBP SDS, DBP SDS, and mean pressure SDS higher vs normal ranges for age and height; negative correlation between 24-hour and sleep DBP SDS to height and androstenedione, negative correlations of daytime DBP SDS and HOMA-IR; negative correlation of mean BP dip during sleep with BMI SDS; no association of other BP measurements with hydrocortisone and fludrocortisone doses, therapy control, HOMA-IR and body composition	CAH in children predisposed to systolic hypertension, prehypertension in children, and nondipping (I)
Moreira et al (115)	Higher SBP and insignificantly higher DBP in CAH males vs CAH females; correlations between SBP and DBP and BMI; SBP higher in *Bcl*I polymorphism carriers; hypertension in 12% of patients, more prevalent in SV vs SW	C-CAH in some adult patients may be complicated by hypertension (but unclear whether more often than in the general population); SBP may differ between affected males and females (X)
Moreira et al (116)	No difference in the percentage of subjects with BP >90th percentile between girls and boys and between SW and SV; BP >90th percentile more frequent in obese than nonobese subjects with CAH; correlations between SBP and BMI; no correlation between BP and fludrocortisone dose in subjects with SW	Prevalence of BP >90th percentile in children with C-CAH may depend on weight status (X)
Navardauskaite et al (117)	Elevated BP more often in patients with CAH (34%) vs controls (12.5%); elevated BP in 45% of patients with SW and in 16.6% of patients with SV; no difference between patients with elevated and normal BP in age, indices of adiposity, glucocorticoid and mineralocorticoid doses, insulin sensitivity and hormone levels; no correlation between SBP or DBP and glucocorticoid and mineralocorticoid doses	C-CAH (more often SW) predisposed young adults to elevated BP (I)
Nebesio and Eugster (118)	BP >95th percentile in 6.6% of patients (in 5.5%: essential hypertension, in 1.1%: hypertension secondary to rhabdomyolysis and acute renal failure complicating CAH-induced adrenal crisis and shock)	High prevalence of hypertension in children with CAH (I)
Nermoen et al (119)	DBP higher vs controls in both males and females; no differences in SBP; DBP higher in SV vs SW; positive correlation between DBP and aldosterone concentration; no correlation between SBP and DBP and BMI, fat mass, glucocorticoid dose, and genotype	C-CAH in adults was often accompanied by increased DBP (more pronounced in case of SV) (I)
Neumann et al (25)	18 months of life—SBP >95th percentile: 57%, DBP >95th percentile: 75%; 30 months of life—SBP >95th percentile: 35%, DBP >95th percentile: 52%; no differences in SBP and DBP between children receiving and not receiving salt supplementation; 1 year of age: positive correlations of BP with total hydrocortisone dose (SBP), fludrocortisone dose (SBP), total glucocorticoid action (SBP) and total mineralocorticoid action (SBP and DBP)	SW in small children was often complicated by hypertension (I)
Özdemir et al (120)	SBP higher vs controls; no differences in DBP	C-CAH in children was accompanied by increased SBP (I)
Paizoni et al (121)	24-hour and daytime SBP and DBP within normal limits; absence of nocturnal BP dip in most patients; no differences in prevalence of nondippers between SW and SV; no impact of evening intake of glucocorticoids and fludrocortisone on nocturnal BP dip	C-CAH in most adults was complicated by nondipping (I)
Roche et al (127)	No differences in SBP and DBP between males and females; negative relationship between SBP and age in males; DBP does not change with age; systolic hypertension in 58% of patients (males: 67%, females: 52%); high normal SBP in 10% of patients; diastolic hypertension in 24% of patients (males: 13%, females: 37%); high normal DBP in 8% of patients; combined systolic and diastolic hypertension in 21% of patients (no differences between both sexes); impaired nocturnal dip in SBP and DBP in 84% and 29% of patients; absent loss of both systolic and diastolic nocturnal dip in 28% of patients (20% of males and 35% of females); correlations between BP and BMI; no correlations with plasma renin activity, androstenedione, and 17OHP	SW in children and adolescents predisposed to systolic and diastolic hypertension, prehypertension and nondipping (I)
Rodrigues et al (128)	More patients under 18 years old with SBP ≥90th percentile; no difference in the percentage of patients with DBP ≥90th percentile under 18 years old; no differences in SBP and DBP between SW and SV	C-CAH in children was associated with higher of SBP ≥90th percentile (I)
Rosenbaum et al (129)	SBP, DBP, central SBP and central DBP higher vs controls; no difference in pulse pressure; unclear information on heart rate	CAH was accompanied by increased SBP and DBP (I)
Sartorato et al (130)	No differences in SBP and DBP vs controls in males and females	C-CAH in young adults was not accompanied by changes in BP (N)
Schröder et al (132)	No differences in mean overnight DBP and SBP between 2 treatment strategies (the highest hydrocortisone dose in the morning and in the evening) in patients with at least 5 nocturnal measurements	No association between hydrocortisone treatment regimen and BP in children and young adults (X)
Subbarayan et al (136)	SBP and SBP SDS higher vs reference values; DBP and DBP SDS insignificantly higher vs reference values; systolic hypertension in 20% of patients; diastolic hypertension in 9% of patients; systolic and diastolic hypertension in 3% of patients, prehypertension in 11% of patients; no differences in prevalence of systolic and diastolic hypertension between both sexes and between SW and SV; negative correlations of SBP SDS with age; correlations of DBP SDS with cortisol levels and fludrocortisone dose	C-CAH in children and young adults was accompanied by increased SBP, and predisposed to hypertension and prehypertension (I)
Tony Nengom et al (137)	SBP and DBP higher vs controls; no correlation between SBP and DBP and hydrocortisone dose; correlations between SBP and DBP and intima–media thickness; no differences in SBP and DBP between fludrocortisone-treated and fludrocortisone-naïve patients	CAH in children was accompanied by increased SBP and DBP (I)
Torky et al (138)	Mean age of hypertension 5.7 years; hypertension in 93.0% of patients, most commonly <2 years, then its prevalence declines throughout childhood before increasing in adulthood; hypertension more frequent in men (39.5%) than women (25.4%); hypertensive BP independently associated with obesity and mineralocorticoid dose (especially in children); increased likelihood of hypertensive blood pressure associated with suppressed plasma renin activity, suppressed androstenedione levels, low 17OHP (in childhood), obesity, and fludrocortisone dose; no association with glucocorticoid type, dose and nighttime dose, and height SDS; advancing age lowers the risk of hypertension	C-CAH predisposes to hypertension, which was more frequent in males and its prevalence depends on age (I)
Tuhan et al (139)	No differences in SBP, SBP SDS, DBP, and DBP SDS vs controls; insignificantly higher heart rate in patients with CAH	C-CAH was not associated with changes in BP in children (N)
Tuhan et al (140)	No differences in SBP, SBP SDS, DBP, and DBP SDS vs controls; no cases of hypertension in the studied patients; no differences in SBP SDS and DBP SDS between SW and SV	C-CAH was not associated with changes in BP in children (N)
Ubertini et al (141)	No differences in SBP and DBP in response to exercise vs reference range; no differences in mean diurnal and nocturnal SBP and DBP vs reference range; no cases of hypertension in subjects with CAH; negative correlations between mean diurnal SBP and 17OHP; positive correlations between mean diurnal DBP and age and height SDS; positive correlations between mean nocturnal DBP and testosterone; positive correlations between SBP at maximal heart rate and age; positive correlations between DBP at maximal heart rate and age and BMI SDS	C-CAH in children and adolescents was not accompanied by changes in BP (N)
Vijaran et al (142)	DBP higher vs controls; no differences in SBP; most (87%) children with CAH normotensive, stage 1 hypertension in 5 patients (9%), prehypertension in 2 patients (4%); correlations of SBP and DBP with HOMA1-IR and BMI but not with hydrocortisone and fludrocortisone doses	C-CAH in children and young adults was associated with increased DBP, and relatively often predisposes to hypertension and prehypertension (I)
Völkl et al (143)	SBP SDS elevated in girls but not boys; DBP SDS decreased in boys and normal in girls; higher daytime (in 11% of patients >97th percentile) and nighttime (in 13% of patients >97th percentile) SBP SDS; lower daytime DBP SDS; normal nighttime DBP SDS; normal nocturnal drop of SBP SDS; decreased nocturnal drop of DBP SDS; no differences between SW and SV in 24-hour ambulatory blood pressure measurements; correlations between SBP SDS and DBP SDS and BMI and skinfold thickness; no correlation with hydrocortisone equivalent and fludrocortisone dose; highly pathological BP profile in 1 patient (2%)	C-CAH was accompanied by increased SBP in children and young adults (more pronounced in females) (I)
Wasniewska et al (147)	SBP SDS in patients with C-CAH higher vs controls; no differences in SBP SDS vs controls in patients with NC; lower DBP SDS vs controls in patients with C-CAH and NC; no differences in SBP SDS and DBP SDS between patients with C-CAH and NC; SBP above the highest limit of normal range in 2 patients with C-CAH (22%) and 2 patients with NC (22%)	High prevalence of systolic hypertension in adolescents and young adults with C-CAH and NC-CAH (I)
Wierzbicka-Chmiel et al (150)	No differences vs controls in SBP and DBP	SW was not associated with changes in BP in young adults (N)
Williams et al (151)	SBP higher vs controls in NC but not in C-CAH; no data on DBP	NC-CAH was associated with increased SBP om children (I)
Zhang et al (153)	SBP higher, DBP insignificantly higher vs controls	Untreated SV was accompanied by increased SBP in girls and young adult women (I)

Summary: Unfavorable impact of CAH on blood pressure in 40 studies (56.3%), no impact of CAH in 16 studies (22.5%), favorable impact of CAH in 1 study (1.4%), while 14 studies (19.7%) were inconclusive.

Abbreviations: 17OHP, 17-hydroxyprogesterone; BMI, body mass index; BP, blood pressure; CAH, congenital adrenal hyperplasia; C-CAH, classic congenital adrenal hyperplasia; DBP, diastolic blood pressure; HOMA-IR, homeostatic model assessment for insulin resistance index; LDL, low-density lipoprotein; NC, nonclassic phenotype; SBP, systolic blood pressure; SDS, standard deviation score; SV, simple virilizing phenotype; SW, salt-wasting phenotype.

^a^Consequences for cardiometabolic health: I, increased cardiometabolic risk; N, no impact on cardiometabolic risk; D, decreased cardiometabolic risk; X– no conclusions concerning cardiometabolic risk can be drawn based on these findings.

The risk of hypertension seems to be greater in children and maybe also in adolescents than in young adults. In the study by Finkielstain et al, hypertension, defined as SBP or DBP in the 95th percentile or greater for age, sex, and height, was reported in two-thirds of children with SW-CAH which was much more frequent than in the age-matched general population (3%) (77). Bonfig et al observed that hypertension occurred more frequently before the age of 12 years than in patients with CAH aged between 12 and 18 years (51). Moreover, in adults with CAH (aged 18 years or older), the prevalence of hypertension did not differ from the general population. In a study by Kim et al, 14% of patients younger than 18 years old were hypertensive and 11% were prehypertensive, while BP of the 4 included young adults were within the reference range (90). Maccabee-Ryaboy et al observed that the risk of hypertension in children, particularly boys, with SW-CAH was highest below the age of 5 with a subsequent decline, more pronounced in males than females (103). However, most cases of hypertension in SV-CAH were found in both boys and girls aged 5 years or older. Torky et al reported the highest prevalence of hypertension in children younger than 2 years (138). The prevalence of hypertension, defined as BP above 95th percentile for age and gender, was found to be greater (6.6%) than the historically reported prevalence in children (1%) and greater than the prevalence in obese/overweight pediatric population (4.5%) (118). In glucocorticoid-treated North African children with CAH aged 4-12 years, the prevalence of hypertension was estimated at 8.5% (42). In a recent retrospective multicenter analysis, high SBP and DBP levels were reported most frequently (57% and 75%) at month 18, and then gradually decreased to 35% and 52% at month 30 (25). The increased risk in the early childhood may be attributed to relatively high doses of mineralocorticoids used in the replacement therapy and/or to additional salt supplementation, aimed at compensating the physiological state of mineralocorticoid resistance and increased renal salt-wasting in this age group (24, 199). Therefore, the age-related decrease in fludrocortisone dosing per body weight and surface area and/or cessation of salt supplementation contribute to a reduction in BP in later periods of childhood and in young adulthood (136). However, increased prevalence of hypertension was also reported in adult patients with CAH participating in the pan-European dsd-LIFE study (75). Moreover, although there are no data concerning antihypertensives in children and adolescents with CAH, the use of antihypertensive agents in adult patients with CAH has been estimated at 7% to 9% (70, 163). Lastly, the risk of hypertension in the group of 254 adult patients with CAH (18-70 years old) correlated with age (59). All these observations suggest the likely role of aging-related mechanisms in the development of this complication at later stages of life.

Another characteristic feature of CAH is an increased prevalence of nondipping phenomenon (45, 113, 121, 127, 143), defined as the loss of the usual physiologic nocturnal drop in BP, which is a well-known cardiovascular risk factor (200). The percentage of nondippers in CAH ranged from 19.2% in the study by Mnif et al (110) to as much as 84% in the study by Roche et al (127). The lack of nocturnal drop in BP was not limited to patients with hypertension and was observed even in patients in whom BP was within the reference range (121).

The data concerning the association between the risk of hypertension in CAH and sex are inconclusive. Most studies have not reported differences in the incidence of hypertension between males and females (77, 85, 127, 130, 136, 141, 143). Some authors observed, however, that females with CAH were more prone to hypertension than men, explaining their findings by more pronounced hypertensive effects of androgen excess in females (51, 74, 86). Finally, there are few studies suggesting a male predominance of hypertension in this disorder (77, 101, 115).

Another controversial question is the relationship between BP and CAH phenotype/genotype. In the majority of studies, BP was similar in SW-CAH and SV-CAH (77, 110, 116, 143). Moreover, Wasniewska et al observed no differences in SBP SDS and DBP SDS between patients with C-CAH and NC-CAH, despite glucocorticoid doses being higher in the classic form (147). However, Falhammar et al observed that only females with SV-CAH and to a lesser extent also females with NC-CAH (but not patients with SW-CAH) had increased risk of hypertension (74). This may be associated with more pronounced obesity in the milder forms of CAH, and maybe also with possible fludrocortisone usage in patients with non–SW-CAH. Furthermore, 3 other studies reported higher BP in SV-CAH than SW-CAH (61, 115, 119). Moreover, Williams et al reported higher SBP levels in NC-CAH than in C-CAH (151), while the opposite findings were found by Finkielstain et al (77). In a recent study by Righi et al, the number of patients with SW-CAH and SV-CAH receiving antihypertensives was the same (163). However, considering that the former is more prevalent than the latter, this finding may suggest that more individuals with SV-CAH need to be treated with antihypertensive agents. In other studies, hypertension and higher BP levels were observed more frequently in children and adults with SW-CAH than with SV-CAH (51, 103, 117). It is difficult to explain the reasons for these inconsistencies. They may result from differences in baseline characteristics of studied populations (ethnicity, age, sex proportion, age at diagnosis, treatment duration, and glucocorticoid and mineralocorticoid dose).

Some pieces of evidence appear to suggest that increased BP levels may depend on metabolic control rather than on the presence of CAH per se. Both SBP and DBP were higher in children and adolescents with poorly controlled CAH than in well-controlled CAH (76). In children and adolescents, DBP was found positively correlated with 17OHP concentrations (84). Moreover, higher BP in children and adolescents with CAH correlated with advanced skeletal maturation (86, 93), which is a marker of poor metabolic control for a longer period of time (201). In glucocorticoid-treated patients with CAH, exercise induced an excessive increase in SBP compared with controls (104). However, the increase in SBP in response to exercise was normal in individuals with good hormonal balance (141). Children and adolescents with CAH had elevated daytime and nighttime SBP, but they were more obese than controls and their BP correlated with BMI and skinfold thickness (143). The association between BP and BMI was observed also by others (38, 93, 116, 127). Only in the study by Nebesio and Eugster the risk of increased body weight did not differ statistically between children with both CAH and hypertension and nonhypertensive children with CAH (118). Moreover, in a recent study hypertension was diagnosed almost twice as frequently in obese children than in patients with CAH, 40% of whom had normal weight (38). Lastly, in a Swedish population-based cohort study, including 1305 patients with primary AI, the participants were prescribed more antihypertensive drugs than controls (202). This finding, which is in contrast to the commonly accepted view that low BP and hypovolemia are an almost invariable feature of untreated or undertreated Addison disease (203), suggests that mechanisms underlying increased BP in CAH are at least partially associated with treatment.

There are many arguments supporting the association of elevated BP with glucocorticoid replacement therapy. Falhammar at al observed higher SBP and DBP in milder (I172N) than more severe genotypes (the null and I2G genotypes) (72). This finding may be well explained by using similar doses of glucocorticoids in subjects independent of genotype. It is likely that, because of a milder enzymatic defect, these doses might have been too high for individuals with I172N genotype, resulting in overtreatment. Consecutively, BP was similar to that in controls if patients with NC-CAH received glucocorticoids in small doses (50, 66). Charoensri and Auchus reported that the risk of hypertension was associated with taking higher doses of glucocorticoids (but not of fludrocortisone) (59). The association with supraphysiological glucocorticoid replacement was also supported by correlations between BP and glucocorticoid dose and duration (25, 41, 93, 136), as well as by higher rates of hypertension in individuals with supraphysiological glucocorticoid replacement (103). Moreover, BP differed between patients with CAH receiving different doses of hydrocortisone (103). The prevalence of hypertension was higher in the study of children with higher hydrocortisone doses (17.5 ± 2.1 mg/m^2^) (127) than in the study including children treated with lower doses (13.3 ± 4.4 mg/m^2^) (136). Moreover, compared with noncarriers, adult carriers of the *Bcl*I polymorphism had higher SBP levels (115), suggesting the role of glucocorticoid receptor activity in regulation of BP in patients with CAH. Finally, high glucocorticoid levels saturating 11β-hydroxysteroid dehydrogenase type 2 impair conversion of cortisol to cortisone, the consequence of which is a stimulation of the mineralocorticoid receptor by cortisol (204). Various doses of glucocorticoids may result in various degrees of activation of the mineralocorticoid receptor and may be associated with changes in the setpoint for activity of 11β-hydroxysteroid dehydrogenase (mainly type 2). However, the association between glucocorticoid replacement and an increase in BP is not so clear-cut. Some researchers did not find correlations between CAH and glucocorticoid dose in patients with CAH (54, 117, 137, 142, 143). In turn, Williams et al reported higher SBP only in children with NC-CAH but not in C-CAH, although children with NC-CAH were treated with only small glucocorticoid doses (151). The possible explanation for these inconsistent results is that the impact of high-dose glucocorticoid therapy on BP is partially counterbalanced by a decrease in activity of the endogenous renin–angiotensin–aldosterone system and by decreased sympathetic activity, and the net effect varies depending on the population's characteristics.

The impact of glucocorticoids on blood pressure (BP) may be also determined by the glucocorticoid regimen. Children with CAH demonstrated higher 24-hour SBP and DBP, as well as daytime and nighttime SBP if a higher hydrocortisone dose was taken in the evening than in the morning, although the daily dose was the same in both treatment regiments (100). Moreover, Apsan et al reported lower DBP in children with CAH receiving hydrocortisone 3 times than twice daily (43). In contrast, Schröder et al did not observe differences in mean overnight SBP and DBP between children and adolescents receiving the highest hydrocortisone dose in the morning or in the evening (132). Another potential explanation is that the impact on BP partially depends on the type of glucocorticoid. Children on prednisone treatment had slightly higher SBP and DBP levels than their peers treated with hydrocortisone (39). Moreover, Auer et al have recently reported higher 24-hour DBP, daytime SBP and daytime DBP levels and more pronounced nocturnal BP dipping in adults receiving synthetic glucocorticoids than hydrocortisone (45). Although in a recent study BP did not differ between patients treated with hydrocortisone and dexamethasone (88), but this may result from matching patients and controls for BMI.

Increased BP may be also attributed to using high daily doses of fludrocortisone or unjustified treatment with mineralocorticoids in individuals with SV-CAH and suppressed renin levels/activity. In line with this explanation, the rate of hypertension was much higher in fludrocortisone-treated (55%) than fludrocortisone-naïve (31%) children (103). In children and adolescents with CAH receiving low doses of fludrocortisone (48 μg/m^2^ daily), daytime and nighttime systolic hypertension was observed only in 11% and 13%, respectively, while DBP was either normal (in obese subjects) or reduced (in patients with normal weight) (143). In a recent study by Bacila et al, 4 out of 5 children and adolescents with CAH and raised BP had a daily fludrocortisone dose ranging between 60 and 200 μg/m^2^ (48). BP was found to correlate with fludrocortisone dose in many studies (25, 51, 136, 138) and mineralocorticoid action in 1 (25). Supporters of this explanation suggest a cautious approach to prescribing fludrocortisone and are in favor of limiting daily dosage of this agent to 100 μg (136). However, there are arguments against this. Most studies did not report differences in BP between subjects with SW-CAH and SV-CAH (77, 110, 116, 140, 143), although only in the SW phenotype mineralocorticoids are compulsory (2, 14, 22, 205). Korkmaz et al observed no differences in SBP and DBP in fludrocortisone-treated and fludrocortisone-naïve children with CAH (93). Kim at al reported no differences in fludrocortisone dose between hypertensive and normotensive subjects with CAH (90). Williams et al observed higher values of SBP in children with NC-CAH than with C-CAH, although only 2 patients with NC-CAH received fludrocortisone (151). Moreover, there was no correlation between BP and fludrocortisone dose in several studies (85, 116, 117, 142, 143). These contradictory results may be explained by the fact that 21OHD is characterized by elevated levels of steroid precursors, either with antimineralocorticoid properties (17-hydroxyprogesterone and progesterone), or which are partial agonists of the mineralocorticoid receptor (21-deoxycorticosterone and 21-deoxycortisol) (206, 207). These precursors, the concentrations of which differ between various study populations, may affect baseline mineralocorticoid activity and fludrocortisone action.

The role of androgen excess in the development of hypertension in patients with CAH is supported by correlations between androgen concentrations and SBP and DBP (113, 141). Moreover, testosterone concentrations were found to correlate with mean nocturnal DBP levels, suggesting that higher nocturnal DBP levels represents an early effect of androgen excess in CAH (141). High androgen concentrations may exert hypertensive properties, increasing vascular reactivity and impairing endothelial-dependent relaxations, and these effects seem to be stronger in women than men (208). This mechanism may also partially explain sex differences in the prevalence of hypertension in subjects with CAH. However, the lack of such correlations reported by Roche et al is in disagreement with this interpretation (127).

Interestingly, some studies revealed low BP in patients with CAH. SBP and DBP were decreased in a cohort study of adult UK patients with CAH (3), as well as in the study by Bouvattier et al including adult French men with C-CAH (56). Moreover, Völkl et al observed that, unlike obese children with CAH having normal DBP, DBP of children with CAH and normal weight was lower than in controls (143). There are at least 3 possible explanations for these findings. Firstly, low BP may result from mineralocorticoid and glucocorticoid deficiency and/or may be secondary to insufficient re-uptake of sodium as well as weakening of direct aldosterone action on the heart and blood vessels as a result of failure of the mineralocorticoid receptor. The role of chronic glucocorticoid and mineralocorticoid undertreatment is supported by markedly increased levels of ACTH and active renin in most participants of the study by Bouvattier et al (56). Moreover, elevated 17OHP concentrations, suggesting glucocorticoid undertreatment, were found associated with lower mean diurnal SBP (141). Secondly, low BP may be a manifestation of adrenomedullary failure, reflected by low plasma/urine epinephrine and metanephrine concentrations in CAH (72, 193, 209). Finally, low pressure may be secondary to an unjustified restriction of salt intake by many patients with C-CAH on glucocorticoid and mineralocorticoid replacement therapy.

Studies examining BP in individuals with 21OHD have numerous methodological limitations, which may contribute to their different results. Depending on study design, BP was measured in supine or upright positions (sometimes only a single random measurement) or by 24-hour ambulatory measurement. The 24-hour ambulatory BP monitoring is considered a better indicator of BP than random assessment. Its use eliminates observer bias, excludes subjects with “white coat” and “isolated office” hypertension, as well as more closely correlates with target organ damage than office-based BP (210). A limiting factor of children's studies might be the clinical setting of measurements. Twenty-four-hour BP monitoring may provide falsely elevated results, if conducted during hospitalization which is sometimes done to monitoring treatment effectiveness (85). The oscillometric method used in some studies may underestimate BP by a few mmHg compared with sphygmomanometric method (211). Certainly, it cannot be excluded that the increased prevalence of hypertension in younger children with CAH results from difficulties in BP measurements. In children and adolescents, references for BP are corrected either for chronological age or for height. Considering the impact of CAH on growth velocity, SDS calculated for height may provide incorrect results. Differences in the obtained results may also be attributed to differences in therapeutic aims. The primary aim is either to normalize BP and sodium concentrations, or to reach renin activity/concentration in the upper half of the reference range. Moreover, laboratory parameters assess metabolic control of CAH during the last hours or days (2), but do not allow to effectively monitor the safety of glucocorticoid and mineralocorticoid treatment over a period of months or years (212). Finally, in some studies, we cannot exclude the impact of a possible occurrence of other than 21OHD genetic defects underlying CAH (213).

Describing the association between 21OHD and BP, it should be kept in mind that elevated BP is a characteristic feature of 11β-hydroxylase deficiency and 17α-hydroxylase/17,20-lyase deficiency due to differences in the steroid precursor load (6, 8). In 11β-hydroxylase deficiency, at the time of diagnosis, hypertension is observed in two-thirds of patients, and the risk seems to increase with age (6). In the largest clinical study so far in 11β-hydroxylase deficiency, 102 children, adolescents, and young adults were examined (213). Around half of those with classic 11β-hydroxylase deficiency received regular at least 1 antihypertensive drug while none of the 10 patients with NC phenotype did so. Interestingly, 19% had been misdiagnosed as 21OHD initially. In 2 large series of patients with 17α-hydroxylase/17,20-lyase deficiency, the incidence of hypertension was found in 88.9% (214) and 95.6% (215), and 2 cases were inappropriately diagnosed with primary aldosteronism (216). Thus, in both 11β-hydroxylase deficiency and 17α-hydroxylase/17,20-lyase deficiency, the risk of hypertension is higher and patients present with more severe forms of hypertension than in 21OHD.

In summary, patients with CAH seem to have higher BP levels than controls and are more prone to nondipping. This risk may be more pronounced in the developmental age and in the late adulthood, but there are no convincing data on its association with sex and CAH phenotype/genotype. Increased BP may be secondary to poor metabolic control of CAH, using too high doses of glucocorticoids and/or mineralocorticoids, nonphysiological timing of their administration, treatment with synthetic glucocorticoids, and/or to androgen excess. In some patients, however, under-replacement, secondary to adrenomedullary failure and/or restricted salt intake, may result in low BP levels.

## Endothelial Function

The endothelium is an active inner layer of blood vessels, exerting many vasoprotective effects, including vasodilation, suppression of smooth muscle cell growth, and inhibition of inflammatory responses (217). Endothelial dysfunction, defined as an imbalance between relaxing and contractile endothelial factors, plays a central role in the pathogenesis of atherosclerosis and is observed already in early steps of its development (218).

A few studies investigating endothelial function in individuals with CAH provided generally consistent results. In these studies, endothelial function was quantified by flow-mediated dilation, a noninvasive, ultrasound-based technique assessing an endothelium-dependent relaxation of conduit arteries in response to shear stress and nitric oxide production (219). Farghaly et al observed lower brachial artery flow-mediated dilation in adolescents with CAH than in controls, and endothelial dysfunction correlated with serum levels of neopterin, a cellular immune system activation marker synthesized by monocyte-derived macrophages (76). The authors also showed reduced brachial artery flow-mediated dilation in adolescents with poorly controlled CAH compared with adequately controlled adolescents (76). Wierzbicka-Chmiel et al reported decreased mean flow-mediated dilation in 19 patients with CAH (mean age 23.7 years) than in controls, and the difference persisted after considering potential confounders: age, sex, BMI, smoking status, cholesterol levels, brachial artery diameter, and doses of glucocorticoids and fludrocortisone (150). Harrington et al reported reduced flow-mediated dilation and glyceryl trinitrate dilation in 14 nonobese adolescent boys with CAH (83). The reduction in vascular function was similar to that observed in obese boys with normal adrenal function, who had much higher BMI, waist circumference, the waist to hip ratio, and the waist to height ratio than individuals with CAH. Finally, compared with matched controls, 32 children and adolescents with CAH had, regardless of sex, an increased number of circulating endothelial cells, suggesting a high degree of endothelial cell injury (105). Only in 1 study did flow-mediated dilation not differ from that in the control group, which may have been a consequence of the small sample size, wide age range, underrepresentation of males, matching the groups for BMI and Tanner stage, and satisfactory disease control in most patients (69).

There are no data supporting the association of endothelial dysfunction with glucocorticoid dose and type, or with treatment duration. There are also no data on the association of endothelial dysfunction with elevated BMI and impaired insulin sensitivity. However, there are positive correlations between the indices of endothelial dysfunction and testosterone concentrations in CAH (76, 105). Thus, endothelial dysfunction may be related to glucocorticoid undertreatment but does not seem to be associated with overtreatment. Glucocorticoids regulate vascular reactivity by acting on endothelial glucocorticoid receptors, and this effect, mediated by suppressing Wnt signaling, is observed at physiological concentrations (220). Moreover, high androgen concentrations, both in males and females, has been found to negatively affect proper functioning of endothelial cells (221, 222). The early onset of unfavorable endothelial changes may be an argument against the use of too low doses of glucocorticoids in children and adolescents with CAH. However, this interesting and clinically relevant question requires better understanding.

## Intima–Media Thickness

Intima–media thickening is a feature of arterial wall aging, related to subclinical atherosclerosis (223). Its noninvasive measurement using B-mode ultrasound is a widely used surrogate marker for atherosclerotic changes in the vascular wall. Increased intima–media thickness is a predictor of clinically detectable atherosclerosis and is a well-established marker for future cardiovascular mortality and cardiovascular events, including myocardial infarction and stroke, independent of other risk factors (224, 225).

The majority of studies conducted so far have shown that CAH was associated with increased carotid artery intima–media thickness compared with controls (38-41, 105-107, 110, 120, 128, 130, 147) (Table 6). Because differences compared with controls were observed also in children, adolescents and young adults (38, 130, 147), it is likely that anatomical changes in the innermost 2 layers of the arterial wall may begin already in the early childhood. However, some studies have not shown differences in intimal–medial thickness between patients with CAH and controls (53, 66, 69, 76, 83, 91, 121, 129, 140). Moreover, carotid artery intima–media thickness did not differ between children and young adults with SW-CAH and SV-CAH (41, 91), between adolescent boys and girls with C-CAH and NC-CAH (147).

**Table 6.** Carotid intima–media thickness in patients with congenital adrenal hyperplasia

Authors	Major findings	Conclusion (consequences for cardiometabolic health)*^a^*
Abdel Meguid et al (38)	Thickness increased vs controls; correlation with BMI and weight SDS	C-CAH was accompanied by early atherosclerotic changes in the vascular wall in children (I)
Ahmed et al (39)	Thickness increased vs controls; no difference between subjects treated with hydrocortisone and prednisone; correlation with BMI SDS but not with age, daily glucocorticoid dose, lipids, insulin, HOMA, and 17OHP concentrations	C-CAH was accompanied by early atherosclerotic changes in the vascular wall in children (I)
Akyürek et al (40)	Thickness increased vs controls; correlation with impaired nocturnal DBP dipping, SBP load, and DBP load	SW was accompanied by early atherosclerotic changes in the vascular wall in children (I)
Amr et al (41)	Thickness increased vs controls; no difference between SW and SV; no correlation with mean daily hydrocortisone dose, treatment duration, and free testosterone concentrations	C-CAH was accompanied by early atherosclerotic changes in the vascular wall in children (I)
Borges et al (53)	No differences in thickness vs control men and women	C-CAH was not accompanied by early atherosclerotic changes in the vascular wall in young adults (N)
Delai et al (66)	No difference in thickness vs controls	It is difficult to draw conclusions because controls were older than patients by an average of 10 years (X)
Espinosa-Reyes et al (69)	Thickness and the percentage of patients with increased thickness insignificantly increased vs sex-, age-, and BMI-controls. More females than with increased thickness. Greater thickness in NC vs SW and SV	CAH may be accompanied by early atherosclerotic changes in the vascular wall in children and young adults (I)
Farghaly et al (76)	No difference in thickness vs controls; thickness increased in patients with poor control vs patients with good control; correlation with flow-mediated dilation	C-CAH was not accompanied by early atherosclerotic changes in the vascular wall in children (N)
Harrington et al (83)	No difference in thickness vs controls and obese subjects	C-CAH was not accompanied by early atherosclerotic changes in the vascular wall in young adults (N)
Kim et al (91)	No difference in thickness vs controls; increased in obese vs nonobese patients; thickness increased in male vs female patients; no differences between SW and SV; no difference between female patients with advanced bone age vs male patients without advanced bone age; positive correlation with androstenedione, 17OHP, and HDL cholesterol but not with DHEA-S, total and free testosterone, sex hormone–binding globulin, BMI, waist circumference, waist to hip ratio, leptin, insulin, HOMA-IR, the remaining lipid fractions, hsCRP, plasminogen activator-inhibitor-1, homocysteine, and family history of cardiovascular disease	C-CAH was not accompanied by early atherosclerotic changes in the vascular wall in children and young adults (N)
Metwalley et al (105)	Thickness increased vs controls (irrespectively of sex); correlations with age, duration of treatment, hsCRP, circulating endothelial cells, testosterone, and 17OHP but not with hydrocortisone dose equivalent	C-CAH was accompanied by early atherosclerotic changes in the vascular wall in children (I)
Metwalley et al (106)	Thickness increased vs controls; thickness higher in patients with poorly controlled than well-controlled CAH; correlation of intima–media thickness and homocysteine	C-CAH was accompanied by early atherosclerotic changes in the vascular wall in children (I)
Metwalley et al (107)	Thickness increased vs controls; correlation with epicardial fat thickness	C-CAH was accompanied by early atherosclerotic changes in the vascular wall in children (I)
Mnif et al (109, 110)	Thickness increased in 53.8% of patients vs general population; 11 noncompliant patients (42.3%) and 1 overtreated patient (3.8%) in the group of patients with increased intima–media thickness	CAH was accompanied by early atherosclerotic changes in the vascular wall (I)
Mooij et al (113)	Intima–media thickness within the reference range; thickness >75th percentile of reference values in only 1 out of 24 patients (4%)	CAH was probably not accompanied by early atherosclerotic changes in the vascular wall in children (N)
Özdemir et al (120)	Thickness increased vs controls; correlation of intima–media thickness with age, weight, height, BMI (independent), bone age, SBP, DBP, heart rate, aortic strain and distensibility, carotid elastic modulus, and treatment duration	CAH was accompanied by early atherosclerotic changes in the vascular wall (I)
Paizoni et al (121)	No difference in thickness vs controls; independent predictors: age, male sex, BMI, and waist to hip ratio	C-CAH was not accompanied by early atherosclerotic changes in the vascular wall in adults (N)
Rodrigues et al (128)	Thickness increased vs controls; no correlation with hydrocortisone dose, BMI, blood pressure, and metabolic parameters (androstenedione, lipids, and HOMA-IR)	C-CAH was accompanied by early atherosclerotic changes in the vascular wall of many arteries in children and young adults (I)
Rosenbaum et al (129)	No difference in thickness vs controls	CAH was not accompanied by early atherosclerotic changes in the vascular wall (N)
Sartorato et al (130)	Intima–media thickness of carotid arteries increased vs controls; increased also in carotid bulbs, abdominal aorta, and common femoral arteries; no differences related to gender, phenotype, and smoking habits; correlations of carotid artery, bulb, and femoral artery thickness with age and BMI; no correlation with fasting glucose, glucose, and insulin after glucose load, cumulative glucocorticoid dose, testosterone, androstenedione, 17OHP	C-CAH was accompanied by early atherosclerotic changes in the vascular wall of many arteries in young adults (I)
Tuhan et al (140)	No difference in thickness vs controls; no correlation with age, weight SDS, height SDS, BMI SDS, hydrocortisone or fludrocortisone doses, SBP SDS, DBP SDS, glucose, plasma lipids, 17OHP, and androstenedione	C-CAH was not accompanied by early atherosclerotic changes in the vascular wall in children (N)
Wasniewska et al (147)	Intima–media thickness of carotid arteries increased vs controls; increased also in carotid bulbs, abdominal aorta, and common femoral arteries; no differences between C-CAH and NC; positive correlations of carotid intima–media thickness and triglycerides and triglycerides/HDL cholesterol ratio; positive correlations of abdominal aorta thickness with cumulative glucocorticoid dose, triglycerides, and DBP SDS, and negative with androstenedione and ACTH	CAH was associated with early atherosclerotic changes in the vascular wall of many arteries in children and young adults (I)
Wierzbicka-Chmiel et al (150)	Intima–media thickness of carotid arteries increased vs controls; increased also in common femoral artery; insignificant after correction for: age, sex, body mass index, the dose of corticosteroid and fludrocortisone, total cholesterol, and smoking status; correlation with total testosterone but not with cumulative glucocorticoid dose, androstenedione, and 17OHP	SW was associated with early atherosclerotic changes in the vascular wall of many arteries in young adults (I)

Summary: Unfavorable impact of CAH on carotid intima–media thickness in 14 studies (60.9%), no impact in 8 studies (34.8%), favorable impact in 0 studies, while 1 study (4.3%) was inconclusive.

Abbreviations: 17OHP, 17-hydroxyprogesterone; BMI, body mass index; BP, blood pressure; CAH, congenital adrenal hyperplasia; C-CAH, classic congenital adrenal hyperplasia; DBP, diastolic blood pressure; DHEA-S, dehydroepiandrosterone-sulphate; HDL, high-density lipoprotein; HOMA-IR, homeostatic model assessment for insulin resistance index; hsCRP, high-sensitivity C-reactive protein; NC, nonclassic phenotype; SBP, systolic blood pressure; SDS, standard deviation score; SV, simple virilizing phenotype; SW, salt-wasting phenotype.

^*a*^Consequences for cardiometabolic health: I, increased cardiometabolic risk; N, no impact on cardiometabolic risk; D, decreased cardiometabolic risk; X, no conclusions concerning cardiometabolic risk can be drawn based on these finding.

The unfavorable impact of CAH on carotid intima–media thickness is also supported by the results of a meta-analysis carried out by Tamhane et al (179). The increase in carotid artery intima–media thickness was more pronounced in adults than in children and adolescents. Unfortunately, the studies included in this meta-analysis were characterized by heterogeneity and high risk of bias. The mean daily dose of glucocorticoids ranged from 9.0 to 26.5 mg/m^2^ of hydrocortisone equivalent, and, in some of them, the dose of fludrocortisone was not reported. Moreover, the age at which glucocorticoid therapy was initiated and the duration of follow-up were rarely specified. Hence, the authors of the meta-analysis were unable to draw unequivocal conclusions about the association between intima–media thickness and sex, glucocorticoid type, daily doses of glucocorticoids and fludrocortisone, duration of treatment and genotype (179).

Although most studies concentrated on the measurement in the carotid arteries, there are also reports on intima–media thickness in other locations (130, 147, 150). In all these studies, differences in the common carotid arteries between patients with CAH and controls were accompanied by similar differences in intimal–medial thickening in the carotid bulbs, abdominal aorta and common femoral arteries. However, no signs of progression to plaque formation were reported (130, 150), and despite differences compared with controls, intima–media thickness in the study by Wierzbicka-Chmiel et al was still within the reference range (150). Similarly, only 1 child of 24 with CAH (4%) had carotid intima–media thickness above the 75th percentile for age and sex (113). These findings indicate that although CAH may induce structural changes in both elastic and muscular arteries already in the early ages, these changes are not advanced. Another piece of evidence indicating that CAH exerts a detrimental effect on elastic properties of the ascending aorta and carotid arteries, as well as results in subclinical atherosclerosis was provided by Özdemir et al (120). In their study, the stiffness index and the elastic modulus of the ascending aorta and carotid arteries were higher in children with CAH than in controls, while the opposite relationship was observed for aortic and carotid distensibility. However, Rosenbaum et al did not observe any differences in pulse-wave velocity between adult patients with CAH and controls (129). The finding of no differences compared with controls may be related to the young mean age of their cohort (30.5 years) because in a recent study including patients with Addison disease, the median age of whom was 51 years, pulse-wave velocity was higher than in controls (226). Lastly, in children and adolescents with CAH, the ambulatory arterial stiffness index depended on the degree of skeletal maturity. This index was lower in individuals with normal bone age than in individuals with advanced bone age, suggesting an unfavorable effect of suboptimal glucocorticoid treatment. Interestingly, the ambulatory arterial stiffness index was higher in females with CAH than males with CAH and normal bone age, while no sex differences were noted for affected children with CAH and advanced bone age (86).

It is difficult to explain these differences in the results between various studies. Most of them included a small number of participants, and it appears that larger groups in some studies would ensure significant differences. The included populations differed in genetic background, which was found to be an independent predictor of arterial wall thickness (227). No increase in intima–media thickness was observed mainly in studies including only or predominantly children and adolescents. Endothelial dysfunction is the first step in the development of atherosclerosis (228), and therefore it is possible that, because of young age, the participants might not yet have developed structural changes in the arteries. This well explains why despite no increases in intima–media thickness, adolescents with CAH presented with impaired flow-mediated dilation (76, 83). Moreover, in the study by Delai et al no difference compared with controls may be a consequence of the 10 years older controls (66). Finally, the association between CAH and intima–media thickness probably in part depends on the level of metabolic control. Consequently, Farghaly et al and Metwalley et al observed that carotid artery intima–media thickness was greater in subjects with poorly controlled CAH than in well-controlled CAH (76, 105). Paizoni et al explained a neutral effect of CAH on intima–media thickness in many overtreated women with CAH and low or undetectable androgen concentrations by the greater role of androgens in thickening of the tunica intima and the tunica media than glucocorticoid exposure (121). Similarly, Borges et al did not report increased carotid intima–media thickness in subjects with C-CAH, in whom androgen concentrations were similar to those in healthy individuals (53).

There also exist other controversies concerning factors affecting the intima–media complex in individuals with CAH. In the study by Kim et al, including adolescents and young adults, carotid intima–media thickness was greater in obese than nonobese subjects, irrespective of whether they had CAH or not (91). Abdel Meguid et al reported that carotid intima–media thickness correlated with BMI and weight SDS (38). Wasniewska et al observed positive correlations between intima–media thickness and cumulative glucocorticoid dose, triglyceride levels and the triglyceride/HDL cholesterol ratio (147). Akyürek et al showed that carotid intima–media thickness was unrelated to plasma lipids, but negatively correlated with nocturnal DBP dipping (40). Finally, the predictors for intima–media thickness were age, male sex, BMI, and the waist to hip ratio (121). These correlations suggest that unfavorable changes in the intima–media thickness may be secondary to poor metabolic control of CAH and are in part a consequence of supraphysiological and/or nonphysiological glucocorticoid replacement. According to the alternative explanation, greater thickness may result from elevated androgen production. Intima–media thickness was found to correlate positively with circulating testosterone, androstenedione, and 17OHP concentrations (69, 91, 105). Moreover, despite higher mean intima–media thickness in men than women with CAH, there were no differences in intima–media thickness between females with CAH and advanced bone age and men with CAH who did not have advanced bone age (91). Finally, Özdemir et al found correlations between carotid intima–media thickness and bone age, SBP, DBP and treatment duration (120), suggesting that changes in the vascular wall may result from additive effects of several factors: increased androgen production, increased BP and replacement therapy. This question requires, however, better understanding because other authors reported that increased intima–media thickness in patients with CAH was independent of body mass, BP, cumulative doses of glucocorticoids, hormones, plasma lipids and glucose (110, 130).

In summary, CAH seems to predispose to increased intima–media thickness of various arteries, which may be at least partially the consequence of poor control and unphysiological glucocorticoid replacement. Despite early changes in the vascular wall, there are no data concerning the earlier and/or more pronounced development of atherosclerotic plaques, which suggests that cardiovascular complications of CAH cannot be explained exclusively by the progression of atherosclerosis.

## Low-Grade Inflammation

C-reactive protein (CRP) plays an important role in the development of atherosclerosis, being implicated in release of proinflammatory cytokines, promotion of endothelial dysfunction, induction of tissue factor expression, activation of complement pathway and inhibition of nitric oxide synthesis (229). Elevated levels are regarded as a sensitive marker of chronic low-grade inflammation (230). In numerous large-scale prospective trials, high-sensitivity CRP (hsCRP) concentrations strongly and independently predicted sudden cardiac death, myocardial infarction and stroke in individuals with and without coronary artery disease (229). The studies carried out so far have provided contradictory results on the association between CAH and low-grade systemic inflammation. No changes in hsCRP were observed in 30 Chinese female young adults with SV-CAH and without glucocorticoid therapy (153), as well as in 30 young patients with NC-CAH on low-dose glucocorticoid therapy (66). In the latter study, however, the participants with CAH were on average 10 years younger than the controls. There were no differences in hsCRP concentrations between adult patients with CAH receiving 24 ± 10 mg of hydrocortisone daily and controls (129). In the study by Ariyawatkul et al, hsCRP levels tended to be higher in patients with SW-CAH and SV-CAH than in controls, while no differences in interleukin-6 and leptin were observed (44). In the study by Metwalley et al, hsCRP concentrations were higher in adolescents with C-CAH than in controls (105). Moreover, Kurnaz et al observed higher concentrations of hsCRP in prepubertal children with C-CAH than in controls (99). Lastly, Farghaly et al reported elevated hsCRP concentrations, inversely correlating with brachial artery flow-mediated dilation, in young subjects (mean age 14.8 years) with C-CAH (76). Particularly high concentrations were observed if CAH was poorly controlled. However, Mooij et al observed lower concentrations of hsCRP in glucocorticoid-treated adult patients with C-CAH than in controls (112). It is difficult to explain these discrepancies. The most likely explanation is the association with anti-inflammatory and immune suppressive effects of chronic glucocorticoid treatment (112). No glucocorticoid use may explain the higher hsCRP concentrations observed in drug-naïve women with NC-CAH than in controls (98). Indirect evidence supporting the role of glucocorticoid treatment in determining hsCRP concentrations is that differences in daily hydrocortisone dose between 2 populations of individuals with Addison disease were associated with significant differences in hsCRP concentrations (lower levels in more intensively treated Swedes than in less intensively treated South Africans) (231). As mentioned above, elevated hsCRP may also be explained by androgen excess since androgen concentrations correlated with hsCRP concentrations in women with NC-CAH (98). Another explanation associated with differences in sex hormones was put forward by Kurnaz et al, who hypothesized that the degree of systemic inflammation depends on pubertal development (99). They observed that, unlike prepubertal children, at puberty hsCRP concentrations were only insignificantly higher in girls with C-CAH than in the controls and did not differ between boys with C-CAH and controls.

The remaining markers of inflammation were assessed only in 4 studies. Mooij et al did not observe differences in serum concentrations of interleukin-6 and interleukin-18 between adults with CAH and controls (112). Similar circulating levels of interleukin-6 in patients with CAH and controls were also reported by Ariyawatkul et al (44) and Rosenbaum et al (129). However, most participants of these studies were chronically treated with glucocorticoids, which might have masked between-group differences in cytokine production. Chronic glucocorticoid therapy may also explain no differences in interleukin-6 and tumor necrosis factor-α between individuals with NC-CAH and controls (66). Furthermore, we cannot exclude the impact of sex on the obtained results as women were overrepresented in these studies. Men and women differ in circulating levels of proinflammatory cytokines, and underrepresentation of men, in whom production of these cytokines in response to triggering stimuli is more pronounced than in women (232), may attenuate the impact of CAH itself. Enhanced production of proinflammatory cytokines may also be speculated on the basis of studies including other groups of patients with impaired functioning of the adrenal cortex. Increased levels of interleukin-6 were reported not only in patients with Addison disease (233), but also in patients with both primary and secondary AI (234), suggesting that increased concentration of interleukin-6 are a consequence of not only autoimmune destruction of the adrenal cortex. In the latter study, interleukin-6 levels exceeded those found in controls, and, unlike controls, they were characterized by an increase toward the evening and night. This finding suggests an abnormal diurnal pattern of interleukin-6 secretion (and maybe also of other cytokines), which may be difficult to reveal if blood samples are taken once daily in the morning.

## Adipose Tissue Hormones

Adipose tissue is a highly active metabolic and endocrine organ secreting a range of bioactive peptides with both local and distant actions known under the name of “adipokines” (235). At least some of them are involved in regulating lipid metabolism, insulin sensitivity, and vascular homeostasis, and their abnormal production may contribute to the development of atherosclerosis and its complications (236).

The relationship between CAH and adipokine concentrations was limited to the assessment of only leptin and adiponectin. For this reason and because of inconsistent results of studies conducted so far (44, 52, 53, 58, 66, 112, 124, 129, 131, 145, 146, 153), the association between CAH and hormonal function of adipose tissue is far from fully understood. Compared with controls, patients with C-CAH in the study by Charmandari et al had higher serum leptin concentrations, and this difference persisted after correction for BMI, despite the fact that BMI was the only independent predictor of leptin concentration (58). Moreover, unlike controls, the authors did not observe differences in leptin between males and females. They speculated that increased leptin concentrations together with other hormonal changes (increased insulin concentration as well as reduced epinephrine/metanephrine concentrations) contribute to a further increase in androgen production. Low epinephrine/metanephrine concentrations probably reflected adrenomedullary failure, leading by itself to an increase in leptin production (237). Higher leptin concentrations in individuals with CAH than in controls, but only in men, were reported also by others (52, 53). Völkl et al observed that, despite unaltered serum leptin concentrations, serum concentration of the soluble leptin receptor (the main binding protein of leptin) and the soluble leptin receptor to leptin ratio was lower in children and adolescents with C-CAH than in controls (145). Similar leptin concentrations were found in both boys and girls (144). Interestingly, there were no differences in leptin concentrations, the soluble leptin receptor and the soluble leptin receptor to leptin ratio between individuals with SW-CAH and with SV-CAH. Unaltered leptin concentration was reported also in adults with CAH (112), children and young adults with C-CAH (44), glucocorticoid-treated patients with NC-CAH (66), and in glucocorticoid-naïve women with NC-CAH (131). The role of glucocorticoid treatment and androgen status in regulation of leptin production in CAH is supported by the results of an interventional study by Poyrazoglu et al (124). Untreated children with CAH had serum leptin concentrations lower than controls (124). However, hydrocortisone, administered in 45% together with fludrocortisone, led to an increase in leptin concentrations, which was paralleled by an increase in serum cortisol and a reduction in serum testosterone concentrations (124).

In the study by Völkl et al, children and adolescents with C-CAH demonstrated increased adiponectin concentrations compared with controls, with no difference between patients with SW-CAH and SV-CAH (146). However, the adiponectin to leptin ratio did not differ from controls (146). A tendency toward an increase in adiponectin concentration was observed by Mooij et al in adults with CAH on stable glucocorticoid and mineralocorticoid therapy for 3 months (112). Furthermore, in the study by Zhang et al adiponectin concentrations were lower in untreated young female adults with SV-CAH than in the controls and negatively correlated with testosterone concentrations (153). The latter observation may be explained by the impact of androgen excess because the glucocorticoid-naïve women with CAH had markedly elevated androgen concentrations (153), which were found to downregulate adiponectin expression (238). Interestingly, lower adiponectin concentrations were observed in patients with NC-CAH treated with low doses of various glucocorticoids (66). However, others did not observe any differences in adiponectin levels between adults with CAH and controls (52, 53, 129).

It is possible that the inconsistent results of the above-mentioned studies arise from different baseline characteristics of the studied populations, various androgen status in the investigated groups, differences in glucocorticoid treatment, regulation of leptin and adiponectin production by a complex matrix of factors, and assessment in only a single time point.

## Hemostasis

Disturbances of coagulation and fibrinolysis play an important role at different stages of atherogenesis, contributing to the development and progression of atherosclerosis, and to the incidence of atherosclerosis-related clinical events (239, 240). High circulating concentrations or activities of hemostatic factors, particularly fibrinogen, plasminogen activator inhibitor-1, and tissue plasminogen activator, are associated with increased cardiovascular morbidity and mortality (239).

Very little is known about the association between CAH and hemostasis. Plasminogen activator inhibitor-1, tissue-type plasminogen activator, urokinase-type plasminogen activator, and plasminogen activator inhibitor-1–tissue-type plasminogen activator complexes were not different in 27 adults with CAH and 27 BMI-matched controls (112). In contrast, untreated women with NC-CAH displayed increased fibrinogen levels compared with controls (98). Finally, in adolescents and young adults with CAH, plasminogen activator inhibitor-1 levels correlated with amounts of visceral and subcutaneous fat (90).

Only 2 studies investigated platelet count and function in individuals with CAH. Untreated infants aged 6-60 days with C-CAH were found to have increased platelet count, which correlated with 17OHP concentrations but was not associated with hemoconcentration (78). Although this observation suggests that the platelet count may be a marker of severity of C-CAH in early infancy, the results were not supported by the second study. Platelet count and platelet aggregations in response to adenosine diphosphate, collagen, and epinephrine did not differ between young women with NC-CAH and controls (67). The authors did not mention, however, if the participants were treated with glucocorticoids or antiandrogens, despite the fact that these treatments may theoretically affect production, degradation, and function of platelets.

Individuals with CAH had increased prevalence of venous thromboembolism compared with controls (74), or at least women with CAH aged 40 years or older (161). Thromboembolic events were also reported more frequently in patients with Cushing syndrome and patients on long-term glucocorticoid therapy, resulting from a state of hypercoagulability, obesity, and hypertension (241, 242). Theoretically, the same predisposing factors may make patients with CAH more prone to thromboembolic complications and some individuals with CAH may benefit from thrombosis prophylaxis. Because of a limited number of studies, differences in the obtained results, and methodological limitations, no firm conclusions can be drawn regarding the impact of CAH on hemostasis. Considering that the unfavorable effect of CAH on hemostatic parameters (90, 98) is predictive of future cardiovascular morbidity and mortality, this question needs to be better addressed.

## Remaining Cardiometabolic Risk Markers

Elevated homocysteine concentrations are regarded as a risk factor for atherosclerosis, cardiovascular disease, and stroke (243). Only 4 studies compared homocysteine concentrations between individuals with CAH and controls, providing inconsistent results. Unlike elevated levels in women with PCOS, females with NC-CAH had similar homocysteine concentrations to those observed in BMI-matched controls (49). In turn, untreated women with NC-CAH were characterized by higher concentration of homocysteine than weight-matched apparently healthy peers (98). In children with C-CAH, homocysteine concentrations were higher than in controls (106). These concentrations were also higher in individuals with poorly than well-controlled CAH, and correlated with SBP, DBP, the atherogenic index, HOMA-IR, the left ventricular mass index, the mitral deceleration time, and intima–media thickness (106). Interestingly, adult men with CAH younger than 30 years old had lower homocysteine concentrations than controls (72).

Prospective observational studies indicate that in the general population low vitamin D status predicts a greater risk of cardiovascular morbidity and mortality, diabetes, metabolic syndrome, and all-cause mortality (244). In a study by Falhammar et al, there were no differences in 25-hydroxyvitamin D concentrations between adult Swedish males with CAH and controls (73). It should be underlined, however, that subjects with CAH much more frequently received exogenous vitamin D preparations than their controls (73). In a large CAH study, the percentage of individuals with vitamin D deficiency and insufficiency was similar to that described in the overall US population (77). However, Demirel et al reported higher vitamin D deficiency and insufficiency rates in Turkish children and adolescents with CAH when compared with normal healthy children, and the degree of deficiency increased with age (245). In other studies, 25-hydroxyvitamin D concentrations in individuals with CAH were lower than in the general population (119), in the lower normal range (92), or below the lower limit of normal (125). Although unexplained, these incongruent results may be associated with various glucocorticoid regimens, between-group differences in supplementation of vitamin D, using other agents that may influence vitamin D pharmacokinetics, skin color, and sun exposure. Glucocorticoids affect vitamin D homeostasis at different levels, inhibiting its absorption, decreasing synthesis of active vitamin D and impairing its biological action at the tissue level (246). However, lower 25-hydroxyvitamin D levels in glucocorticoid-naïve women with NC-CAH than in controls (98) suggest the involvement of other mechanisms underlying low vitamin D status in CAH.

Another predictor of cardiovascular morbidity and mortality independent of other cardiovascular risk factors is albuminuria (247). Urinary albumin concentrations tended to be higher in adult men with CAH aged 30 years or older and in men treated with hydrocortisone or cortisone acetate than in controls (72). Elevated values of the urinary albumin to creatinine ratio were also found in women with NC-CAH (98). However, no difference in albumin excretion compared with controls was observed in 27 adults with CAH (112).

## Rhythm and Conduction Disturbances

Increased heart rate is a risk factor for cardiovascular morbidity and mortality, independent of other risk factors (248, 249). Even small increases, not exceeding the upper limit of normal, are associated with higher prevalence of cardiovascular events (249). Unfortunately, data concerning heart rate in individuals with CAH are inconsistent. In 2 studies, there were no differences in heart rate compared with controls during exercise and recovery (80, 126). However, Tuhan et al observed insignificantly higher resting heart rate in children and adolescents with CAH (139). In the study by Mooij et al, mean 24-hour heart rate was higher in adults with CAH than in BMI-matched controls (112). Weise et al reported a 5% lower peak heart rate in adolescents with CAH (148), which might have been a consequence of adrenomedullary failure. Falhammar et al reported increased 24-hour heart rate in men with CAH aged 30 years or older (by 27 and 13 beats per minute during night and day, respectively), but not in younger ones (72). The increase was observed in patients with I2G and I172N genotypes but not with null genotype, and heart rate did not depend on the type of glucocorticoid. The authors calculated that elevated heart rate in about 50% was determined by low testosterone and high glycated hemoglobin (HbA_1c_) levels, possibly due to using too high doses of glucocorticoids in patients with milder disease (72). Finally, Kroese et al observed lower 24-hour and nighttime, but not daytime, heart rate during treatment with pioglitazone (94). The small number of studies and their inconsistency make it difficult to draw strong conclusions about heart rate in CAH.

To the best of our knowledge, only 2 studies have investigated other rhythm and conduction disturbances in subjects with CAH. Atrial fibrillation and flutter were recorded more frequently in individuals with CAH than in controls, particularly in males aged at least 40 years and subjects with the I172N genotype (74). This increase may be partially explained by alcohol misuse, precipitating atrial fibrillation (250, 251). Moreover, Mooij et al, assessing 27 children with CAH, observed incomplete right bundle branch block in 26%, as well as the lack of a sinus rhythm in 2 patients (having either atrial or sinoatrial rhythm) (114). The clinical relevance of these findings is difficult to establish. Incomplete right bundle branch block is a frequent finding in the general population, may be transient and is not related to increased cardiovascular mortality (252, 253), while atrial and sinoatrial rhythms were reported so rarely that their association with CAH might have been accidental.

## Cardiac Structure and Function

The evidence is convincing that imbalances in glucocorticoid receptor and mineralocorticoid receptor signaling in the heart cause heart disease (254). Glucocorticoids influence the blood vessels by glucocorticoid receptor regulating a myriad of signaling pathways. These pathways play roles in development, angiogenesis, oxidative stress, and inflammation within vascular smooth muscle cells and endothelial cells (255, 256). Clinical studies indicate that reduced systemic glucocorticoid receptor signaling is linked to diminished cardiac contractile force, systolic dysfunction, coronary artery disease, dilated cardiomyopathy, and the advancement toward heart failure (254, 257-259). Many of these studies have a limitation in that they fail to differentiate between the systemic effects of glucocorticoids and their direct local impact on the heart and blood vessels (254).

A pilot study including 9 newborns with C-CAH revealed that heart failure was already observed in first days of life, however, early introduction of glucocorticoid replacement completely reversed it (108). The mechanism underlying heart failure in untreated patients has not been fully explained, but it may be associated with hemoconcentration (decreasing coronary blood flow), dyselectrolytemia (hyperkalemia or hyponatremia), and impaired glucocorticoid and/or mineralocorticoid action at the level of cardiomyocytes (260). Moreover, glucocorticoid receptor–deleted mice had impaired cardiac contractibility and cardiac hypertrophy (261). There is no evidence that transient disturbances in cardiac contractility in the early childhood are associated with higher rates of future cardiovascular events.

There are no convincing data that systolic dysfunction develops in the patients with CAH more frequently than in the general population. The left ventricular ejection fraction, stroke volume, cardiac output, cardiac index, and fractional shortening did not differ between individuals with CAH and controls (53, 105, 137). However, Mooij et al reported an insignificant increase in the global longitudinal strain, inversely correlating with 24-hour BP (114). This observation justifies further investigation because global longitudinal strain is an earlier and a more sensitive marker of left ventricular systolic dysfunction than left ventricular ejection fraction and is useful in risk stratification of various cardiac diseases affecting the left ventricle (262). In line with this finding, Tuhan et al reported the prolonged isovolumic contraction time and the increased myocardial performance index (139), suggesting the presence of subclinical left ventricular systolic dysfunction. Recently, Amr et al found a lower fractional shortening percentage in patients with C-CAH than in controls (42). Since the 3 last studies only included children and adolescents with CAH, it is prudent to assess parameters of systolic function also in adults with CAH.

A more consistent finding associated with heart function is left ventricular diastolic dysfunction. Left ventricular diastolic dysfunction has been observed already in children and adolescents with CAH (42, 104, 105, 120, 137, 139), although Marra et al reported its development exclusively in males (104). Left ventricular dysfunction was found to coexist with impaired exercise performance, as well as with enhanced SBP response during exercise (104). It should be underlined that left ventricular diastolic dysfunction is 1 of the first echocardiographic abnormalities appearing in subjects with atherosclerotic cardiovascular disease (263) and therefore its presence in individuals with CAH may have clinical significance. In line with this, a recent large-scale study showed that diastolic dysfunction was associated with increased risk of all-cause mortality and cardiovascular mortality (even in patients with preserved left ventricle ejection fraction) (264). Interestingly, mitral and tricuspid velocities were worse in patients with CAH receiving glucocorticoid supplementation for more than 6 years, correlated with the duration of treatment, cumulative glucocorticoid dose, and mean daily glucocorticoid dose in mg/m^2^ per day (42), suggesting a possible causative role of supraphysiological glucocorticoid replacement.

Mild diastolic dysfunction was present in children with CAH even if insulin concentrations and HOMA-IR were within the reference range (104), and therefore its association with impaired insulin sensitivity does not seem convincing. The risk of diastolic dysfunction in children with CAH was reported to be inversely correlated with testosterone concentrations (104, 105), which suggests that unfavorable changes in the heart may be a consequence of decreased androgen production most likely resulting from glucocorticoid overtreatment (34). Alternatively, high doses of glucocorticoids directly destroy cardiomyocytes, while low testosterone concentrations are only an epiphenomenon of supraphysiological glucocorticoid replacement. In line with this explanation, the isovolumetric relaxation time correlated with the hydrocortisone dose even after adjustment for androgen concentrations (139), while testosterone concentrations inversely correlated with the average hydrocortisone dose within 3 years before the study (104). Moreover, the causative role of elevated BP, observed in many patients with CAH, in inducing diastolic dysfunction is supported by the finding of left ventricular failure in a 6-year-old boy with hypertension, which was secondary to 11β-hydroxylase deficiency (265).

Data concerning ventricular mass in individuals with CAH are inconclusive. Some studies observed a higher thickness of the left ventricular posterior wall and the interventricular septum, as well as increased left ventricular mass and left ventricular mass index (42, 105, 139), which suggest the presence of hypertrophy of this heart chamber. Interestingly, CAH was poorly controlled in most participants of these studies. The elevated left ventricular mass index was observed also in children with CAH in Cameroon, the majority with delayed CAH diagnosis (137). However, others did not report ultrasound changes suggestive of left ventricular hypertrophy (53, 104, 141, 150) nor showed a reduced thickness of the left ventricular posterior wall (114). Intriguingly, myocardial hypertrophy parameters positively correlated with testosterone concentrations in children with CAH (104, 105). Therefore, the most probable explanation for these contradictory results is that the risk of hypertrophy may depend on the length of exposure androgen excess. If individuals are correctly diagnosed neonatally and effectively treated, they do not develop left ventricular hypertrophy (53, 114). However, if diagnosis and treatment are delayed, chronic androgen excess may have a detrimental effect on the myocardium, leading to hypertrophy of the left ventricle. Later glucocorticoid replacement may cause that at the time of assessment correlations between androgens and echocardiographic markers of cardiac hypertrophy may be absent (42). Consequently, early detection of CAH and implementation of glucocorticoid replacement may be beneficial also from a cardiological point of view. Lastly, glucocorticoid excess may by itself induce myocardial remodeling and cause left ventricular hypertrophy (261). Thus, the doses of glucocorticoids should be carefully chosen in order not to exceed the therapeutic window.

Contrary to the left ventricle, pilot observations suggest that CAH does not seem to impair the function of the right ventricle (120, 137). The lack of right ventricular dysfunction seems to be an argument against the role of androgen excess as a sole factor determining diastolic left ventricular dysfunction in subjects with CAH, suggesting that elevated BP may also contribute to left ventricular dysfunction (93).

Interestingly, until now, no patient with CAH had been found to develop Takotsubo cardiomyopathy, in contrast to other patients with AI (266, 267). The reason why no case of Takotsubo cardiomyopathy has been reported in CAH may be attributed to higher doses of glucocorticoids in the treatment of CAH than of other forms of AI, or to differences in sympathetic tone and/or function of the adrenal medulla.

In summary, CAH is likely to lead to diastolic dysfunction of the left ventricle, which may be partially explained by thickening of its walls and is associated with adverse treatment effects and its later implementation. This dysfunction may be potentiated by other cardiometabolic effects of CAH, particularly elevated BP, increased adipose tissue content, and impaired insulin sensitivity. The unfavorable impact on systolic function of this chamber and on right ventricular function, as well as on exercise capacity is less convincing and requires further research.

## Exercise Capacity

Three pilot studies reported that patients with C-CAH did not differ from controls in exercise capacity, irrespective of whether it was assessed during short-term high-intensity exercise, measuring maximal aerobic capacity (126, 148), or during long-term moderate-intensity exercise that is equivalent to brisk walking (80). Only Marra et al observed that adolescents with C-CAH exhibited an impaired exercise capacity as shown by reduced peak workload and higher SBP response at peak during an exercise test (104). Interestingly, the authors observed similar exercise capacity (and diastolic function) in overtreated and in normotreated or undertreated patients (104). Study differences in exercise performance of patients with CAH may be attributed to more patients with CAH in the study by Marra et al (20 persons) than in the remaining studies (from 6 to 9 persons) and maybe also to differences in insulin sensitivity between the study populations. Despite similar exercise capacity, individuals with CAH had a blunted increase in plasma glucose concentrations (126, 148), a lower peak heart rate (148), and the lack of leptin suppression (126) during high-intensity short-term exercise and by a steady decline in glucose concentrations during a standardized moderate-intensity exercise test (80). However, neither high-intensity nor moderate-intensity exercise caused overt hypoglycemia (80, 126, 148). All these disturbances seem to be the result of abnormally low epinephrine secretion, resulting from decreased adrenomedullary reserve and impaired conversion of epinephrine to norepinephrine, which, as has been mentioned earlier, are secondary to low cortisol production (72, 193, 194).

## Special Considerations Concerning NC-CAH

There are substantial differences between NC-CAH and C-CAH that may be important from the cardiometabolic point of view. Firstly, most individuals with NC-CAH (including almost all men) remain undiagnosed (13). This means that they are exposed life-long to supraphysiological adrenal androgen production. Secondly, in untreated NC-CAH, cortisol production is often normal or near-normal, and aldosterone production is unaltered. Thirdly, in contrast to C-CAH, only some individuals with NC-CAH (children with accelerated bone maturation, patients presenting symptoms of hyperandrogenism, and women planning pregnancy) are treated with glucocorticoids (17). However, treatment period is usually shorter (from months to several years), and individuals with NC-CAH often receive long-acting synthetic glucocorticoids (268). Because the diagnosed cases (mainly symptomatic women) represent only a tip of the iceberg, the data concerning prevalence or incidence of its complications are affected by ascertainment bias. Unfortunately, no large-scale longitudinal study has investigated cardiometabolic outcomes in NC-CAH. Falhammar et al reported increased risk of any cardiovascular disease, obesity, diabetes, and stroke in individuals (mainly women) with NC-CAH than in controls (74). In turn, in a study by Liu et al, obesity was reported in 10.3%, overweight in 23.1%, insulin resistance in 41%, type 2 diabetes in 9%, impaired glucose tolerance in 29.5%, metabolic syndrome in 1.3%, and dyslipidemia in 32.1% of women with NC-CAH (102). Unfortunately, the presented data provide only general information concerning NC-CAH women probably with more pronounced 21OHD receiving glucocorticoids. These data may, however, be poorly representative because of the heavy underrepresentation of men, underrepresentation of asymptomatic/oligosymptomatic women, and a possible association with glucocorticoid overtreatment.

Most studies conducted so far included only or mainly individuals with C-CAH, while patients with NC-CAH were either excluded or constituted a minority, and often received glucocorticoids. Only a few studies have assessed cardiometabolic risk factors in glucocorticoid-naïve patients (unfortunately only women) with NC-CAH. Circulating levels of hsCRP, fibrinogen, and homocysteine were higher in young women with untreated NC-CAH than in controls, while the opposite relationship was observed for 25-hydroxyvitamin D (98). This finding suggests increased cardiometabolic risk in untreated women with NC-CAH. Abnormal levels of all these factors correlated with circulating concentrations of androgens (dehydroepiandrosterone-sulphate [DHEA-S], total testosterone, and androstenedione), steroid precursors (17OHP), and with HOMA-IR (98), indicating that cardiometabolic risk may be proportional to the degree of 21OHD. In 2 other studies, glucocorticoid-naïve women with NC-CAH were more insulin-resistant than female controls of similar age and weight (134). In the study by Saygili et al, insulin positively correlated with free testosterone and 17OHP concentrations (131). Thus, it cannot be excluded that hyperandrogenemia and impaired insulin sensitivity in CAH reciprocally potentiate their cardiometabolic effects, forming a mechanism of a vicious circle. However, in another Turkish study, untreated females with NC-CAH did not differ in glucose homeostasis markers and plasma lipids from BMI-matched healthy controls (49). In the Turkish population, women with NC-CAH were found to have unaltered leptin (131) and homocysteine (49) concentrations. Between-study differences in the obtained results may be a consequences of population differences. Women participating in both Turkish studies were younger, had lower BMI and the exclusion criteria were less restrictive than in the study by Krysiak et al (98).

Changes in body mass and composition in patients with NC-CAH may appear already in childhood. In a recent study, Ben Simon at al reported higher BMI *z*-score and increased content of fat and truncal fat in a population of 75 patients with NC-CAH, 81% of which received relatively small doses of glucocorticoids (hydrocortisone median dose: 6.59 mg/m^2^), compared with matched controls (50). The median age was only 11.2 years, and this may explain why they did not find differences in fasting glucose, lipids, and BP. However, despite young age, the study groups differed in the use of oral contraceptives, which was higher in the patients with NC-CAH (18.2%) than in controls (3.7%). In another recent study, Delai et al observed an increased value of the waist to hip ratio, slightly decreased concentrations of fasting glucose, HbA_1c_, HDL cholesterol, and adiponectin, and a reduced amount of exogenous glucose necessary to fully compensate for exogenous hyperinsulinemia (66). However, all subjects with NC-CAH either used glucocorticoids (59%) or reported prolonged use of these agents in the past. Moreover, women with NC-CAH used combined oral contraceptives more frequently (36%) than controls (18%) (66). Interestingly, both studies included not only females but also males (35% and 17%, respectively), yet the authors did not perform separate analyses for each sex (50, 66). Such analysis, as well as assessment of cardiometabolic risk factors in men with NC-CAH would be interesting. The vast majority of men with NC-CAH are undiagnosed and, consequently, do not receive any pharmacotherapy (13, 17), but it is not certain whether, from a cardiometabolic point of view, this is the best approach.

Very preliminary findings suggest that statin and/or metformin therapy is safe and may bring some cardiometabolic benefits to young glucocorticoid-naïve women with NC-CAH in case of cardiometabolic comorbidities (Table 7). Metformin decreased fasting glucose, HOMA-IR, HbA_1c_, and triglycerides in females with coexistent type 2 diabetes (95). The effect of metformin on plasma lipids (total and LDL cholesterol) was found to be stronger after simvastatin add-on therapy (96). In turn, atorvastatin, beyond decreasing elevated concentrations of total and LDL cholesterol, reduced in a lipid-independent manner plasma concentrations of hsCRP, homocysteine, and uric acid (97). These studies do not allow us, however, to formulate an analogous statement for glucocorticoid-naïve men and postmenopausal women with NC-CAH, and for subjects receiving glucocorticoids, which at supraphysiological doses increase risk of future cardiovascular events (273).

**Table 7.** The impact of nonspecific interventions on markers of cardiometabolic risk in patients with congenital adrenal hyperplasia

Intervention	Population	Body mass/composition	Glucose homeostasis	Plasma lipids	Blood pressure	Other effects	Reference
Pioglitazone (45 mg daily for 16 weeks)	CAH (C-CAH in all but 1 case) + glucocorticoid-induced insulin resistance (n = 12)	↔ subcutaneous fat ↔ visceral fat ↔ visceral to subcutaneous fat ratio ↓ % liver fat only in subjects with high baseline % liver fat	↑ glucose infusion rate ↑ insulin sensitivity index ↓ HOMA-IR (insignificant) ↓ area under the curve for insulin in oral glucose tolerance test ↔ area under the curve for glucose in oral glucose tolerance test	not assessed	↓ 24-hour, daytime and nighttime SBP ↓ 24-hour, daytime and nighttime DBP ↓ 24-hour, daytime and nighttime mean blood pressure	↓ 24-hour heart rate ↔ daytime heart rate ↓ nighttime heart rate	(94)
Metformin (2.55-3 g daily for 6 months)	NC-CAH + type 2 diabetes (n = 8)	not assessed	↓ fasting glucose ↓ HOMA-IR ↓ HbA_1c,_ No difference in percent changes in fasting glucose, HOMA-IR and HbA_1c_ vs patients with normal adrenal function	↓ triglycerides ↔ total cholesterol ↔ HDL cholesterol ↔ LDL cholesterol No difference in percent changes in triglycerides vs patients with normal adrenal function	not assessed	not assessed	(95)
Metformin (1 g daily for 3 months)	SW-CAH + overweight (n = 1)	↓ body weight	not assessed	not assessed		not assessed	(269)
Simvastatin (20 mg daily for 12 weeks)	NC-CAH + hypercholesterolemia + diabetes or impaired glucose tolerance + chronic metformin treatment (1.7-2.55 mg daily) (n = 8)	not assessed	↔ fasting glucose ↔ HOMA-IR ↔ HbA_1c_	↓ total cholesterol ↓ LDL cholesterol ↔ HDL cholesterol ↔ triglycerides no difference in percent changes in total and LDL vs patients with normal adrenal function	not assessed	not assessed	(96)
Atorvastatin (20-40 mg daily for 12 weeks)	NC-CAH + hypercholesterolemia (n = 12)	not assessed	↔ fasting glucose ↔ 2-hour postchallenge glucose ↔ HOMA-IR	↓ total cholesterol ↓ LDL cholesterol	not assessed	↓ hsCRP ↓ uric acid ↓ homocysteine ↑ 25OHD (insignificant) ↓ urinary albumin to creatinine ratio ↔ fibrinogen	(97)
Topiramate (50-100 mg for 4 years)	SW-CAH + obesity (n = 1)	↓ BMI *z*-score ↓ total body fat ↓ visceral adipose tissue	not assessed	not assessed	not assessed	not assessed	(270)
Bariatric surgery (sleeve gastrectomy)	SW-CAH + obesity (n = 1)	↓ weight ↓ BMI ↓ waist circumference ↓ waist to hip ratio ↓ decreased liver fat content	↓HOMA-IR	not assessed	not assessed	↓ daily hydrocortisone dose requirement	(271)
Bariatric surgery (Roux-en-Y gastric bypass)	NC-CAH (11OHD) + obesity + type 2 diabetes (n = 1)	↓ BMI	normoglycemia (without treatment with previously used hypoglycemic drugs)	not assessed	not assessed	not assessed	(272)

Abbreviations: 11OHD, 11β-hydroxylase deficiency; 25OHD, 25-hydroxyvitamin D; BMI, body mass index; CAH, congenital adrenal hyperplasia; C-CAH, classic congenital adrenal hyperplasia; HbA_1c_, glycated hemoglobin; HDL, high-density lipoprotein; HOMA-IR, homeostatic model assessment for insulin resistance index; hsCRP, high-sensitivity C-reactive protein; LDL, low-density lipoprotein; NC-CAH, nonclassic congenital adrenal hyperplasia; SW-CAH, salt-wasting congenital adrenal hyperplasia.

In summary, cardiometabolic risk in men with NC-CAH remains unknown. This risk may be increased in glucocorticoid-naïve women with more severe forms of NC-CAH or in case of oligosymptomatic women receiving glucocorticoids. Unfortunately, our conclusions are based on data that may be biased by underreporting or misdiagnosis of NC-CAH.

## Advances in Treatment and Cardiometabolic Risk

Existing glucocorticoid treatment for CAH is suboptimal and nonphysiological, often resulting in inadequate suppression of androgens and glucocorticoid excess (1, 2, 21, 22). In order to minimize unfavorable effects caused by conventional glucocorticoid supplementation in various forms of AI, 3 treatment options aimed at mimicking normal circadian cortisol rhythm have been developed: an immediate-release tablet with sustained-release hydrocortisone core formulation (SR-HC, Plenadren), a modified-release formulation of hydrocortisone (MR-HC, Chronocort/Efmody), and 24-hour circadian continuous subcutaneous infusion of hydrocortisone (Table 8). Unlike patients with primary and secondary AI, a dual-release preparation of hydrocortisone (ie, SR-HC, taken once daily in the morning) is unlikely to be suitable for patients with CAH because it does not replicate the preawakening rise in cortisol, and its impact on androgen production is relatively weak (274, 275). Thus, cardiometabolic effects of SR-HC have not been investigated in subjects with CAH.

**Table 8.** Arguments for and against cardiometabolic benefits of emerging treatment options for congenital adrenal hyperplasia

Treatment option	Arguments for	Arguments against
MR-HC	Preawakening rise in cortisol Better biochemical control of CAH Prevention of ACTH-driven excess production of adrenal androgens Possible lower daily hydrocortisone equivalent dose	Cardiometabolic benefits have not been proved yet Theoretically possible worsening of morning glucose homeostasis (higher early-morning glucose levels)
24-hour circadian subcutaneous infusion of hydrocortisone	Moderate morning ACTH peak Near physiological patterns of circadian and ultradian rhythmicity of cortisol Decrease in daily hydrocortisone equivalent dose Better androgen control	Cardiometabolic benefits have not been proved yet
Corticotropin-releasing factor type 1 receptor antagonists	Reduction of morning ACTH rise Better control of androgen production Possible reduction in glucocorticoid dose	Cardiometabolic benefits have not been proved yet
Abiraterone acetate	Control of androgen levels Theoretically possible reduction in glucocorticoid dose	Hypertension (in 21OHD probably absent)
Oral combined estrogen–progestin contraceptives	Antiandrogenic action	Concerns about thromboembolic complications Increase in triglycerides Higher fasting glucose in patients receiving pills containing desogestrel
Spironolactone	Antiandrogenic action Cardiovascular benefits in patients with hypertension and preexisting heart diseases	Antimineralocorticoid action

Cardiometabolic effects of MR-HC have been assessed only in a few studies including patients with CAH, and they were not determined in other forms of AI. In accordance with current recommendations (276), in all conducted studies, this formulation was given in 2 daily doses (one-third taken at 7 Am and two-thirds taken at 11 Pm). Replacement of conventional glucocorticoid treatment with MR-HC in patients with SW-CAH was not associated with any changes in SBP and DBP, though the replacement reduced plasma renin activity (277). This discrepancy cannot be explained by differences in daily glucocorticoid dose, but it might have been associated with a reduction in 17OHP concentrations. In another study including patients with C-CAH, MR-HC increased HOMA-IR, but this effect, observed already after the first dose, did not reflect changes in body mass and composition, and might have resulted from an early-morning increase in cortisol concentrations, which is absent in patients receiving conventional glucocorticoid therapy (278). Lastly, a recent 6-month, randomized, phase 3 study, followed by a single-arm extension study showed that subjects receiving MR-HC did not differ from patients continuing conventional glucocorticoid replacement therapy in fat mass, fasting glucose, fasting insulin, HOMA-IR, HbA_1c_, and hsCRP (279). In the same study, however, MR-HC improved morning and early afternoon biochemical control of 17OHP over standard glucocorticoid therapy, and this control was sustained for 18 months on hydrocortisone doses recommended for adrenal replacement therapy and lower than doses normally used in CAH (279). There are some possible explanations for these discrepancies: metabolic effects of MR-HC were not the primary endpoint, patients were allowed to take other drugs affecting androgen concentrations (except for spironolactone), most comorbidities were not considered an exclusion criterion, while, except for the extension study, the average hydrocortisone equivalent dose was relatively high (25.0-25.9 mg/day) and similar to that used by patients continuing conventional glucocorticoid therapy.

Because pulsatile cortisol secretion plays a role in glucocorticoid gene signaling (280), another emerging treatment approach is subcutaneous infusion of hydrocortisone. In patients with CAH, continuous subcutaneous hydrocortisone infusion resulted in a moderate morning ACTH peak (less pronounced than in patients on conventional oral hydrocortisone), while levels of steroids with diurnal fluctuations returned to baseline levels around 11 Am and remained stable throughout the rest of the day (281). Moreover, in 9 patients with difficult to treat AI (including 1 with CAH), replacement of oral hydrocortisone with continuous subcutaneous hydrocortisone infusion, beyond better disease control and the improved quality of life, led to a decrease in daily hydrocortisone equivalent dose by 34% (282). Unfortunately, metabolic effects of hydrocortisone infusion were assessed only in 2 studies. Mallappa et al reported that replacement of standard glucocorticoid therapy with continuous subcutaneous hydrocortisone infusion did not induce any changes in weight, BMI, waist to hip ratio, HOMA-IR as well as visceral and subcutaneous fat (283). However, only 5 individuals participated in this study, the daily hydrocortisone dose was supraphysiological (38.3 ± 8.8 and 33.6 ± 12.2 mg/day after 6 and 18 months of therapy, respectively), and all included females were initiated during the study on combined oral contraceptives, the use of which may have affected metabolic effects of hydrocortisone infusion. In a study by Mortensen et al switching from conventional hydrocortisone to continuous subcutaneous hydrocortisone infusion resulted in a reduction in body weight in 5 out of 8 patients with AI (63%), though average BMI did not change (282). Interestingly, a recent study by Simunkova et al has shown that mimicking endogenous cortisol rhythmicity by ultradian subcutaneous infusion of hydrocortisone is superior not only to oral glucocorticoid therapy but also to continuous pump therapy in maintaining normal ACTH concentrations and in restoring circulating and subcutaneous tissue cortisol throughout the 24-hour cycle (284). However, the authors did not compare cardiometabolic effects of both types of subcutaneous infusion.

The small number of studies and their numerous limitations make the question of cardiometabolic benefits with either MR-HC or 24-hour circadian subcutaneous infusion of hydrocortisone to remain still open, and should be investigated in future, well-designed, and larger-scale studies. It should be noted though that subcutaneous infusion of hydrocortisone is a cumbersome therapy and probably will be used clinically only in highly selected patients.

Another emerging treatment approach is the use of CRF-1 antagonists: crinecerfont and tildacerfont. In recently published phase 2 open-label studies, both drugs administered orally to patients with poorly controlled CAH attenuated the morning rise in ACTH and led to a decrease in 17OHP and androstenedione concentrations (285-287). Moreover, crinecerfont decreased testosterone concentrations in women and adolescents with C-CAH, and the androstenedione/testosterone ratio in men with C-CAH (285, 286). Recently, in a randomized controlled trial with crinecerfont vs placebo (n = 182), 63% of crinecerfont treated adult patients with C-CAH reached physiologic glucocorticoid dose compared with 18% in placebo group (288). Similar findings with crinecerfont were also found in children with C-CAH (289). These findings suggest that concomitant treatment with CRF-1 antagonists may help to decrease replacement doses of glucocorticoids from supraphysiologic to physiological (block/replacement therapy), and to reduce cardiometabolic risk in individuals with CAH. It is possibly that these agents administered together with glucocorticoids (and mineralocorticoids) may be beneficial in CAH treatment, particularly in patients with CAH showing cardiometabolic comorbidities (hypertension, obesity, or prediabetes). However, the exception may be concurrent administration of tildacerfont and dexamethasone resulting in increased exposure to dexamethasone, probably because of interactions between both drugs (287).

Very little is known about cardiometabolic effects of other treatment options used together with glucocorticoids in patients with CAH. One of the most promising options is agents targeting steroid production. Abiraterone acetate, the acetate prodrug of abiraterone, a selective and irreversible inhibitor of 17α-hydroxylase/17,20-lyase, was found to reduce androgen levels in individuals with CAH (290), and may theoretically allow tapering down glucocorticoid dose. Although deficiency of 17α-hydroxylase/17,20-lyase and administration of abiraterone acetate to patients with advanced prostatic cancer are associated with increased values of BP, no women with C-CAH receiving this drug developed hypertension (291). No change in BP is probably a consequence of the fact that patients with 21OHD have impaired conversion of progesterone to 11-deoxycorticosterone, preventing accumulation of 11-deoxycorticosterone and stimulation of the mineralocorticoid receptor (291). Another agent that may reduce glucocorticoid dose in patients with CAH is nevanimibe, a potent acyl-coenzyme A:cholesterol O-acyltransferase 1 inhibitor, which in a phase 2 study reduced 17-OHP concentrations and, though to a lesser extent, also androstenedione concentrations (292). Although there are no studies assessing cardiometabolic effects of nevanimibe in individuals with CAH, inhibition of acyl-coenzyme A:cholesterol O-acyltransferase 1 in diet-induced obese mice decreased weight, fat content, circulating levels of glucose, and triglycerides, and improved insulin sensitivity (293). Despite a stimulatory effect on lipolysis of visceral adipose tissue, in a retrospective analysis of 56 children with CAH, there was no differences in BMI *z*-score, total fat mass, visceral adipose tissue, and subcutaneous abdominal fat between patients receiving and not receiving anastrozole, an aromatase inhibitor used in the developmental age to delay bone maturation (294). In turn, possible unfavorable cardiometabolic effects of “medical adrenalectomy” by mitotane may be an argument (beyond teratogenicity and significant toxicity) against its use in patients with C-CAH, despite resulting in disappearance, volume reduction, or volume stabilization of testicular adrenal rest tumors and the improvement in sperm count (295). Administered to patients with adrenocortical cancer, this drug increases all main lipid fractions (296), and, by inducing cytochrome P450 3A4 enzymatic activity, its administration is associated with increased daily glucocorticoid dosing (297). In contrast, bilateral adrenalectomy, used in very selected patients with C-CAH, could theoretically improve cardiometabolic outcomes by controlling hyperandrogenism with physiological glucocorticoid doses but no studies to prove this exist (298).

Patients with NC-CAH often do not require glucocorticoid and mineralocorticoid replacement but frequently receive drugs with antiandrogen properties (268). In turn, women with C-CAH may require concomitant treatment with glucocorticoids and antiandrogens (1). The most commonly used treatment option for women with androgen excess are oral combined estrogen–progestin contraceptives. However, these agents used for pregnancy prevention increase the risk of venous thromboembolism and, though less frequently, are associated with arterial thrombotic complications and hypertension (299, 300). The relative risk of these complications depends on the type and dose of estrogen, the type of progestin, and the presence of concomitant risk factors (300). Unfortunately, cardiometabolic risk in users of oral combined estrogen–progestin contraceptives has been compared mainly with the risk in untreated young healthy women. Thus, these conclusions cannot be transferred to patients with concomitant disorders, assessed only in few studies. A deteriorating effect of ethinyl estradiol/drospirenone combination therapy in women with androgen excess (mainly caused by PCOS) was weaker than in healthy controls (301). However, in sexually active women with hyperprolactinemia wanting to avoid pregnancy, ethinyl estradiol plus desogestrel worsened insulin sensitivity and increased triglycerides, hsCRP, fibrinogen, and urinary albumin to creatinine ratio (302). Only 1 recent study including adolescent and young adult women with NC-CAH did not show changes in BMI SDS after replacing glucocorticoids with oral contraceptives or adding oral contraceptives to glucocorticoids (303). Spironolactone, used by some women with androgen excess, is a well-established treatment improving cardiovascular prognosis in individuals with hypertension, patients with heart failure and reduced ejection fraction, and in survivors of myocardial infarction with left ventricular dysfunction (304). However, its cardiometabolic safety has not been evaluated in conditions associated with androgen excess. Moreover, spironolactone is an aldosterone receptor antagonist, while patients with CAH have a various degree of aldosterone deficiency (305), sometimes even if they have NC-CAH (268, 306). Thus, at least theoretically, spironolactone does not appear to be a good choice. Lastly, androgen receptor antagonists and 5α-reductase inhibitors, either did not affect or slightly improved glucose homeostasis and/or the lipid profile in women with PCOS (307), but their cardiometabolic effects in individuals with CAH have not been yet investigated.

In summary, there are no convincing data that treatment with hydrocortisone preparations aimed at mimicking circadian cortisol rhythm is associated with lower cardiometabolic risk than conventional glucocorticoid preparations, as well as that cardiometabolic risk in CAH is reduced by hormonal drugs inhibiting androgen secretion and receptor action. However, this conclusion is based only on the results of a few studies with many drawbacks.

## Nonspecific Treatment and Prevention

Patients with CAH at high diabetes risk are assumed to benefit from the intensive lifestyle intervention and metformin treatment. Until now, only 4 case reports showed a reduction in body weight in individuals with CAH, and only 3 individuals were not previously diagnosed with diabetes (Table 7). Three-month treatment with small doses of metformin (500 mg twice daily) decreased body weight in an overweight 17-year-old girl with SW-CAH (269). Chronic topiramate treatment of an obese 17-year-old female with SW-CAH led to a sustained reduction in BMI, which was accompanied by a reduction in content of total body fat and visceral adipose tissue (270). Gastric sleeve surgery resulted in a dramatic weight loss, improved insulin sensitivity, decreased liver fat content, and caused a reduction in the daily hydrocortisone dose required to adequately control adrenal androgen production in a 19-year-old female with SW-CAH and morbid obesity (271). Finally, bariatric surgery (Roux-en-Y gastric bypass) reduced BMI and alleviated symptoms of androgen excess in a 39-year-old women with obesity and NC-CAH due to 11-hydroxylase deficiency, initially treated, because of coexisting type 2 diabetes, with metformin and pioglitazone (272).

Some precautions should be taken in case of physical activity in individuals with CAH. Despite no cases of overt hypoglycemia and glycopenic symptoms in the study by Green-Golan et al, individuals with C-CAH had lower glucose levels than controls during and shortly (15 minutes) after a standardized 90-minute moderate-intensity exercise that was comparable to brisk walking (80). The decrease in glucose levels, statistically significant already after 60 minutes, was a consequence of epinephrine deficiency as well as of the lack of adequate counterregulatory response (80). Thus, patients with CAH should be prepared before very high-intensive training with a high carbohydrate intake (eg, pasta). Moreover, if the very high-intensive training goes on for prolonged time, such as marathon, regular intake of high carbohydrate snacks is necessary. Extra hydrocortisone should also be used before prolonged exercise (eg, a marathon) (308).

Although in the general population, metformin, thiazolidinediones, α-glucosidase inhibitors and liraglutide have been found to lower the risk of diabetes in individuals with prediabetes (309), only pioglitazone has been used in a trial assessing glucose homeostasis in nondiabetic individuals with CAH. Administered at the daily dose of 45 mg for 16 weeks, pioglitazone improved insulin sensitivity and additionally reduced BP (94). Impaired insulin sensitivity suggests that individuals with CAH and type 2 diabetes should be treated with insulin sensitizers (metformin, thiazolidinediones), glucagon-like peptide 1 analogs, dipeptidyl peptidase-4 inhibitors, and sodium-glucose cotransporter 2 inhibitors rather than with insulin, sulfonylureas, and meglitinides. Only 1 preliminary study has evaluated the effectiveness and safety of pharmacotherapy of diabetes in patients with CAH. Six-month metformin treatment of women with NC-CAH and recently diagnosed type 2 diabetes decreased fasting glucose, HOMA-IR, HbA_1c_, and triglycerides to the same degree as in controls with type 2 diabetes and was well tolerated (95). Interestingly, metformin was reported to be the second most commonly used glucose-lowering drug (after insulin) in patients with C-CAH and concomitant type 2 diabetes (40%), and by far the most commonly prescribed agent (91%) in patients with C-CAH and hyperinsulinemia (163). However, patients with type 2 diabetes and established atherosclerotic cardiovascular disease or having indicators of high risk are currently recommended to receive a sodium-glucose cotransporter 2 inhibitor or glucagon-like peptide 1 receptor agonist with demonstrated cardiovascular disease benefits as part of the glucose-lowering regimen (310). Considering increased cardiovascular risk, these recommendations may theoretically apply also to subjects with CAH.

The current guidelines on cardiovascular disease prevention recommend lifestyle changes regarding diet and physical activity, cessation of smoking, reduction of alcohol intake, reduction of elevated BP and reaching target LDL cholesterol levels, which depend on age and the 10-year risk of atherosclerotic cardiovascular disease. Because of lack of net benefit, aspirin is rarely recommended in the routine primary prevention of atherosclerotic cardiovascular disease (311). The ongoing recommendations for patients with established atherosclerotic cardiovascular disease include lifestyle changes, smoking cessation, a target for SBP of 130 to 140 mmHg, and a target for LDL cholesterol below 1.8 mmol/L (70 mg/dL) (311). Observations by Falhammar et al justify prevention and treatment of heart, vascular, and metabolic complications also in NC-CAH (74), even if glucocorticoid replacement has not been used.

Statins significantly reduce the incidence of all-cause mortality and major coronary events in both the primary and secondary prevention (312). As mentioned above, individuals with CAH may benefit from statin therapy, which lowered total and LDL cholesterol levels, exerted pleiotropic effects, reduced androgen concentrations, and was well-tolerated (96, 97, 313). Although both statins and glucocorticoids may induce myopathy, no case of this complication have been reported in CAH, probably because of using relatively low statin and glucocorticoid doses. Moreover, although adrenal cortex hormones are mostly produced from cholesterol contained in LDLs, even aggressive statin therapy (resulting in very low LDL cholesterol levels) did not affect plasma cortisol and does not seem to induce AI in subjects with subnormal glucocorticoid production (314). There are no data in CAH on the effectiveness and safety of ezetimibe, proprotein convertase subtilisin/kexin 9 inhibitors, or bile acid sequestrants recommended to patients who either do not tolerate statins or do not achieve therapeutic goals with the maximum tolerated dose of a statin (311).

The knowledge is limited about antihypertensive agents in patients with CAH, and we can only speculate about the preferential treatment strategy. In case of mineralocorticoid overreplacement, salt intake should be restricted, and fludrocortisone dose should be minimized. First-line medications for hypertension include thiazide diuretics, β-adrenergic antagonists, calcium channel blockers, angiotensin-converting enzyme inhibitors (ACE-Is), and angiotensin receptor blockers (ARBs), and theoretically all these medications may be used in individuals with CAH (311). However, ACE-Is and ARBs may be the drugs of choice because of their cardioprotective effects. In addition to reducing BP, pharmacological inhibition of the renin–angiotensin system improves insulin sensitivity, normalizes endothelial function, reverses early atherosclerotic changes in the vascular wall, and improves left ventricle function (315, 316). If hypertension persists, a calcium antagonist can be added since these agents reduce the intima–media thickness progression rate and cause a significant reduction in the appearance of new angiographic lesions (317). Although β-adrenergic antagonists seem to induce negative metabolic effects, such as weight gain, glucose intolerance, and dyslipidemia, their use should be considered in patients with CAH after myocardial infarction (318). However, it appears that such patients should be treated with third generation drugs (carvedilol, nebivolol or labetalol), which have fewer side effects and a better metabolic profile than nonselective β-adrenergic antagonists (318). In the previous mentioned study by Righi et al, 10 out of 244 (4%) patients with C-CAH used antihypertensive drugs, the most commonly being ACE-Is, and calcium channel blockers, but also β-adrenergic antagonists, ARBs, and loop diuretics were used (163).

In summary, the results of small sample studies and case reports suggest that individuals with CAH may gain cardiometabolic benefits from treatment with metformin, statins, pioglitazone, topiramate, and bariatric surgery. Owing to the lack of supporting evidence and specific adverse effects, recommendations on the use of remaining treatment options (other antidiabetic and hypolipidemic drugs, hypotensive agents, and aspirin) are similar to those in the general population.

## Cardiometabolic Risk in CAH in Comparison With Other Forms of AI

**Figure 1.**
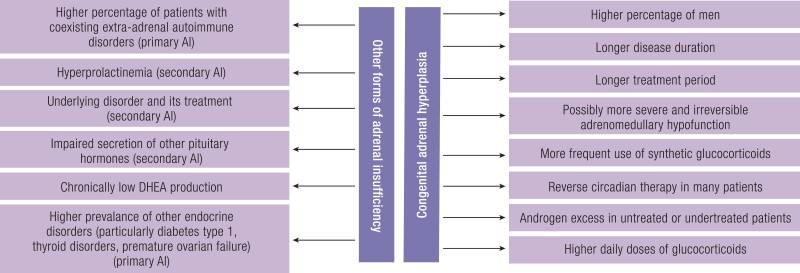
Factors putatively increasing cardiometabolic risk in congenital adrenal hyperplasia and in other forms of adrenal insufficiency.

No head to head comparisons make it difficult to compare the risk of cardiometabolic complications between patients with CAH and AI. However, increased mortality and cardiovascular morbidity observed in patients with CAH (72, 75, 87, 160, 161) seem to correspond to the results of 3 large population-based observational studies including patients with Addison disease (319-321). Although indirect comparisons between the results of epidemiological studies suggest general similarities between CAH and AI, it cannot be excluded that differences in mortality and cardiovascular morbidity between these disorders exist (Fig. 1).

Firstly, due to an autosomal recessive pattern of inheritance, prevalence of CAH is similar in men and women, while primary and secondary AI is diagnosed predominantly in females (322). Thus, the percentage of men is greater in populations of subjects with CAH than AI, and these differences in sex distribution and production of sex hormones across the lifespan may translate into different cardiometabolic risk (323).

Secondly, C-CAH is in the majority of cases diagnosed soon after birth, while NC-CAH in childhood, adolescence or early adulthood (13, 14). Autoimmune primary AI usually manifests between 30 and 50 years of age, while secondary AI is diagnosed most frequently in the sixth decade of life (322). Thus, the period for glucocorticoid and fludrocortisone replacement is longer for C-CAH than AI. Moreover, treatment of CAH is aimed not only at compensating impaired function of the adrenal cortex, but also at reducing androgen production (2, 22). Consequently, compared with AI, patients with CAH are frequently treated with supraphysiological doses of glucocorticoids, more often receive longer-acting synthetic glucocorticoids, and, to better suppress the early morning rise in ACTH, glucocorticoids in this population are frequently given in a reverse circadian pattern, with the highest dose in the evening (324). Moreover, due to the presence of precursors with antimineralocorticoid properties (17OHP and progesterone), patients with SW-CAH, particularly poorly controlled, often require higher daily doses of fludrocortisone than patients with primary AI (277). Higher daily doses and a longer average treatment period result in higher cumulative doses of glucocorticoids and often also of mineralocorticoids in CAH than AI. Moreover, cortisol deficiency in CAH already in the prenatal period may impair the development of epinephrine-secreting cells, as well as autocrine, paracrine, and endocrine interactions between the adrenal cortex and adrenal medulla to a greater degree than acquired AI (325). However, cardiometabolic consequences of putative differences in adrenal medulla function remain unknown.

Thirdly, over 80% of all cases of Addison disease in developed countries is caused by autoimmune adrenalitis, and more than 60% of patients with this form of AI show clinical or preclinical signs of another autoimmune disease (326). In turn, prevalence of concomitant autoimmune disorders in persons with 21OHD has been estimated at 7.4% to 22.2% and has been found increased particularly in individuals aged 40 years and older, males, and subjects with milder phenotypes (75, 327). A greater risk of autoimmune disorders in autoimmune primary AI than CAH may translate into greater cardiometabolic risk. In a large population-based study, the incidence rate of cardiovascular disease was higher in patients with at least 1 autoimmune disorder and increased with the number of autoimmune diseases (321). Patients with nonendocrine autoimmune disorders more frequently also develop type 2 diabetes and metabolic syndrome (328). Moreover, although CAH may be complicated by thyroid dysfunction (327) and rarely by premature ovarian failure (1), the list of disorders coexisting with autoimmune primary AI that may lead to cardiometabolic complications is longer and includes type 1 diabetes mellitus, thyroid dysfunction, premature ovarian failure, celiac disease, hypoparathyroidism, hepatitis, atrophic gastritis, and low vitamin B_12_ (326); while in secondary AI—the causative factor and nontreated or imperfectly treated deficiencies of other pituitary hormones (particularly growth hormone deficiency and hypogonadotropic hypogonadism) may increase the cardiometabolic risk (329).

Lastly, there are remarkable differences in the androgen profile between patients with AI and CAH. Individuals with AI have low concentrations of dehydroepiandrosterone (DHEA) and DHEA-S (unless they receive exogenous DHEA preparations), while concentrations of testosterone and androstenedione are determined by gonadal function, being very low in many patients with secondary AI and in postmenopausal women with autoimmune primary AI (203). Patients with CAH represent an even more heterogeneous group, because androgen concentrations in CAH depend on its severity, disease control and intensity of treatment (21). Moreover, androgen concentrations may fluctuate to a greater degree in patients with CAH than in healthy subjects (1).

In summary, both CAH and other forms of AI are associated with the excess mortality rate and an increase prevalence of cardiovascular disease. Despite theoretical premises, the lack of observational studies comparing clinically definitive or, at least, surrogate endpoints preclude drawing firm conclusions about whether cardiometabolic risk in these disorders is similar or higher in 1 of them. Future targeted studies are warranted to answer this question, which seems to be important from a prognostic and therapeutic point of view.

## Conclusions and Further Research

**Figure 2.**
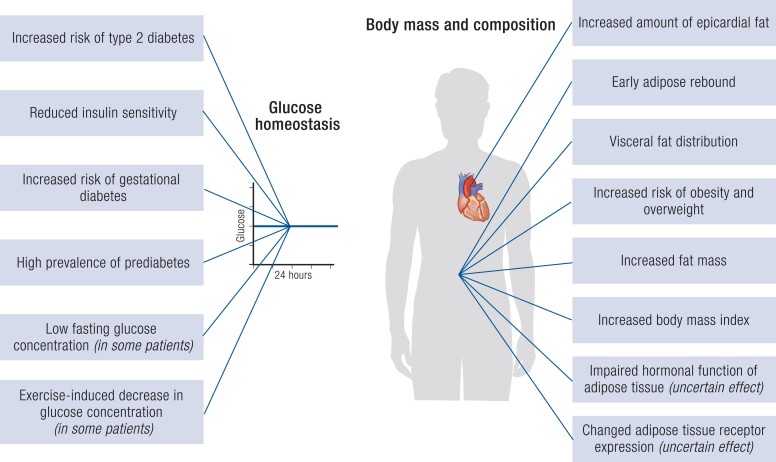
Metabolic complications of congenital adrenal hyperplasia.

**Figure 3.**
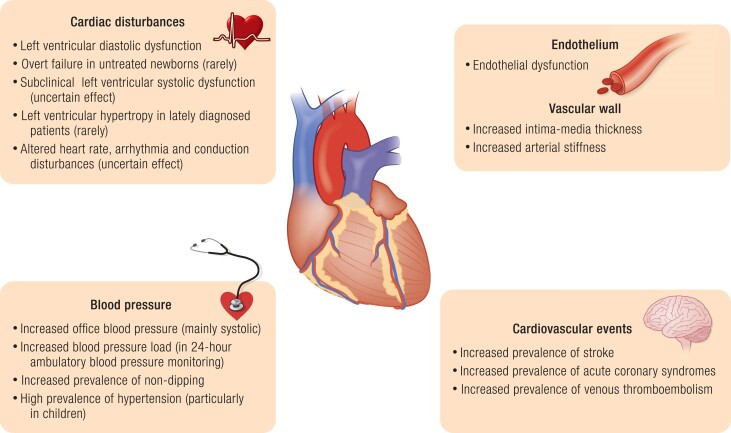
Cardiovascular complications of congenital adrenal hyperplasia.

**Figure 4.**
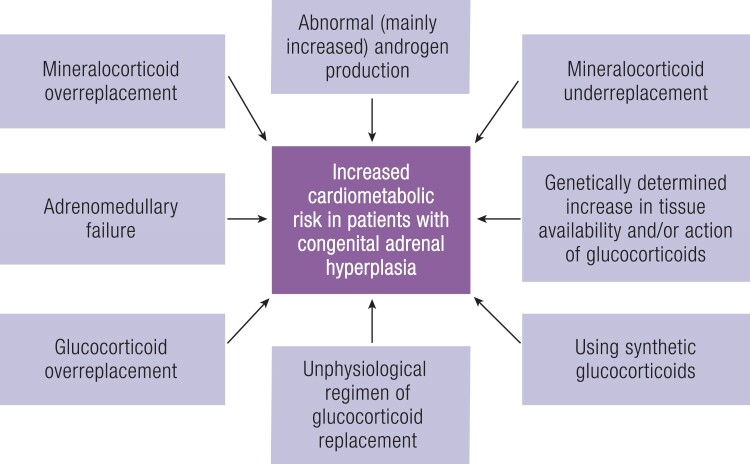
Potential mechanisms increasing cardiometabolic risk in patients with congenital adrenal hyperplasia.

Individuals with C-CAH often have a constellation of metabolic abnormalities, increased values of BP, endothelial dysfunction, and structural changes in the vascular wall (Figs. 2 and 3). Inconsistent results of individual studies may be explained by the presence of many different mechanisms increasing cardiometabolic risk in CAH (Fig. 4). In line with this explanation, the analyzed groups differed in phenotype, genotype, treatment, and the level of hormonal control. The reported absolute number of patients with CAH developing definitive, clinically relevant endpoints was relatively low. Nevertheless, outcome data for cardiovascular mortality and morbidity were obtained from national registers based on disease coding, and the available data are biased by overrepresentation of young patients. Similarly, most studies assessing modifiable cardiometabolic risk factors included only or mainly children and young adults. However, the incidence of cardiovascular disease, metabolic syndrome, and type 2 diabetes increases with age, and most cardiovascular events, even in high-risk populations, require years to develop. This justifies the need to design and conduct future studies assessing cardiometabolic risk in middle-aged and older adults with CAH.

At physiologic doses, hydrocortisone or synthetic glucocorticoids replace the deficient adrenocortical cortisol secretion and prevent acute adrenal crises (330) but do not suppress ACTH and adrenal androgen production. The complex pathophysiology of CAH makes it more difficult to treat than other forms of AI, and currently there is no ideal glucocorticoid regimen (22). Moreover, because of the lack of reliable markers of glucocorticoid action, adequacy of glucocorticoid replacement therapy is monitored mainly based on careful clinical assessment (212, 331). Although concentrations of 17OHP and adrenal androgens can be used as an indirect measurement of glucocorticoid treatment, this strategy is not ideal (2). Conventional therapy does not mimic the physiological rhythm of cortisol secretion, inevitably resulting in periodic overreplacement and/or underreplacement. These treatment inaccuracies may have a negative impact on cardiometabolic health. This highlights the need for wider use of glucocorticoid preparations exhibiting pharmacokinetic and pharmacodynamic profiles which get closer to the physiological circadian cortisol secretion. New formulations of hydrocortisone with modified-release characteristics and new delivery formats at least theoretically allow for a more physiological replacement, and together with better monitoring may lead to better clinical outcomes for subjects with CAH.

The complex etiology of excess cardiovascular and metabolic morbidity in CAH may potentially be explained by a U-shaped relationship between cardiovascular complications and androgen concentrations, which means that both low and high concentrations of these hormones predispose to increased cardiometabolic risk. Although there are no direct data concerning patients with CAH, such a relationship has been documented in older adult males and females participating in large population studies (332-334). This relationship may be masked by genetic variations in steroid hormone secretion and androgen receptor action, and by greater interindividual differences in androgen production compared with the general population, limiting the utility of plasma/serum measurements of 17OHP, DHEA-S, androstenedione, testosterone, and dihydrotestosterone as markers predictive of subsequent development of cardiometabolic complications. Moreover, it cannot be excluded that 21-deoxycortisol, 11-oxygenated androgens, and intermediates of the androgen backdoor pathway may better reflect adequate hormonal control of CAH than traditional biomarkers.

Although severe forms of CAH are accompanied by adrenomedullary failure, its causative role in the development of cardiometabolic complications of CAH has been assessed only in few studies, and this role requires better understanding.

It is also vital to better investigate cardiometabolic aspects of NC-CAH, which is by far the most common form of CAH. Although cardiometabolic risk was found to be increased in young symptomatic females, almost nothing is known about this risk in young asymptomatic women, postmenopausal women, and males. Future studies are needed to ascertain whether NC-CAH poses any increased risks for adverse cardiovascular/cardiometabolic outcomes.

Of growing interest is a possible association between cardiometabolic risk and complications in CAH and treatment adherence of patients. This term involves not only taking the required dose of a drug at the correct time, but also avoiding forbidden concomitant drugs and complying with nonpharmacological interventions. The term “adherence” emphasizes active choice of a patient taking responsibility for own well-being, which is a very important component of management (335). Nonadherence with glucocorticoid therapy in patients with CAH was found to increase with age and was reported in over one-third of adults (87, 336). Poor adherence in CAH was associated with negative health outcomes: increased thickness of intima–media (110), and impaired quality of life (337). Moreover, poorly adherent patients constituted about one-third of patients with CAH participating in a study that showed a younger mean age at death in individuals with CAH than in controls (87). Unfortunately, treatment adherence is not well reflected by steroid measurements. Ekbom et al reported a 31% discordance rate between self-reported medication adherence and medication adherence assessed by physicians on the basis of medical history, growth rate, and steroid concentrations (336). This discordancy was observed though the authors analyzed 24-hour 17OHP profiles, which seem to be more accurate than singular measurements.

Lastly, it seems justified to consider partial updating the current recommendations of the Endocrine Society concerning surveillance for long-term complications in CAH that came into force in 2018 (2). The guidelines “suggest introducing counseling regarding healthy lifestyle choices at an early age to maintain BMI within the normal range to avoid metabolic syndrome and related sequelae (point 6.10),” as well as “recommend against routine evaluation for cardiac and metabolic disease in patients with CAH beyond that recommended for the general population (point 6.14).” In our opinion, routine counseling concerning lifestyle choices should be part of the follow-up of each patient with CAH. All necessary questions should be discussed with the parents, and with the affected child when able to comply. Moreover, we advise that all individuals with C-CAH require metabolic and cardiovascular follow-up. For adults with C-CAH we would suggest BMI, waist circumference (and/or the waist to hip ratio), plasma glucose, HbA1c, plasma lipids, heart rate, and BP measured yearly. Selected patients with C-CAH may require dual-energy X-ray absorptiometry (or bioelectrical impedance if not available) to estimate body composition (particularly body fat), a standard 2-hour 75 g glucose oral tolerance test, 24-hour ambulatory BP monitoring, and/or cardiovascular testing (measurement of intima–media thickness, exercise electrocardiogram, and/or echocardiography). Until more data are available, the current less rigorous diagnostic and preventive approach of the Endocrine Society should still be recommended for individuals with NC-CAH. Although increased risk for cardiometabolic disease in NC-CAH remains unproven, healthy lifestyle mentioned in the guidelines has been documented to bring health benefits to the general adult population, and patients with mild forms of CAH are also likely to benefit from this approach (338, 339).

## Abbreviations

17OHP: 17-hydroxyprogesterone
21OHD: 21-hydroxylase deficiency
ACE-I: angiotensin-converting enzyme inhibitor
ACTH: adrenocorticotropic hormone
AI: adrenal insufficiency
ARB: angiotensin receptor blocker
BMI: body mass index
BP: blood pressure
CAH: congenital adrenal hyperplasia
C-CAH: classic congenital adrenal hyperplasia
CRF-1: corticotropin-releasing factor type 1 receptor
CRP: C-reactive protein
DBP: diastolic blood pressure
DHEA: dehydroepiandrosterone
DHEA-S: dehydroepiandrosterone-sulphate
HbA_1c_: glycated hemoglobin
HDL: high-density lipoprotein
HOMA-IR: homeostatic model assessment for insulin resistance index
hsCRP: high-sensitivity C-reactive protein
LDL: low-density lipoprotein
MR-HC: modified-release formulation of hydrocortisone
NC-CAH: nonclassic congenital adrenal hyperplasia
PCOS: polycystic ovary syndrome
SBP: systolic blood pressure
SDS: standard deviation score
SR-HC: sustained-release formulation of hydrocortisone
SV-CAH: simple virilizing congenital adrenal hyperplasia
SW-CAH: salt-wasting congenital adrenal hyperplasia
